# Targeting *Leishmania infantum* Mannosyl-oligosaccharide glucosidase with natural products: potential pH-dependent inhibition explored through computer-aided drug design

**DOI:** 10.3389/fphar.2024.1403203

**Published:** 2024-05-30

**Authors:** Luis Daniel Goyzueta-Mamani, Haruna Luz Barazorda-Ccahuana, Mayron Antonio Candia-Puma, Alexsandro Sobreira Galdino, Ricardo Andrez Machado-de-Avila, Rodolfo Cordeiro Giunchetti, José L. Medina-Franco, Mónica Florin-Christensen, Eduardo Antonio Ferraz Coelho, Miguel Angel Chávez-Fumagalli

**Affiliations:** ^1^ Computational Biology and Chemistry Research Group, Vicerrectorado de Investigación, Universidad Católica de Santa María, Arequipa, Peru; ^2^ Facultad de Ciencias Farmacéuticas, Bioquímicas y Biotecnológicas, Universidad Católica de Santa María, Arequipa, Peru; ^3^ Laboratório de Biotecnologia de Microrganismos, Universidade Federal São João Del-Rei, Divinópolis, Brazil; ^4^ Programa de Pós-Graduação em Ciências da Saúde, Universidade do Extremo Sul Catarinense, Criciúma, Brazil; ^5^ Laboratório de Biologia das Interações Celulares, Instituto de Ciências Biológicas, Universidade Federal de Minas Gerais, Belo Horizonte, Brazil; ^6^ Instituto Nacional de Ciência e Tecnologia de Doenças Tropicais (INCT-DT), Salvador, Brazil; ^7^ DIFACQUIM Research Group, Department of Pharmacy, School of Chemistry, Universidad Nacional Autónoma de México, Mexico City, Mexico; ^8^ Instituto de Patobiología Veterinaria, CICVyA, Instituto Nacional de Tecnología Agropecuaria (INTA), Buenos Aires, Argentina; ^9^ Consejo Nacional de Investigaciones Científicas y Técnicas (CONICET), Buenos Aires, Argentina; ^10^ Programa de Pós-Graduação em Ciências da Saúde: Infectologia e Medicina Tropical, Faculdade de Medicina, Universidade Federal de Minas Gerais, Belo Horizonte, Brazil; ^11^ Departamento de Patologia Clínica, Colégio Técnico da Universidade Federal de Minas Gerais (COLTEC), Universidade Federal de Minas Gerais, Belo Horizonte, Brazil

**Keywords:** drug discovery, natural products, Ocotillone, Subsessiline, visceral leishmaniasis, molecular docking simulation, molecular dynamics simulation, virtual screening

## Abstract

Visceral Leishmaniasis (VL) is a serious public health issue, documented in more than ninety countries, where an estimated 500,000 new cases emerge each year. Regardless of novel methodologies, advancements, and experimental interventions, therapeutic limitations, and drug resistance are still challenging. For this reason, based on previous research, we screened natural products (NP) from Nuclei of Bioassays, Ecophysiology, and Biosynthesis of Natural Products Database (NuBBE_DB)_, Mexican Compound Database of Natural Products (BIOFACQUIM), and Peruvian Natural Products Database (PeruNPDB) databases, in addition to structural analogs of Miglitol and Acarbose, which have been suggested as treatments for VL and have shown encouraging action against parasite’s N-glycan biosynthesis. Using computer-aided drug design (CADD) approaches, the potential inhibitory effect of these NP candidates was evaluated by inhibiting the Mannosyl-oligosaccharide Glucosidase Protein (MOGS) from *Leishmania infantum*, an enzyme essential for the protein glycosylation process, at various pH to mimic the parasite’s changing environment. Also, computational analysis was used to evaluate the Absorption, Distribution, Metabolism, Excretion, and Toxicity (ADMET) profile, while molecular dynamic simulations were used to gather information on the interactions between these ligands and the protein target. Our findings indicated that Ocotillone and Subsessiline have potential antileishmanial effects at pH 5 and 7, respectively, due to their high binding affinity to MOGS and interactions in the active center. Furthermore, these compounds were non-toxic and had the potential to be administered orally. This research indicates the promising anti-leishmanial activity of Ocotillone and Subsessiline, suggesting further validation through *in vitro* and *in vivo* experiments.

## 1 Introduction

Leishmaniasis is a vector-borne disease caused by *Leishmania spp.* protozoan parasites. It is transmitted to humans through the bites of infected female sandflies of *Phlebotomus spp.* (Old World) and *Lutzomyia spp.* (New World), with distinct clinical manifestations. With a staggering impact affecting over 12 million individuals worldwide across 90 countries, particularly afflicting economically disadvantaged regions, prominent in nations like Brazil, Ethiopia, Sudan, South Sudan, India, and Bangladesh where sandfly vectors flourish (Alvar et al., 2012; Akhoundi et al., 2016), Leishmaniasis remains a neglected tropical disease of critical concern. The disease showcases three primary clinical forms: cutaneous (CL), mucosal (ML), and visceral leishmaniasis (VL); this last one is caused mainly by *L. infantum*, resulting in severe symptoms including anemia, fever, weight loss, and an enlargement of the liver and spleen, among other internal organs and bone marrow (Ghazanfar and Malik, 2016). Endemic in 99 countries, Cutaneous Leishmaniasis (CL) prevails in 89 nations, while Visceral Leishmaniasis (VL) is observed in 80 countries, emphasizing the global burden of this disease (Hailu et al., 2016).

Despite its widespread prevalence, therapeutics for leishmaniasis are limited, with antimonials, single and liposomal amphotericin B, and miltefosine being the most common treatments (Bernal and Coy-Barrera, 2014). However, these drugs often exhibit severe side effects, such as the risk of harming healthy cells and high treatment costs, raising concerns about medication resistance in *Leishmania* parasites. Antimonials’ resistance has compromised the efficacy of these drugs, leading to a pressing need for alternative treatments (Freitas-Junior et al., 2012; Ponte-Sucre et al., 2017). This context highlights the urgency of exploring new avenues for effective and safer treatments for leishmaniasis. On the other hand, developing a vaccine for leishmaniasis has proven to be challenging due to the parasite’s diversity and the host’s immunological response. Despite this challenge, ongoing research in various areas, such as nanomedicine, biomarkers research, and drug repurposing, reveals information on potential treatment alternatives (Kaye et al., 2021).

Researchers are actively working to repurpose current medications and identify accurate biomarkers to monitor treatment response and predict recurrence (Rostami and Khamesipour, 2021; Volpedo et al., 2021). The oral drugs, Miglitol and Acarbose, initially designed for treating type-2 diabetes, are examples of such repurposed drugs. Classified as alpha-glucosidase inhibitors, these drugs function by inhibiting the intestinal digestion and absorption of carbohydrates (Ueno et al., 2015). Notably, Acarbose and Miglitol have demonstrated effectiveness against macrophages infected with *Leishmania*, impacting a key protein in the metabolic network of the N-glycan biosynthesis pathway crucial for the parasite’s survival (Chavez-Fumagalli et al., 2019). Particularly, Acarbose impaired the mitochondrial function in *L. infantum* parasites, leading to increased reactive oxygen species (ROS) production and, thus, cellular stress. This alteration induced a specific production of Th1-type cytokines, reducing parasitism in diverse targeted organs. *In vivo* treatment of Acarbose also elevated neutral lipid levels, indicating cell damage and parasite death (Costa et al., 2021).

Alternatives for drug development must be researched further to determine their effectiveness. Two critical factors must guide this research: differentiation from the mammalian host and the essential factor of the chosen target for the pathogen’s survival. A parasite’s survival depends on critical metabolic pathways governed by complex chemical mechanisms. Specific enzymes efficiently catalyze a variety of responses in these pathways (Kaye and Scott, 2011). These factors should be carefully considered to identify suitable targets that can effectively disrupt the *Leishmania* survival mechanisms. Research in the field of leishmaniasis focused on uncovering novel biochemical targets associated with various defense mechanisms, including RNA and DNA metabolism, glucose metabolism, sterols, fatty acids, the purine pathway, and nucleotides. The primary objective is identifying targets that new anti-leishmania drugs can effectively damage while ensuring the host’s safety (Jain and Jain, 2018; Raj et al., 2020; Soni and Pratap, 2022).

Consequently, there is an ongoing effort to discover novel inhibitors of proteins and enzymes that function as targets for anti-leishmanial resources. Notably, plant-derived flavonoids, including lupeol, quercetin, and gallic acid, have shown promising activity at comparatively low concentrations against Try-R and Try-S disulfide oxidoreductase enzymes crucial for the detoxification and survival of *Leishmania* (Mehwish et al., 2019). A significant *in vitro* efficacy against *Leishmania* promastigotes and amastigotes and the promising *in vivo* activity of sesquiterpene-related compounds derived from natural sources have been shown (Bernal and Coy-Barrera, 2014). A recent study used molecular docking techniques to analyze numerous terpenoids within the active sites of 24 enzymes from *L. major, L. donovani, L. mexicana,* and *L. infantum* (Ogungbe et al., 2014). Specific enzymatic targets have been studied and tested in this context to develop new drugs to treat leishmaniasis. Examples include Adenine phosphoribosyltransferase (APRT) (Barazorda-Ccahuana et al., 2024), where three furoquinolone alkaloids from the plant *Almeidea rubra* showed APRT inhibitory activity (Ambrozin et al., 2005); N-myristoyltransferase (NMT), in which phenolic compounds, including aurones, chalcones, lignans, and isoflavonoids, showed specific effect and docking activity against NMT (Ogungbe et al., 2014); Dihydroorotate dehydrogenase, where chalcones, flavonoids, monoterpenoids, limonoids, demonstrated anti-leishmanial effect (Ogungbe and Setzer, 2013); and Arginase, in which quercetin, epigallocatechin-3-gallate, and gallic acid exhibited the most inhibitory effects (dos Reis et al., 2013; Manjolin et al., 2013).

Given the potential of targeting diverse enzymes, exploring less-studied proteins presents an intriguing avenue for anti-leishmanial drug development. One such protein is Mannosyl-oligosaccharide glucosidase (MOGS). Initially, cathegorized as a hypothetical protein due to insufficient characterization. MOGS gained recognition after a thorough biochemical analysis confirmed its activity (Nairn and Moremen, 2014). This enzyme plays a crucial in the endoplasmic reticulum (ER) N-linked glycosylation pathway (Kuribara and Totani, 2022). The hydrolytic removal of the terminal α-1,2-linked glucose residue from the Glc3Man9GlcNAc2 oligosaccharide precursor is catalyzed at that location. This critical trimming step impacts glycoprotein trafficking, ER quality control mechanisms, and protein folding (Ito et al., 2020; Kuribara and Totani, 2021; Ninagawa et al., 2021).

*Leishmania*, parasites exhibit N-glycosylation patterns distinct from their mammalian hosts. MOGS is involved in these pathways, and research suggests that it contributes to the biosynthesis of Lipophosphoglycan (LPG), an essential defense mechanism for the promastigote stage of *Leishmania* that guarantees the parasite’s survival within the sandfly vector (Lodge and Descoteaux, 2005). Additionally, interactions between macrophage receptors and MOGS-processed parasite surface glycoproteins may contribute to *Leishmania’s* invasion of the host cell (Olafson et al., 1990).

Harnessing its extraordinary biodiversity and longstanding knowledge of medicinal plants, Latin America emerges as a powerful force in drug discovery. A recent development, the Latin American Natural Products Database (LANaPDB), integrates data from existing regional medicinal plant databases (Gómez-García et al., 2023). This comprehensive resource empowers researchers to identify promising natural product leads for diseases like Leishmaniasis, accelerating the path towards novel therapies. Computer-aided drug design (CADD) can further accelerate this process by facilitating the screening and analysis of these compounds, ultimately streamlining the search for novel therapies.

Computer-aided drug design (CADD) can facilitate the screening and analysis of these compounds, streamlining the search for novel anti-leishmanial therapies (Barazorda-Ccahuana et al., 2023b). Interestingly, environmental factors such as pH can influence both *Leishmania*’s life cycle and gene expression. Therefore, investigating the pH-dependent activity of natural products against MOGS could reveal new and potent anti-leishmanial compounds.

While natural and synthetic products hold promise, their exact mechanisms of anti-leishmanial action often remain elusive. Environmental factors, such as pH, temperature, and osmotic pressure, may influence the parasite’s life cycle and gene expression. For example, the ideal temperature for parasite species to proliferate as amastigote forms in mammalian hosts is around 37°C (Clos et al., 2022). Differentiation from promastigotes to amastigotes occurs during macrophage phagocytosis in an acidic environment, which can also occur between 4.5 and 6, although some species can adapt to values between 7 and 7.5. These pH fluctuations impact DNA, protein synthesis, and glucose metabolism (Antoine et al., 1990; Garlapati et al., 1999).

In this work, we aim to screen and evaluate compounds contained in three Latin American NP databases, the Nuclei of Bioassays, Ecophysiology, and Biosynthesis of Natural Products Database (NuBBE_DB_), BIOFACQUIM, and PeruNPDB at different pH levels to identify compounds whose properties and actions are comparable to those of Miglitol and Acarbose. After docking these compounds with the MOGS enzyme of *L. infantum*, their potential toxic effects were explored with molecular dynamic simulation analyses. This study promotes the research of innovative enzyme inhibitors, consequently expanding the field of medication discovery targeting leishmaniasis, and offers important insights at the molecular level. The insights provided by these analyses suggest the potential for advancing medicines constructed from natural products.

## 2 Materials and methods

### 2.1 Natural products ligand library and structural analog search

The simplified molecular-input line-entry system (SMILES) (Weininger, 1988) of NPs previously described were retrieved from NuBBE_DB_ online web server (version 2017) (https://nubbe.iq.unesp.br/portal/nubbe-search.html, accessed on 15 August 2023), which contains the information of more than 2,681 NPs and derivatives from Brazilian biodiversity (Pilon et al., 2017); BIOFACQUIM database (https://figshare.com/articles/dataset/BIOFACQUIM_V2_sdf/11312702., accessed on 15 August 2023, which contains the information of 395 NPs from Mexican biodiversity (Pilón-Jiménez et al., 2019); and from the Peruvian Natural Products Database (PeruNPDB) online web server (https://perunpdb.com.pe/, accessed on 15 August 2023), which contains the information of 280 NPs from Peruvian biodiversity (Barazorda-Ccahuana et al., 2023b).

Also, the SMILES from Acarbose (PubChem CID: 41774) and Miglitol (PubChem CID: 441314) were used for high throughput screening to investigate structural analogs by the SwissSimilarity server (http://www.swisssimilarity.ch/index.php, accessed on 15 September 2023) (Bragina et al., 2022); as the most similar from the commercial types of compounds and the zinc-drug-like compound, library databases were selected, while the combined screening method was chosen for the high throughput screening to achieve the best structural analogs. Default parameters chose threshold values for positivity.

### 2.2 Molecular properties calculation

The Osiris DataWarrior v05.02.01 software (Sander et al., 2015) was employed to generate the dataset’s structure data files (SDFs). This followed the uploading to the Konstanz Information Miner (KNIME) Analytics Platform (Fillbrunn et al., 2017), where the “Lipinski’s Rule-of-Five” node was employed to calculate physicochemical properties of therapeutic interest—namely: molecular weight (MW), octanol/water partition coefficient (clogP), number of H-bond donor atoms (HBD) and number of H-bond acceptor atoms (HBA)—of the dataset. To generate a visual representation of the chemical space of the dataset for the auto-scaled properties of pharmaceutical interest, the principal component analysis (PCA), which reduces data dimensions by geometrically projecting them onto lower dimensions called principal components (PCs), calculated by the “PCA” KNIME node. Three-dimensional scatter-plot representations were generated for PCA with the Plotly KNIME node.

### 2.3 Virtual screening

The FASTA sequence of the *L. infantum* Mannosyl-Oligosaccharide Glucosidase protein (MOGS) (ID: A4I3U9) was retrieved from the UniProt database (http://www.uniprot.org/), accessed on 03 October 2023), and subjected to automated modeling in SWISS-MODEL (Biasini et al., 2014). Moreover, various tools were used to analyze protein structure. The Ramachandran plot was carried out using the PROCHECK program of the SAVES program (v.6.0) (Laskowski et al., 1996) to validate the structure of the modeled 3D structure of the MOGS protein. Additionally, the ProSA-web program (Wiederstein and Sippl, 2007) was used to determine the Z-score of the modeled structure and the local model quality assessment for structural assessment.

The NP datasets were imported into OpenBabel using the Python Prescription Virtual Screening Tool (Dallakyan and Olson, 2015) and were subjected to energy minimization. PyRx performs structure-based virtual screening by applying docking simulations using the AutoDock Vina tool (Trott and Olson, 2010). The MOGS model was uploaded as a macromolecule, and a thorough search was carried out by enabling the “Run AutoGrid” option, which creates configuration files for the grid parameter’s lowest energy pose, and then the “Run AutoDock” option, which uses the Lamarckian GA docking algorithm. To ensure a comprehensive exploration of potential binding sites, blind docking was employed. The entire set of modeled 3D models was used as the search space for the study with this approach. The docking simulation was then run with an exhaustiveness setting of 20 and instructed only to produce the lowest energy pose. The statistical analysis was done within the GraphPad Prism software version 10.0.2 (232) for Windows from GraphPad Software, San Diego, California, United States, at http://www.graphpad.com. Violin plots were generated for visualization, and the One-way ANOVA followed by Dunnett correction for multiple comparisons test was employed to evaluate the differences between the datasets. The results were considered statistically significant when *p* < 0.05. For the selected compounds, the Tanimoto similarity score was calculated for clustering. The atom-pair-based fingerprints of the compounds were obtained using the “ChemmineR” package (Cao et al., 2008) in the R programming environment (version 4.0.3) (Dalgaard, 2010), and heatmaps were generated for visualization.

### 2.4 ADMET predictions

The pkCSMonline server, which uses graph-based signatures, was employed for the prediction of the compounds’ ADMET (absorption, distribution, metabolism, elimination, and toxicity) properties (https://biosig.lab.uq.edu.au/pkcsm/, accessed on 14 December 2023) (Pires et al., 2015). The Z-score was calculated for numerical results, while categorical data was converted into binary “Yes” or “No” data. Heatmaps were generated for visualization within the GraphPad Prism software version 10.0.2 (232) for Windows from GraphPad Software, San Diego, California, United States, at http://www.graphpad.com.

### 2.5 Protonation/deprotonation states by SGCMC

The protein structure input files were established using homology modeling via the Swiss-Model server (https://swissmodel.expasy.org, accessed in July 2023). The original protein was prepared to assess the impact of protonation/deprotonation states of titratable residues at pH 5 and pH 7 using the program developed by Barazorda-Ccahuana et al. (2021) (accessible online at https://github.com/smadurga/Protein-Protonation). The pKa value for each titratable residue (Asp, Glu, Arg, Lys, and His), along with the C-terminal and N-terminal ends, was determined using Propka v.3 (Rostkowski et al., 2011).

### 2.6 Molecular docking

The selected compounds underwent design and editing using the Avogadro v.1.2.0 software (Hanwell et al., 2012), followed by the automatic input generation CHARMM-GUI server (https://www.charmm-gui.org, accessed in September 2023)(Jo et al., 2008; Brooks et al., 2009). Before conducting docking, multiple druggable regions of the target protein were delineated, with the optimal druggable site identified using the PASSer program, which locates potential binding pockets within a protein’s structure based on their size, shape, and chemical properties and analyzes these pockets for “druggability,” comparing them to known drug-binding sites for similarity and ranks, up to 100%, the pockets based on their overall potential (https://passer.smu.edu, accessed in September 2023). Molecular docking was conducted using the free web server Dockthor, which performs protein-ligand docking using a genetic algorithm. It prepares the protein and ligand, then iteratively explores different binding poses, scoring them with the MMFF94s force field. The final output is a ranked list of potential binding modes for analysis, docking grids centered on the druggable region. (https://dockthor.lncc.br/v2/, accessed in October 2023) (Santos et al., 2020).

### 2.7 Molecular dynamics

The all-atom simulations were conducted using the Gromacs 2023 (Van Der Spoel et al., 2005) (Software package employing the Charmm27 (MacKerell et al., 2000) force field. Each system was enclosed within a cubic box measuring 10 nm³. Explicit water molecules were modeled using the TIP3P (Mark and Nilsson, 2001) water model, and ions were incorporated to achieve system neutrality. The molecular dynamics (MD) simulations comprised three primary stages. Firstly, energy minimization was performed by employing the steep integrator over 200,000 steps. Subsequently, the equilibrium simulation proceeded, wherein restrictions were imposed on the protein-ligand complex for 1 ns within the NVT (number of molecules, volume, and temperature constant) ensemble, followed by an additional restriction in the isobaric-isothermal NPT (number of molecules, pressure, and temperature constant) ensemble for 1 ns. Lastly, the production simulation phase commenced, where restrictions were lifted from the entire system for 100 ns under the isobaric-isothermal NPT ensemble. Estimating binding free energies between the proteins and the ligands was conducted using the Molecular Mechanics/Poisson-Boltzmann Surface Area (MM/PB(GB)SA) methodology. This approach was implemented via the tool called gmx_MMPBSA (Valdés-Tresanco et al., 2021), which was designed to bridge the gap and integrate the extensive functionalities of AmberTools’ MMPBSA.py and associated programs for GROMACS users.

**FIGURE 1:**
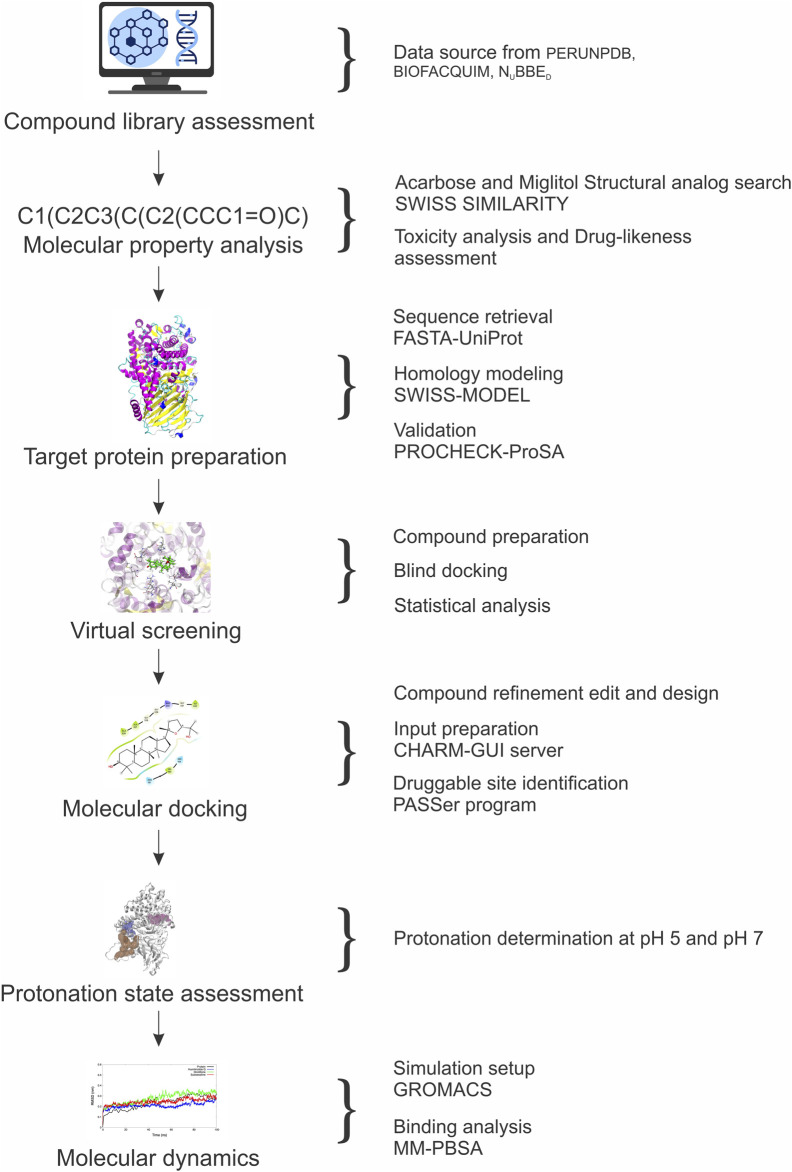
Flowchart summarizing the *in silico* analysis methods performed in this work.

Figure 1 provides a visual flowchart of the methodology performed.

## 3 Results

### 3.1 Natural products ligand library, chemical space visualization, and virtual screening

**FIGURE 2:**
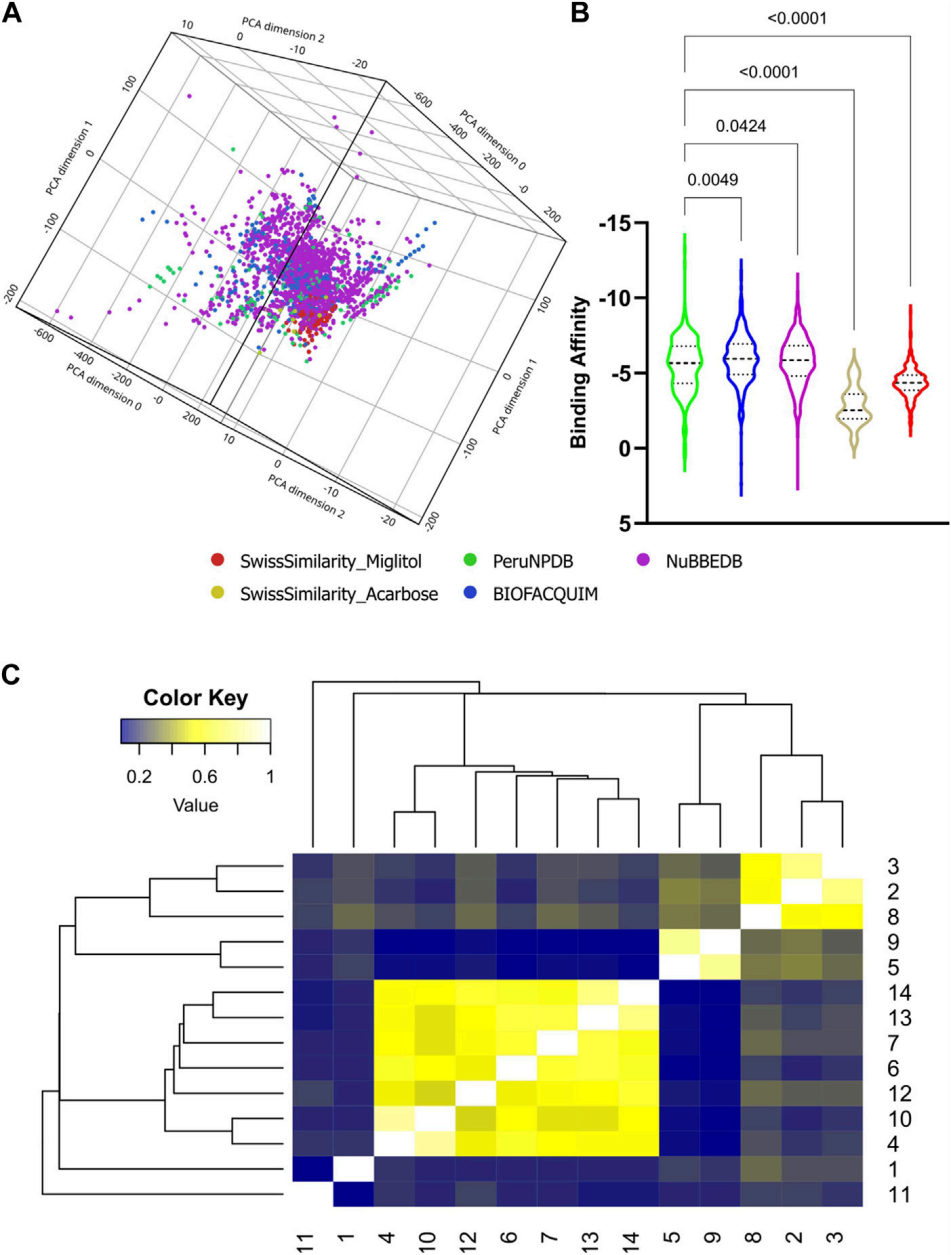
Molecular properties analysis and virtual screening. The chemical space of the generated dataset is represented visually by 3D-PCA **(A)**. binding affinities of NPs against Mannosyl-Oligosaccharide Glucosidase protein **(B)** from the dataset; and heatmap generated with Tanimoto scoring matrix of similar structures among compounds **(C)**.

First, the SMILES from NPs were extracted from three databases: PeruNPDB, BIOFACQUIM, and NuBBE_DB_. These yielded 280, 395, and 2,681 molecules in total. Utilizing the SwissSimilarity site, 3356 molecules of Miglitol and Acarbose’s structural analogs were found, respectively. A total of 3,487 unique molecules were found, and the duplicate compounds were removed (Supplementary Table S1). Figure 2A depicts a visual representation of the chemical space of the data set with 5383 molecules. The PCA analysis simplifies complex chemical features into three key dimensions. The distribution of the molecules in the plot can indicate the chemical diversity of the generated dataset. A widely scattered plot suggests a more chemically diverse dataset, whereas a plot where most compounds cluster together suggests a less diverse set and might share similar chemical features or potentially similar biological activity.

**FIGURE 3:**
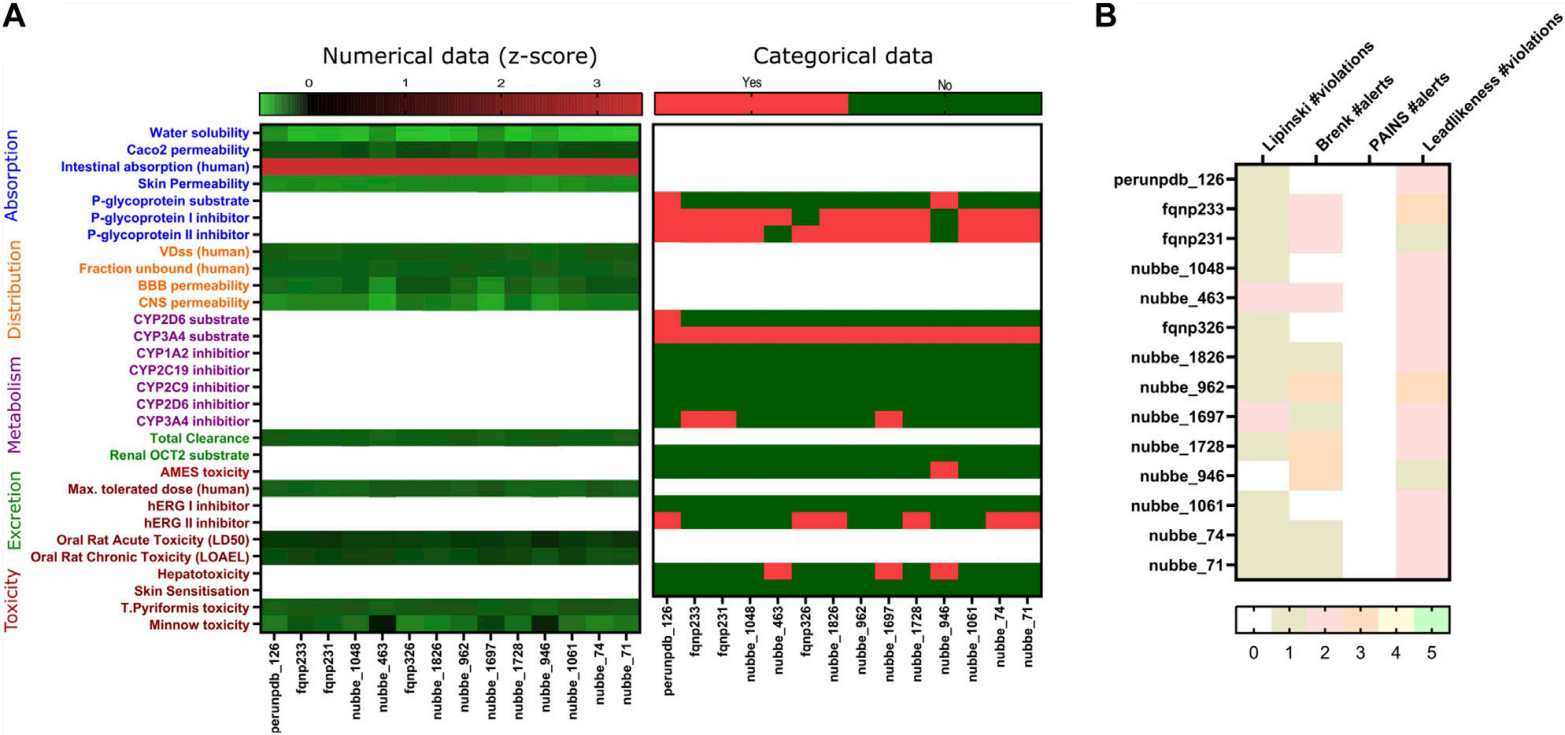
Absorption, Distribution, Metabolism, Excretion (ADMET) prediction heat map of natural products **(A)**; and Lipinski rule of five and PAINS, Brenk alerts, Lead likeness **(B)**.

Subsequently, a virtual screening analysis was conducted on the 3,487 molecules dataset against MOGS (Figure 2B). Compounds demonstrating a binding affinity ≤ −10.00 kcal/mol were considered as potential leads for further investigation. This threshold was selected based on the binding affinities of known inhibitors, Miglitol (−6.577 kcal/mol) and Acarbose (−7.551 kcal/mol), which functioned as controls. Hence, Tanimoto clustering was used as an efficient tool to screen this set for structural similarity by employing molecular fingerprints that encode relevant structural features and quantify their similarity pairwise. Hierarchical clustering, using the Tanimoto distance (0–1), reveals patterns in the dataset. Clusters containing molecules with high Tanimoto similarity highlight candidates with potentially shared bioactivity (Figure 2C), and observing the ADMET dataset analysis with favorable pharmacokinetic properties, indicating potential bioavailability and reduced systemic toxicity (Figure 3A), three NPs were selected: Subsessiline (PubChem CID: 182033) extracted from *Abuta rufescens* (Swaffar et al., 2012), Ocotillone (PubChem CID: 12313665) extracted from *Cabralea canjerana* (Sarria et al., 2011)*,* and Humilinolide G (PubChem CID: 163100483) extracted from *Swietenia humilis* (Ovalle-Magallanes et al., 2015). Also, the analysis confirmed that adherence to Lipinski’s rule of five (molecular weight <500, logP <5, more than 5 hydrogen donors and more than 10 hydrogen bond acceptors) increases the likelihood of good oral bioavailability and membrane permeability. The analysis confirmed adherence to Lipinski’s rule of five (molecular weight <500, a logP value <5, no more than 5 hydrogen bond donors, and no more than 10 hydrogen bond acceptors), which generally increases the likelihood of good oral bioavailability and membrane permeability, absence of PAINS which often act as nonspecific binders in assays, leading to false positives (Dahlin et al., 2015), Brenk alerts avoiding structural motifs flagged helping to minimize potential metabolic liabilities and chemical reactivity issues (Brenk et al., 2008); and Lead likeness characteristics promoting smaller and less complex molecule designs (Teague et al., 1999), suggesting favorable drug-like properties (Figure 3B).

### 3.2 Molecular dynamics simulation of MOGS

**FIGURE 4:**
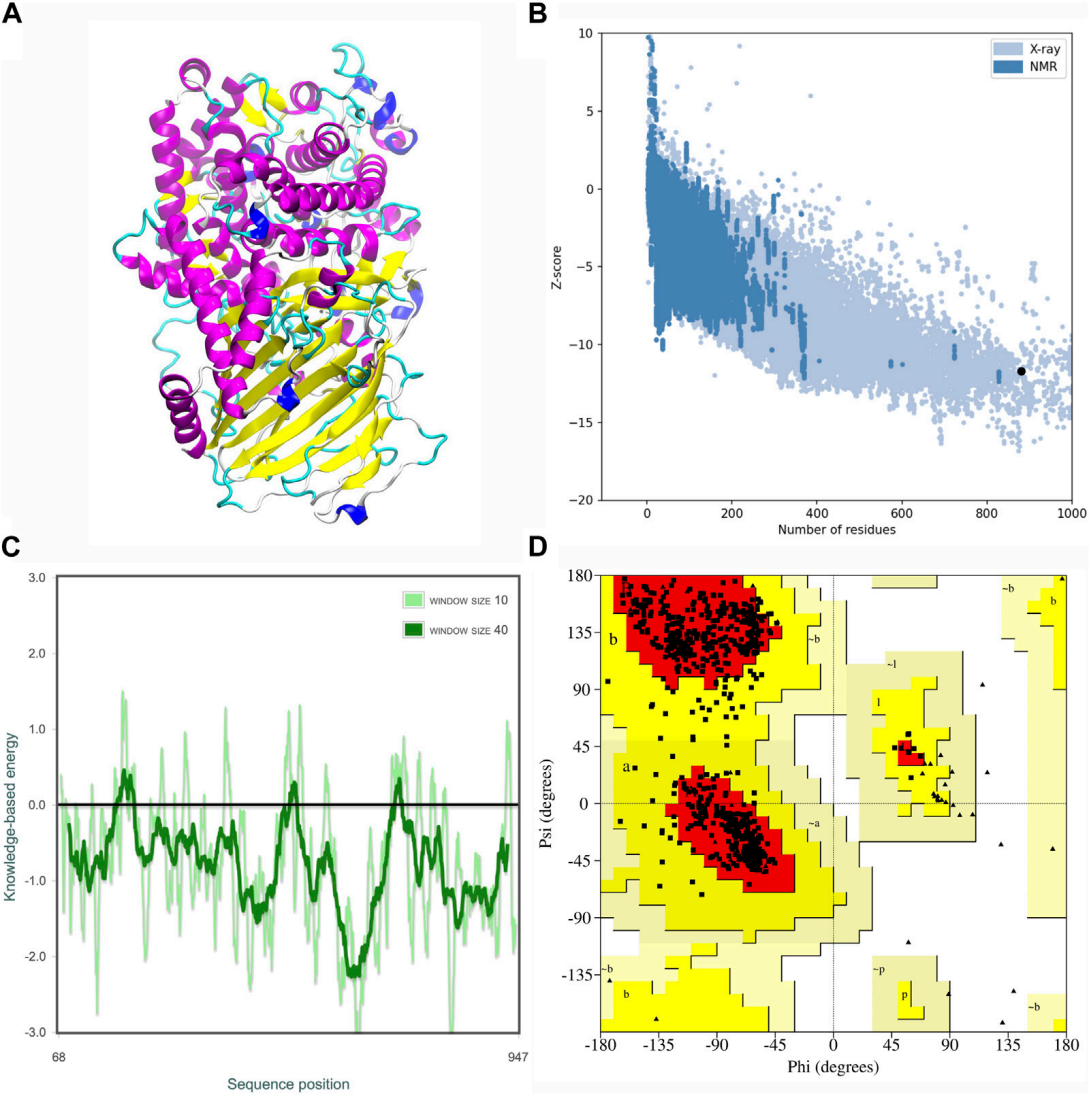
Tertiary structural anticipation and assessment of the MOGS protein. The predicted three-dimensional structure **(A)**, The overall model quality by Z-score plot (−11.00) **(B)**, The local model quality assessment **(C)**, and Ramachandran plot: 91.9% residues in favored region, 8.1% in additionally allowed region, and no residues were found in disallowed conformations **(D)**.

The modeled MOGS protein structure was analyzed and validated (Figure 4A). The Ramachandran plot analysis, a tool for assessing protein structure quality, indicated a well-folded protein. The majority of residues (91.9%) were found in the most favorable structural conformations, residues in additional allowed regions (8.17%), number of non-glycine and non-proline residues (778, 100%), and none in generous conformations in disallowed regions. No residues were found in disallowed conformations (Figure 4C). Additionally, a Z-score of −11.00 (Figure 4B) and energy plots gave reasonable energy values for the structure (Figure 4D), supporting the model’s overall quality.

**FIGURE 5:**
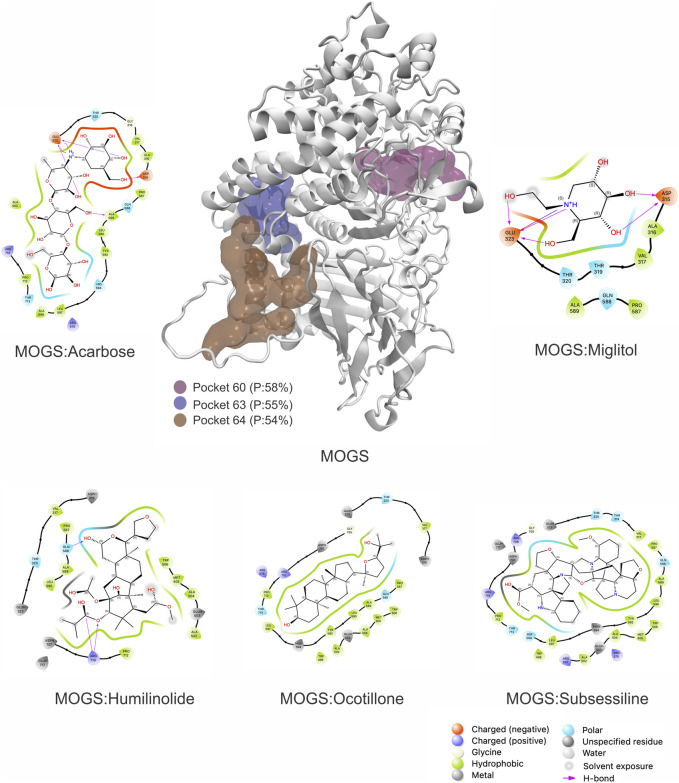
3D structure of Mannosyl-oligossacharide glucosidase (MOGS) Highlighting Druggable Sites and Docking Interactions against controls and the top three natural products found: Ocotillone (NuBBEDB), Huminolide G (BIOFACQUIM), and Subsessiline (PeruNPDB). P = Druggable probability.

Building upon this validation, the MOGS protein model was analyzed using the PASSer software. This analysis identified 64 potential druggable regions. The top three druggable pockets were ranked as numbers 60, 63, and 64, with druggable probabilities of 58%, 55%, and 54%, respectively (Figure 5). The target chosen was pocket number 60 (Grid settings: x = −6, y = 17, and z = 69; Total size: x = 20, y = 20, and z = 20) due to the following properties: The druggability score of 0.666, indicates a moderate potential for binding drug-like molecules. Scores closer to 1 suggest higher druggability. The large volume of 1667.223 suggests the pocket can accommodate reasonably sized drug molecules. The high hydrophobicity score of 27.125 indicates a strong preference for non-polar molecules or sections of molecules. The moderate proportion of polar atoms (39.796%) suggests some potential for interaction with polar parts of drug molecules (like hydrogen bond donors or acceptors), and the flexibility score of 0.844 indicates some adaptability. This could allow the pocket slightly adjust its conformation to fit a drug molecule. The total number of pockets and properties is shown in Supplementary Table S2.

The thermodynamic parameters were calculated and computed with a 100 ns NPT simulation snapshot. Analysis of the backbone involved assessing the root mean squared deviation (RMSD) and root mean squared fluctuations (RMSF) per residue. Furthermore, the radius of gyration (RG) was assessed, quantifying the mass distribution dispersion concerning the central axis. The compactness of a protein, crucial for its folding rate, was directly linked to RG, especially when computed using advanced computational methods (Lobanov et al., 2008). To ascertain the accessible surface area of solvent molecules on the protein, the solvent-accessible surface area (SASA) was analyzed. Conformational changes occur in proteins in response to external stresses, such as the binding of a foreign substance like a medication; these changes increase the solubility of hydrophobic residues.

**FIGURE 6:**
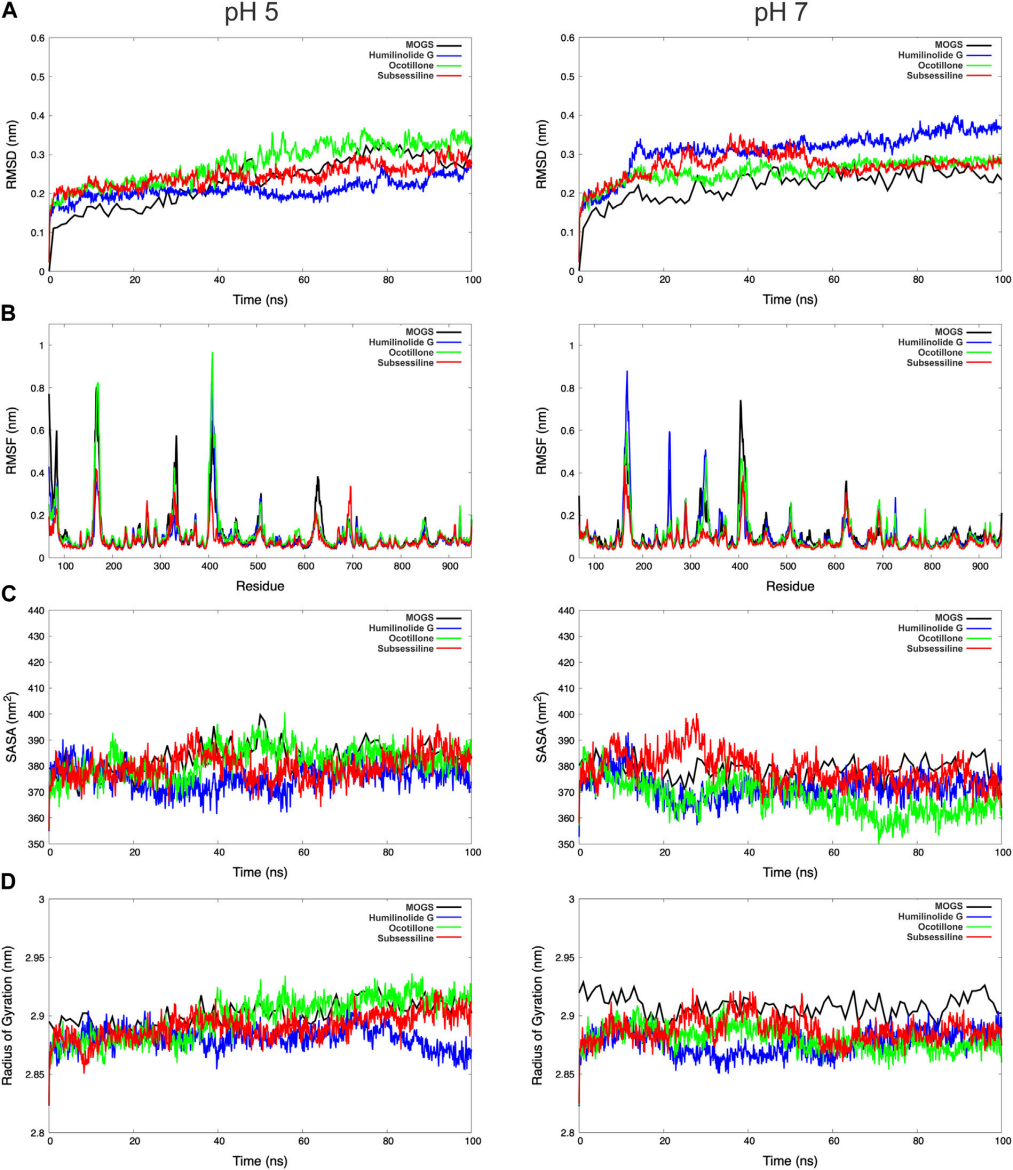
Representation of DM trajectories over 100 ns for Mannosyl-Oligosaccharide Glucosidase, Humilinolide G, Ocotillone and Subsessiline. The figures on the left and right of the panel above were performed at pH 5 and pH 7, respectively. Root mean squared deviation (RMSD) **(A)**; root mean squared fluctuations (RMSF) **(B)**; solvent-accessible surface area (SASA) **(C)**; and radius of gyration (RG) **(D)**.

In general, the average RG and SASA values of the study indicated that proteins linked to pharmaceutical substances had small conformational changes, most likely as a result of ligands occupying their active sites (Durham et al., 2009). The tetrameric conformation of the modeled MOGS exhibited a stable behavior throughout the 100 ns snapshot of MDS conducted at pH 7. It showed a more stable behavior than 5, as shown in Figure 6. The RMSD values of the systems performed to verify the similarity between a protein-bound and not-bound ligand for MOGS receptor were as follows: MOGS (pH 5: 0.23 nm and pH 7: 0.22 nm), MOGS:Humilinolide G (pH 5: 0.21 nm and pH 7: 0.31 nm), MOGS:Ocotillone acid (pH 5: 0.28 nm and pH 7: 0.26 nm), and MOGS:Subsessiline (pH 5: 0.25 nm and pH 7: 0.27 nm). On the other hand, the MOGS: Ocotillone system exhibits low fluctuations in loop peaks as measured by the RMSF per residue, with low peaks at pH 5 at 0.1065, 0.1213, and 0.5732 nm. Meanwhile, the MOGS: Subsessiline system shows low peaks at pH 7 at 0.0528, 0.0564, 0.0689, 0.0694, 0.0734, 0.1197, 0.1380, and 0.2151 nm. The greater RG values obtained from Ocotillone at pH 5 (2.90 nm) and Subsessiline at pH 7 (2.89 nm) showed that the MOGS structure was less compact. In the same way, the average SASA value of Ocotillone at pH 5 (381.80 nm^2^) and Subsessiline at pH 7 (378.86 nm^2^) was higher in comparison to the remaining compounds, indicating lower compactation of MOGS.

### 3.3 Molecular Mechanics/Poisson-Boltzmann (Generalized Born) Surface Area (MM/PB(GB)SA)

The estimation of the binding free energy (ΔG) is made possible by the utilization of continuum solvation implicit models in conjunction with Molecular Mechanics Poisson–Boltzmann Surface Area (MM/PBSA) and Molecular Mechanics Generalized Born Surface Area (MM/GBSA) methods. Through the examination of multiple conformations retrieved from the last 100 frames of the MD simulations, the ΔG values were determined in which the most suitable results were the systems MOGS:Ocotillone at pH 5 (−12.96 kcal·mol^−1^) and MOGS:Subsessiline at pH 7 (−18.67 kcal·mol^−1^) suggesting them as the most suitable candidates among the three chosen initially. The estimated phase-gas binding free energy (ΔGgas) also provided the highest energy contribution for this system, with values of −47.62 and −32.98 kcal·mol^−1^, respectively.

**FIGURE 7:**
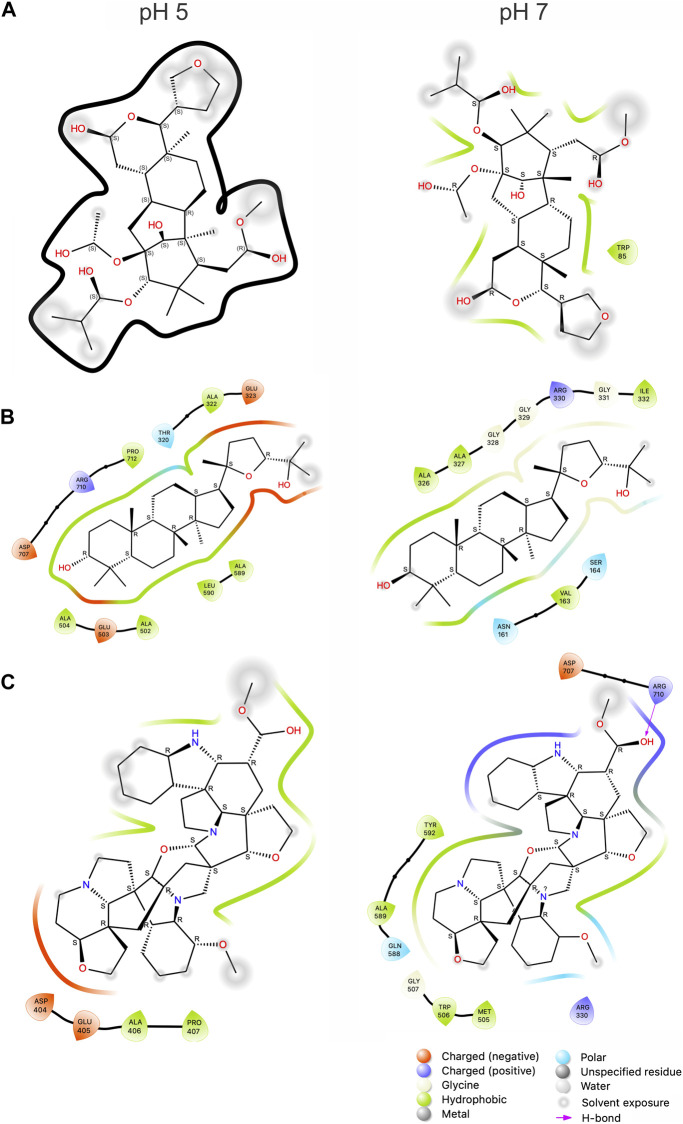
2D representation of the top inhibitors with Mannosyl-Oligosaccharide Glucosidase and compounds computed from 100 ns MD simulation. Humilinolide G **(A)**, Ocotillone **(B)**, and Subsessiline **(C)**. The bottom legends identify the types of interaction and bonds with their corresponding color codes. The thick black line in section A indicates no binding between NP and MOGS.

The data shown in Table 1 emphasizes the significant effect of van der Waals energies alongside electrostatic and generalized born energies. The analysis at two different pH levels can positively or negatively influence the protein/ligand binding due to its effect on hydrophobic interactions. In this context, the electrostatic energies (ΔEele) of Subsessiline, among others, contributed more positively to this binding at a pH of 7 than a pH of 5. The aforementioned pattern was similarly noted in the measurement of solvation energies (ΔGsolv), where negative values indicate an unfavorable contribution to the protein-ligand interaction. Based on the 2D interaction summary represented in Figure 7, it is evident that, at pH 7, the ligand Subsseline effectively replicated a critical hydrogen bonding interaction with specific amino acid residue ARG710.

**TABLE 1:** Average value of the binding free energy determined by the MM/PBSA method.

Energy	Subsessiline				Humilidoline G				Ocotillone			
	pH 5		pH 7		pH 5		pH 7		pH 5		pH7	
	Average	DesV	Average	DesV	Average	DesV	Average	DesV	Average	DesV	Average	DesV
**ΔVDWAALS**	−6.37	0.02	−36.20	1.43	−13.81	2.28	−5.10	2.55	−29.02	2.46	−18.34	0.97
**ΔEEL**	440.10	95.71	−11.42	9.57	−3.54	0.10	−2.40	0.82	−3.96	4.79	−1.91	3.94
**ΔEGB**	−434.87	96.90	33.48	15.08	11.66	2.92	6.16	1.83	23.75	0.21	14.32	3.47
**ΔESURF**	−0.79	0.08	−4.53	0.26	−2.00	0.00	−0.72	0.03	−3.73	0.11	−2.41	0.37
**ΔGGAS**	433.73	95.72	−47.62	9.78	−17.35	2.85	−7.50	3.14	−32.98	5.53	−20.25	4.26
**ΔGSOLV**	−435.66	96.90	28.95	15.08	9.66	2.92	5.44	1.83	20.02	0.24	11.91	3.49
**ΔTOTAL**	−1.92	136.20	−18.67	17.97	−7.70	4.08	−2.05	3.63	−12.96	5.54	−8.34	5.51

ΔVDWAALS, Van DerWaals energy; ΔEel, electrostatic interactions energy; ΔEgb, electrostatic contribution free energy calculated by generalized Born (polar contribution to solvation energy); ΔEsurf, nonpolar component of the solvation energy; ΔGgas, estimated phase-gas binding free energy; ΔGsolv, estimates binding free energy solvent (total solvation energy); ΔGTOTAL, estimated binding free energy. Values of energy in kcal.mol^−1^.

## 4 Discussion

VL disproportionately impacts economically disadvantaged communities in tropical and subtropical areas, with an estimated 500,000 new cases annually (Alvar et al., 2012; Africa and Asia, 2018). Sudan, Ethiopia, Brazil, Kenya, Somalia, and South Sudan represent most cases (Hotez et al., 2020). If acute and untreated, the death rate associated with VL exceeds 95% (Rodrigues Monteiro et al., 2023). Treatment options are hampered by difficulties, such as toxicity, prolonged administration, and the emergence of drug resistance; furthermore, the unavailability of a vaccine hinders prevention efforts. The urgent necessity for improved diagnostics, vector control strategies, novel and safer medications, an effective vaccine, and an emphasis on addressing the social and economic disparities that sustain the transmission of this neglected tropical disease is highlighted by the challenges raised by VL (De Rycker et al., 2018).

NPs, which have enhanced bioactive potential and structural diversity, are a potentially promising source of possible anti-leishmanial compounds. Implementing CADD techniques to repurpose NPs may accelerate drug discovery and improve affordability. CADD makes virtual screening against *Leishmania* targets possible, enhancing potential NP scaffolds (Barazorda-Ccahuana et al., 2023a; Luna et al., 2023; Barazorda-Ccahuana et al., 2024). Examples of natural compounds that have shown anti-leishmanial activity comprise alkaloids (like berberine and piperine) (Sakyi et al., 2021), flavonoids (such as quercetin and rutin) (Mehwish et al., 2021), and terpenes (like limonene and eugenol) (Almeida-Bezerra et al., 2022). Despite the ongoing difficulties associated with compound isolation and validation, integrating NPs research and CADD, including chemoinformatics (Medina-Franco and Saldívar-González, 2020), emerges as a persuasive approach to address the increasing demand for novel and efficacious treatments for VL.

Due to the promising results obtained in our previous research (Chavez-Fumagalli et al., 2019; Costa et al., 2021), we utilized, modeled, and validated the MOGS protein to identify potentially druggable pockets. This approach is crucial in drug design, allowing us to target protein activity for therapeutic purposes. Analysis of the modeled MOGS structure revealed several promising pockets, with pocket number 60 showing particularly favorable characteristics (Figure 5). Our work confirmed this pocket’s ability to bind to NPs, highlighting its potential to modulate the glucosidase’s biological activity.

Consequently, the current study aimed to use CADD approaches to identify NPs from the NuBBEDB, BIOFACQUIM, and PeruNPDB databases, as well as Miglitol and Acarbose analogs that may have antileishmanial and anti-MOGS properties. At the outset, we distinguished three compounds, namely, Ocotillone (PubChem CID 12313665), Subsessiline (PubChem CID 182033), and Humilinolide G (PubChem CID 163100483), which stabilized the MOGS structure with an average RMSD higher than that of the protein without compounds. The relevance of certain N-glycosylation processes on the biology of *Leishmania* highlights the promise of MOGS as a prospective target for anti-leishmanial drugs (Naderer et al., 2004). Peptide-specific inhibitors, such as those evaluated here, can potentially interfere with critical N-glycan production pathways. A lack of homology between *Leishmania* MOGS and its human counterpart can significantly enhance the drug’s specificity, thereby reducing toxicity toward the host (Chavez-Fumagalli et al., 2019). Although these NPs have significant potential for drug development, their effectiveness within macrophages and MOGS can be significantly affected by the environment. An effective method for analyzing this effectiveness was using molecular docking and dynamic simulations. These techniques can extensively consider crucial factors such as pH levels, as they possess the potential to drastically modify the charge distribution, conformation, and binding characteristics of the NPs.

It is important to note that *Leishmania* has a unique lifecycle encompassing two stages. The promastigote stage thrives within its sandfly host, facing conditions ranging from slightly acidic to alkaline. Upon entering a mammalian host, it transforms into the amastigote stage, adapted to survive within the acidic environment of the phagolysosomes of macrophages (Rosenzweig et al., 2008). Understanding insight into the processes by which *Leishmania* recognizes, adapts to, and utilizes these significant fluctuations in pH is critical to unraveling its interesting life cycle and discovering prospective treatment targets. Although several organisms would perish in the acidic environment of the phagolysosome of a host cell, *Leishmania* has developed ways that allow it to survive and develop in this situation. An examination of the impacts of pH reveals that the parasite possesses a resistance mechanism to acid levels, responding through stress-response pathways and specific transporters to maintain intracellular balance (McConville and Naderer, 2011). Moreover, *Leishmania* prevents the usual phagosome maturation process, ensuring a safe environment within the host cell and avoiding exposure to the lysosomal damaging enzymes and highest acidity (Podinovskaia and Descoteaux, 2015).

Significantly, *Leishmania* utilizes the acidic environment (pH 4.5–5.5) of phagolysosomes as an important trigger for its development. Research findings indicate that the parasite may have pH-sensing systems that initiate its transformation from external promastigotes to intracellular amastigotes (Pal et al., 2017). Significant modifications in gene expression occur during this transition, leading to the synthesis of surface chemicals and proteins optimized for the survival of amastigote cells (Cohen-Freue et al., 2007). Likewise, the function of MOGS, in this pH-dependent transition, is an exciting topic for further studies. Consequently, considering this information, we performed a molecular dynamic simulation at two different pH levels, 5 and 7, to evaluate the effects of this parameter (Figure 6).

**FIGURE 8:**
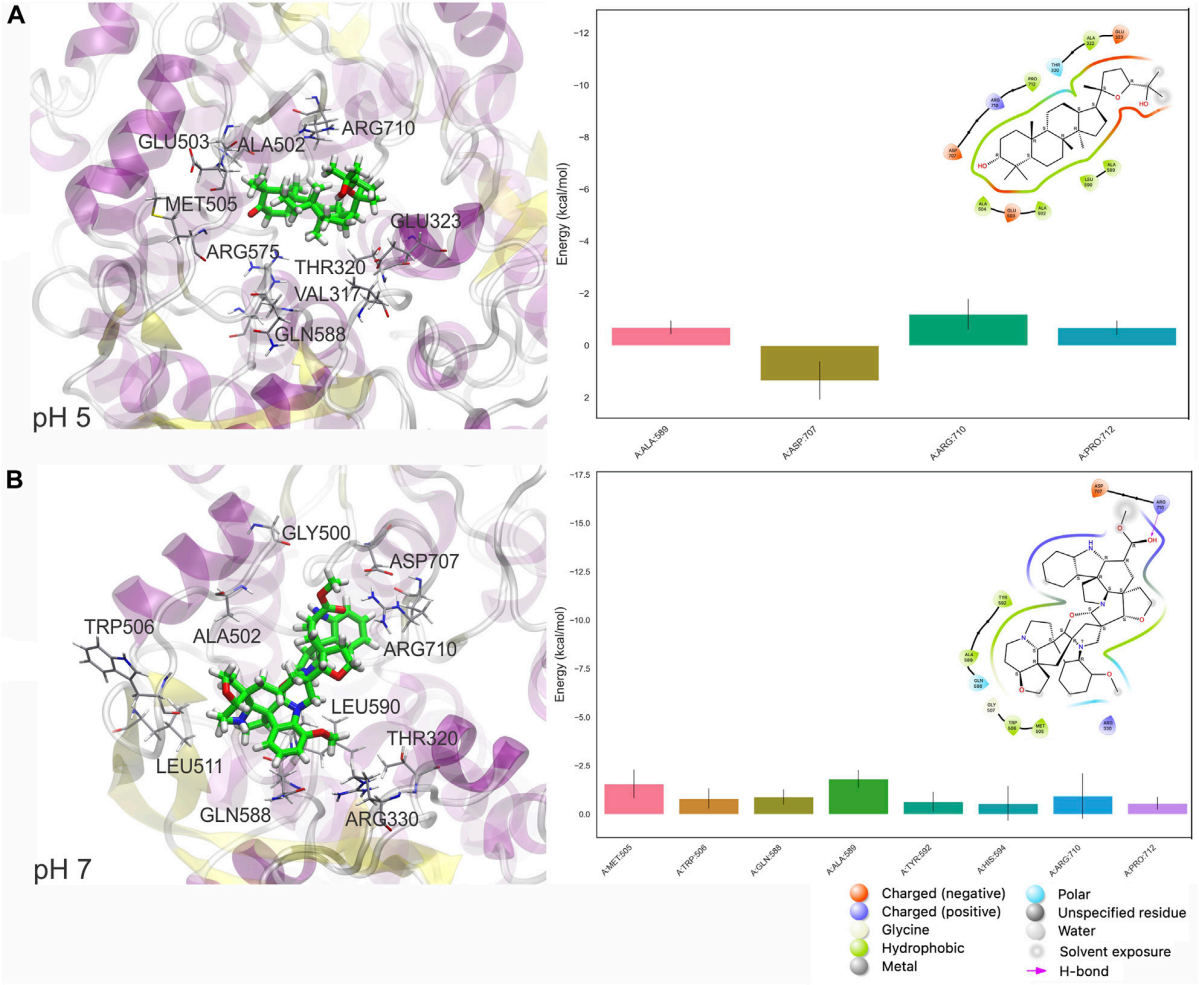
Pictorial 3D (Left) and 2D (right) representation of the best binding free energy between Ocotillone **(A)** and Subsessiline **(B)** at pH 5 and 7, respectively; and Mannosyl-Oligosaccharide Glucosidase (MOGS), computed from the last 10 ns (100 frames) of 100 ns MD simulation.

At pH 5, also shown by the values in Table 2 significant conformational changes in the protein were found in interaction with Ocotillone, as indicated by an RMSD value of 0.28 ± 0.05 nm, which was higher than the value of the protein in the absence of ligands. Subsessiline subsequently followed with an RMSD of 0.25 ± 0.03 nm. Humidilidoline G, in comparison to the aforementioned, produced the most stable protein structure, as indicated by its average RMSD value of 0.21 ± 0.02. This is because, in this system, the active center does not retain the compound for an extended time at pH 5. Concerning the solvent-accessible surface area (SASA) and radius of gyration (RG), Ocotillone and Subsessiline have more effective results than Humilidoline G. The stability of the compound related to MOGS at pH 5 was Ocotillone > Subsessiline > Humidilidoline G. Additionally, our research revealed the catalytic dyad for MOGS, a key component of the protein binding site, composed of hydrophobic bonds with Ocotillone ALA 589 (0.1213 nm), ARG 710 (0.1065 nm), and PRO 172 (0.5732 nm) (Figure 8A).

**TABLE 2:** RMSD, SASA, and RG, average values of the top three targets: Humilidoline G, Ocotillone and Subsessiline, based on 100 ns of MD simulations.

Structure	pH 5						pH 7					
	RMSD (nm)		SASA (nm^2^)		RG (nm)		RMSD (nm)		SASA (nm^2^)		RG (nm)	
	Average	DesV	Average	DesV	Average	DesV	Average	DesV	Average	DesV	Average	DesV
**MOGS**	0.23	0.07	382.84	5.57	2.90	0.01	0.22	0.04	378.32	4.05	2.91	0.01
**Humilidoline G**	0.21	0.02	375.63	4.96	2.88	0.01	0.31	0.05	371.76	4.95	2.88	0.01
**Ocotillone**	0.28	0.05	381.80	5.70	2.90	0.02	0.26	0.03	367.60	6.64	2.88	0.01
**Subsessiline**	0.25	0.03	379.83	5.32	2.89	0.01	0.27	0.03	378.86	6.39	2.89	0.01

At pH 7, the compounds’ behavior increases the therapeutic target’s conformational changes. Humilidoline G and Ocotillone exhibited short retention at the active center within these systems (10 ns of 100 ns). Meanwhile, Subsessiline was the compound that remained in its position in the active site after MD concluded. Nevertheless, the proteins binding to ligands exhibited more significant oscillations in the systems’ RMSD values than those without ligands. In systems involving Humilidoline G and Ocotillone, the solvent has reduced access to the protein surface, as indicated by the SASA value. The stability of the compound related to MOGS at pH 7. was Subsessiline > Humidilidoline G > Ocotillone. The catalytic dyad for MOGS (Figure 8B), composed of hydrophobic bonds with Subsessiline MET 505 (0.1197 nm), TRP 506 (0.1380 nm), GLN 588 (0.0689 nm) ALA 589 (0.0694 nm) TYR 592 (0.0564 nm) HIS 594 (0.0528 nm) and PRO 172 (0.2151 nm), and hydrogen bond ARG 710 (0.0734 nm) (Figure 7C). The specificity and directionality of hydrogen bonds enhance drug selectivity, reducing off-target effects. These bonds also stabilize the drug-target complex, potentially influencing a drug’s residence time and improving its overall efficacy. Unfortunately, we could not find literature discussing the role of specific amino acid residues in this protein’s conformation, highlighting its novelty and the need for further research.

Ocotillone and Subsessiline might inhibit protein folding in *Leishmania*’s transition from promastigote to amastigote stage, offering a potential treatment for Leishmaniasis. Focusing on this critical phase, these NPs could limit the parasite’s capacity to settle within the macrophage, preventing its survival and disease development. Moreover, these NPs demonstrate stable performance across different pH levels in the macrophage’s phagolysosome, indicating their efficacy throughout the infection process. This stability is crucial because the parasite effectively takes advantage of these pH changes to control the phagolysosome environment in its favor. Furthermore, evaluating ADMET (absorption, distribution, metabolism, excretion, and toxicity) characteristics and adherence to Lipinski’s Rule of Five may predict low toxicity linked to these natural molecules. These assays provide an important initial evaluation of a drug candidate’s drug-likeness and potential for absorption through the digestive system, including parameters such as molecular size, lipophilicity, and hydrogen bonding potential.

Ocotillone, a naturally occurring compound isolated from *C. canjerana*, a native tree in Brazil, has a promising potential for a wide range of biological applications. It shows significant antifeedant efficacy against *Spodoptera litura* in insect bioassays, as evidenced by a Percentage Feeding Index (PFI) that is comparable to that of well-established biopesticides such as Limonin, Azadiradione, and Epoxyazadiradione (Sarria et al., 2011). Furthermore, Ocotillone shows considerable *in vitro* anticancer activity when tested against cancer cell lines MCF-7 (breast), NCI-H460 (lung), and A375-C5 (melanoma). Also, it synergizes with colchicine, combating multidrug resistance (MDR) in cancer treatment (M Cazal et al., 2010). Moreover, dichloromethane extracts derived from closely related species (e.g., *P. grandiflorus*) containing Ocotillone in its composition exhibit a significant inhibitory effect against the fungus *Leucoagaricus gongylophorus* (de Souza et al., 2005).

Subsessiline, on the other hand, was initially isolated from the native tree of the Peruvian Amazon, *A. rufescens* (Swaffar et al., 2012), and demonstrated *in vitro* efficacy against *Plasmodium falciparum*. Although the precise efficacy of the substance was not disclosed, it exhibited an IC50 ranging from 4 to 40 μg per milliliter, inhibiting the *in vitro* proliferation of parasites. Notably, this extract also inhibited the parasite’s capacity to transform hazardous heme into innocuous hemozoin crystals, indicating the possibility of a second mechanism of action (Ruiz et al., 2011). Roumy et al. (2007), also investigated this effect and evaluated the antiplasmodial activity of *A. rufescens*, which is historically utilized by the Shipibo-Conibo community in Peru. The activity of the leaves and bark extracts against *P. falciparum* was found to be good to moderate, as indicated by IC50 values ranging from 2.3 to 7.9 g/mL. This discovery is consistent with antiplasmodial alkaloids previously documented in the stem (such as isoquinoline, azafluoranthene, and oxoaporphine), supporting the traditional use of *A. rufescens* as a therapy for malaria.

Examining pH dynamics in *Leishmania* makes a significant contribution that reaches beyond basic comprehension of the parasite’s biology and pathology. It provides valuable insights into potential treatment pathways, making room for novel strategies to overcome a disease affecting millions of people worldwide. Researchers perform phagosome infection models and *in vitro* pH manipulations with great attention to detail to analyze pH sensing, adaptability, and differentiation mechanisms. Critical molecular targets, such as MOGS, for developing novel drugs could be proposed by revealing these systems. Furthermore, it is significant to understand the impact of pH on the absorption, stability, and efficacy of drugs to develop highly effective anti-leishmanial substances. Notably, therapeutic approaches could potentially achieve a synergistic effect by concurrently targeting *Leishmania*’s pH regulating systems and inhibitors of differentiation processes, including those dependent on MOGS-dependent N-glycosylation. To the best of our knowledge, no studies have been published on the pharmacological potential activity of Ocotillone and Subsessiline against *Leishmania*. While considering the pH variations, the MOGS as a target and comparing and validating this catalytic dyad is still challenging.

## 5 Conclusion

This study aimed to identify *L. infantum* inhibitors from NP scaffolds through *in silico* analysis and database mining. Based on similarity analysis of NPs and existing data in three major public NP databases (NuBBE_DB_, BIOFACQUIM, and PERUNPDB). Ocotillone (PubChem CID: 12313665) and Subsessiline (PubChem CID: 182033) were evaluated at pH 5 and 7, respectively, and emerged as the most promising candidates. *In silico* docking analyses indicated a favorable affinity for the *L. infantum* MOGS enzyme that could suggest antileishmanial activity. It is worth mentioning that the compounds showed no predicted toxicity and were shown *in silico* to be compatible with oral administration. Additional *in vitro* and *in vivo* experiments are necessary to validate the possible therapeutic potential of Ocotillone and Subsessiline in the treatment against VL, as suggested by the results described here.

## Data Availability

The original contributions presented in the study are included in the article/Supplementary Material, further inquiries can be directed to the corresponding author.
