# Supplementary material for: Targeting Leishmania infantum Mannosyl-oligosaccharide glucosidase with natural products: potential pH-dependent inhibition explored through computer-aided drug design
Source: Front Pharmacol. 2024 May 30;15:1403203. doi: 10.3389/fphar.2024.1403203 (PMC11169604; doi:10.3389/fphar.2024.1403203)
Supplement: Supplementary file 1 [file DataSheet1.PDF]

Supplementary Material

Targeting *Leishmania infantum* Mannosyl-oligosaccharide glucosidase with natural products: pH-dependent inhibition explored through computer-aided drug design.

Luis Daniel Goyzueta-Mamani, Haruna Luz Barazorda-Ccahuana, Mayron Antonio Candia-Puma, Alexsandro Sobreira Galdino, Ricardo Andrez Machado-de-Avila, Rodolfo Cordeiro Giunchetti, José L. Medina-Franco, Mónica Florin-Christensen, Eduardo Antonio Ferraz Coelho, Miguel Angel Chávez-Fumagalli*

*** Correspondence:** Corresponding Author: mchavezf@ucsm.edu.pe

Table S1. Docking results for NPs ligands from BIOFACQUIM, PERUNPDB, and NuBBEDB with AutoDock 4.0.

Compound	Binding energy (kcal/mol)
perunpdb126	-12.2
fqnp233	-11.76
fqnp231	-11.3
nubbe1048	-11.1
nubbe463	-10.88
zinc000095551509	-10.85
fqnp326	-10.65
nubbe1826	-10.44
nubbe962	-10.44
nubbe1697	-10.4
zinc000100008319	-10.37
nubbe1728	-10.35
zinc000019632706	-10.34
nubbe946	-10.22
nubbe1061	-10.09
nubbe74	-10.09
nubbe71	-10.02
zinc000001319780	-10.02
fqnp48	-9.97
zinc000150338708	-9.95
nubbe1698	-9.86
zinc000003812988	-9.82
nubbe428	-9.8
fqnp271	-9.77
nubbe956	-9.74
nubbe429	-9.72
fqnp327	-9.66
fqnp49	-9.64
nubbe1051	-9.6
nubbe2281	-9.6
zinc000053683271	-9.6
fqnp269	-9.55
nubbe1062	-9.48
nubbe364uff	-9.48
nubbe1347	-9.42
nubbe1706	-9.42
zinc000100037890	-9.42
nubbe1348	-9.41
nubbe589uff	-9.4
perunpdb268	-9.39
nubbe2503	-9.38
nubbe366uff	-9.36

nubbe1523	-9.34
nubbe438	-9.34
nubbe133	-9.33
nubbe2301	-9.33
nubbe72	-9.29
nubbe72uff	-9.29
nubbe71uff	-9.28
fqnp281	-9.27
nubbe1033	-9.26
nubbe1063	-9.26
nubbe1430	-9.26
nubbe190uff	-9.25
zinc000004097383	-9.25
nubbe21	-9.23
nubbe1370	-9.2
nubbe364	-9.19
nubbe347	-9.17
nubbe415	-9.17
fqnp325	-9.15
zinc70673654	-9.15
nubbe1271	-9.14
zinc000001280665	-9.14
nubbe2161	-9.13
nubbe415uff	-9.13
nubbe78	-9.12
nubbe1365	-9.11
zinc000003861599	-9.11
nubbe585uff	-9.1
nubbe1064	-9.07
nubbe1130	-9.07
nubbe263uff	-9.07
fqnp153	-9.06
zinc000012503291	-9.05
nubbe2303	-9.01
zinc000410428674	-9.01
nubbe2491	-9
zinc68590493	-9
fqnp152	-8.99
nubbe1005	-8.97
nubbe87uff	-8.96
nubbe438uff	-8.94
nubbe1042	-8.93
nubbe1038	-8.92

nubbe1257	-8.91
nubbe589	-8.91
nubbe1144	-8.89
nubbe2378	-8.86
nubbe1928	-8.85
nubbe1204	-8.84
nubbe256uff	-8.84
nubbe1355	-8.83
nubbe1891	-8.83
nubbe228	-8.83
perunpdb147	-8.79
zinc000003927200	-8.79
nubbe607uff	-8.78
nubbe2264	-8.77
zinc000100037885	-8.77
nubbe1455	-8.74
nubbe621	-8.74
zinc000003813047	-8.74
zinc000013831141	-8.74
zinc000256433948	-8.74
nubbe191	-8.71
zinc000014768621	-8.7
nubbe1863	-8.69
nubbe1353	-8.68
nubbe439	-8.68
perunpdb22	-8.68
nubbe1416	-8.67
nubbe410	-8.67
perunpdb267	-8.67
nubbe1037	-8.66
nubbe1052	-8.66
nubbe928	-8.66
fqnp106	-8.66
zinc000003973334	-8.66
nubbe386	-8.65
nubbe2426	-8.64
nubbe87	-8.64
nubbe92	-8.64
zinc000052509463	-8.64
nubbe960	-8.63
zinc000002568036	-8.63
nubbe190	-8.61
nubbe959	-8.6

nubbe1146	-8.59
nubbe388	-8.59
fqnp207	-8.59
nubbe1176	-8.58
nubbe1110	-8.56
nubbe2484	-8.56
fqnp235	-8.56
zinc000003915154	-8.56
nubbe1672	-8.55
nubbe416uff	-8.55
nubbe75uff	-8.55
fqnp143	-8.54
nubbe1131	-8.53
nubbe240uff	-8.53
nubbe1431	-8.52
fqnp265	-8.52
fqnp270	-8.52
nubbe458uff	-8.51
nubbe146	-8.5
nubbe21uff	-8.5
nubbe462uff	-8.5
nubbe2006	-8.48
fqnp303	-8.48
zinc000100371951	-8.48
nubbe1438	-8.47
nubbe479	-8.47
nubbe247uff	-8.45
perunpdb129	-8.45
fqnp133	-8.45
zinc000001493878	-8.45
fqnp446	-8.44
zinc000000020253	-8.43
zinc000100370145	-8.43
nubbe1039	-8.42
fqnp307	-8.4
nubbe1034	-8.39
zinc000003813003	-8.39
zinc000043206370	-8.39
fqnp282	-8.38
zinc000013648755	-8.38
nubbe268uff	-8.35
zinc000034089131	-8.35
zinc000043450324	-8.34
nubbe1025	-8.33

perunpdb28	-8.33
nubbe1035	-8.32
nubbe966	-8.31
zinc000256433955	-8.31
nubbe1046	-8.3
nubbe1727	-8.3
fqnp154	-8.3
nubbe1926	-8.29
nubbe2360	-8.29
nubbe2495	-8.29
fqnp268	-8.29
fqnp349	-8.29
nubbe1729	-8.28
nubbe1050	-8.27
nubbe2073	-8.27
nubbe1602	-8.25
nubbe407	-8.25
nubbe1168	-8.24
nubbe177uff	-8.24
nubbe2068	-8.24
nubbe608	-8.24
nubbe1096	-8.23
nubbe314	-8.23
nubbe365uff	-8.23
nubbe89uff	-8.23
perunpdb243	-8.23
fqnp201	-8.23
zinc000003818726	-8.23
zinc000003875483	-8.22
nubbe961	-8.21
nubbe266	-8.19
nubbe75	-8.19
fqnp169	-8.19
zinc000030691797	-8.19
nubbe2354	-8.18
nubbe574	-8.18
nubbe1827	-8.17
zinc000006094354	-8.17
nubbe1707	-8.16
nubbe240	-8.16
nubbe605	-8.16
fqnp287	-8.15
nubbe1356	-8.14
nubbe1360	-8.14

nubbe1318	-8.13
nubbe2171	-8.13
fqnp275	-8.13
fqnp386	-8.13
zinc000003927822	-8.12
zinc000004097305	-8.12
zinc000118912393	-8.12
nubbe1047	-8.11
nubbe1141	-8.11
nubbe1725	-8.11
nubbe1357	-8.1
nubbe1453	-8.09
nubbe416	-8.09
nubbe1095	-8.08
nubbe1317	-8.08
nubbe2077	-8.08
nubbe1232	-8.07
nubbe1652	-8.07
zinc000000402954	-8.07
nubbe191uff	-8.06
nubbe1949	-8.06
nubbe2244	-8.06
zinc000011677837	-8.06
nubbe1550	-8.05
nubbe395	-8.05
perunpdb207	-8.05
nubbe1362	-8.04
nubbe2357	-8.04
nubbe479uff	-8.04
nubbe73	-8.04
nubbe216uff	-8.03
nubbe2326	-8.03
nubbe954	-8.03
nubbe189uff	-8.02
nubbe2033	-8.01
nubbe2280	-8.01
nubbe2432	-8.01
nubbe384	-8.01
nubbe2072	-8
nubbe2286	-8
nubbe455uff	-8
fqnp202	-8
zinc000002008310	-8

zinc000100378061	-8
nubbe2363	-7.99
nubbe322uff	-7.99
nubbe857	-7.99
nubbe958	-7.99
zinc000118912450	-7.99
zinc000100013130	-7.98
nubbe1632	-7.97
nubbe379	-7.97
nubbe552	-7.97
nubbe1653	-7.96
nubbe1321	-7.95
nubbe1407	-7.95
nubbe2234	-7.95
nubbe2395	-7.95
perunpdb232	-7.95
fqnp452	-7.95
zinc000051133897	-7.95
zinc000261527196	-7.95
nubbe315	-7.94
nubbe466	-7.94
fqnp455	-7.94
zinc000003881958	-7.94
nubbe258uff	-7.93
fqnp210	-7.93
zinc000000538312	-7.93
nubbe1139	-7.92
nubbe132	-7.92
nubbe1731	-7.92
nubbe2093	-7.92
perunpdb6	-7.92
fqnp212	-7.92
nubbe1231	-7.91
nubbe2239	-7.91
fqnp142	-7.91
nubbe2295	-7.9
perunpdb163	-7.9
fqnp285	-7.9
zinc000003814395	-7.9
nubbe1433	-7.89
nubbe1786	-7.89
nubbe2344	-7.89
nubbe437	-7.89

zinc000011616882	-7.89
nubbe1349	-7.88
nubbe1609	-7.88
perunpdb231	-7.88
zinc000000968264	-7.88
zinc000003784182	-7.88
nubbe1290	-7.87
nubbe386uff	-7.87
zinc000003795819	-7.87
nubbe1579	-7.86
nubbe1661	-7.86
nubbe469	-7.86
nubbe590uff	-7.86
fqnp236	-7.86
nubbe1026	-7.85
nubbe1410	-7.85
nubbe2196	-7.85
nubbe1553	-7.84
nubbe1925	-7.84
nubbe2191	-7.84
nubbe2197	-7.84
nubbe268	-7.84
nubbe365	-7.84
nubbe379uff	-7.84
nubbe553uff	-7.84
fqnp230	-7.84
nubbe1194	-7.83
nubbe2188	-7.83
nubbe2287	-7.83
nubbe257uff	-7.83
nubbe381	-7.83
perunpdb144	-7.83
zinc000003985982	-7.83
zinc000111460375	-7.83
nubbe1392	-7.82
nubbe2396	-7.82
perunpdb131	-7.82
nubbe1183	-7.81
nubbe1322	-7.81
nubbe1327	-7.81
nubbe329	-7.81
nubbe383uff	-7.81
nubbe985	-7.81
zinc000000002299	-7.81

nubbe1136	-7.8
nubbe1584	-7.8
nubbe366	-7.8
nubbe2285	-7.79
nubbe407uff	-7.79
fqnp286	-7.79
zinc000003938695	-7.79
zinc000019632618	-7.79
zinc000085205451	-7.79
nubbe1511	-7.78
nubbe2400	-7.78
nubbe480uff	-7.78
fqnp246	-7.78
zinc000003860453	-7.78
nubbe1989	-7.77
nubbe678	-7.77
nubbe952	-7.77
zinc000000897251	-7.77
zinc000004097416	-7.77
nubbe132uff	-7.76
nubbe1359	-7.76
nubbe396uff	-7.76
nubbe836	-7.76
perunpdb155	-7.76
fqnp107	-7.76
fqnp118	-7.76
nubbe2233	-7.75
nubbe548uff	-7.75
perunpdb230	-7.75
zinc000000584092	-7.75
zinc000003833821	-7.75
nubbe12uff	-7.74
nubbe224	-7.74
nubbe45uff	-7.74
nubbe1917	-7.73
nubbe2156	-7.73
nubbe2429	-7.73
zinc000013520815	-7.73
zinc000118912517	-7.73
zinc68590491	-7.73
perunpdb99	-7.72
nubbe2333	-7.71
perunpdb128	-7.71
perunpdb156	-7.71

nubbe1145	-7.7
nubbe1148	-7.7
nubbe1181	-7.7
nubbe1292	-7.7
nubbe1313	-7.7
nubbe1530	-7.7
nubbe257	-7.7
nubbe540uff	-7.7
zinc000169621200	-7.7
zinc000253530025	-7.7
nubbe1045	-7.69
nubbe1072	-7.69
nubbe1413	-7.69
nubbe381uff	-7.69
nubbe77	-7.69
fqnp295	-7.69
zinc000000000431	-7.69
nubbe1240	-7.68
nubbe1486	-7.68
nubbe326	-7.68
nubbe495	-7.68
nubbe81uff	-7.68
zinc000000004893	-7.68
zinc000003814422	-7.68
zinc000116473771	-7.68
zinc000306122005	-7.68
zinc68590487	-7.68
nubbe1043	-7.67
nubbe1098	-7.67
nubbe12	-7.67
nubbe380uff	-7.67
nubbe461uff	-7.67
nubbe679	-7.67
fqnp206	-7.67
zinc000000538658	-7.67
zinc000000896634	-7.67
zinc000019632917	-7.67
zinc000054053579	-7.67
nubbe1208	-7.66
nubbe2078	-7.66
nubbe2200	-7.66
fqnp203	-7.66
zinc000000601250	-7.66

nubbe1049	-7.65
nubbe1512	-7.65
nubbe1908	-7.65
nubbe2366	-7.65
fqnp211	-7.65
zinc000000005423	-7.65
zinc000006716957	-7.65
nubbe1203	-7.64
nubbe2494	-7.64
nubbe88uff	-7.64
perunpdb154	-7.64
nubbe255uff	-7.63
perunpdb23	-7.63
zinc000169621219	-7.63
nubbe1583	-7.62
nubbe2241	-7.62
nubbe2507	-7.62
nubbe328	-7.62
nubbe997	-7.62
perunpdb229	-7.62
fqnp276	-7.62
nubbe1354	-7.61
nubbe256	-7.61
nubbe542	-7.61
perunpdb106	-7.61
perunpdb208	-7.61
zinc000004213474	-7.61
zinc000014261579	-7.61
zinc000034125842	-7.61
zinc000036701290	-7.61
nubbe1040	-7.6
nubbe13uff	-7.6
nubbe2198	-7.6
nubbe2516	-7.6
nubbe990	-7.6
perunpdb116	-7.6
fqnp208	-7.6
zinc000000002212	-7.6
nubbe2064	-7.59
nubbe2237	-7.59
nubbe411uff	-7.59
nubbe613uff	-7.59
perunpdb21	-7.59

nubbe1085	-7.58
nubbe1160	-7.58
nubbe553	-7.58
perunpdb112	-7.58
nubbe1142	-7.57
nubbe1710	-7.57
nubbe180	-7.57
nubbe1962	-7.57
nubbe28	-7.57
nubbe834	-7.57
nubbe1507	-7.56
nubbe22	-7.56
nubbe2288	-7.56
nubbe2402	-7.56
nubbe258	-7.56
nubbe93	-7.56
perunpdb217	-7.56
nubbe80uff	-7.55
nubbe1720	-7.54
nubbe202uff	-7.54
nubbe581uff	-7.54
fqnp141	-7.54
zinc000253498282	-7.54
nubbe1053	-7.53
nubbe1175	-7.53
nubbe1248	-7.53
nubbe1458	-7.53
nubbe1626	-7.53
perunpdb241	-7.53
fqnp111	-7.53
zinc000000968375	-7.53
nubbe1459	-7.52
fqnp347	-7.52
zinc000003812897	-7.52
nubbe1151	-7.51
nubbe2243	-7.51
nubbe2279	-7.51
nubbe2293	-7.51
perunpdb242	-7.51
fqnp220	-7.51
zinc000000389747	-7.51
zinc000011617039	-7.51
nubbe1118	-7.5
nubbe1265	-7.5

nubbe1519	-7.5
nubbe1777	-7.5
nubbe2492	-7.5
fqnp324	-7.5
nubbe1054	-7.49
nubbe1286	-7.49
nubbe1320	-7.49
nubbe1424	-7.49
zinc000013129998	-7.49
zinc000096006018	-7.49
nubbe1218	-7.48
nubbe1268	-7.48
nubbe1669	-7.48
nubbe2314	-7.48
nubbe328uff	-7.48
nubbe329uff	-7.48
nubbe396	-7.48
nubbe684	-7.48
zinc000000403533	-7.48
zinc000003945984	-7.48
nubbe1004	-7.47
nubbe1936	-7.47
nubbe216	-7.47
nubbe2359	-7.47
perunpdb111	-7.47
zinc000003876186	-7.47
zinc000003918453	-7.47
zinc000011680067	-7.47
zinc000085536990	-7.47
nubbe1942	-7.46
nubbe2262	-7.46
nubbe2320	-7.46
nubbe2355	-7.46
nubbe607	-7.46
fqnp108	-7.46
zinc000003881640	-7.46
zinc70691889	-7.46
nubbe1828	-7.45
nubbe2229	-7.45
nubbe2267	-7.45
nubbe388uff	-7.45
nubbe584uff	-7.45
nubbe610uff	-7.45
fqnp162	-7.45

fqnp329	-7.45
zinc000094566092	-7.45
zinc68590497	-7.45
nubbe1255	-7.44
nubbe1589	-7.44
nubbe1765	-7.44
nubbe382uff	-7.44
perunpdb101	-7.44
perunpdb146	-7.44
perunpdb76	-7.44
zinc000000004351	-7.44
nubbe1945	-7.43
perunpdb5	-7.43
zinc000002016257	-7.43
zinc000004097467	-7.43
zinc000013512456	-7.43
nubbe1036	-7.42
nubbe2159	-7.42
nubbe2481	-7.42
nubbe1990	-7.41
nubbe2508	-7.41
nubbe79uff	-7.41
fqnp345	-7.41
fqnp402	-7.41
zinc000000643114	-7.41
zinc000256433952	-7.41
nubbe326uff	-7.4
nubbe992	-7.4
perunpdb145	-7.4
fqnp328	-7.4
zinc000053229445	-7.4
zinc000256433946	-7.4
nubbe1210	-7.39
nubbe22uff	-7.39
nubbe591uff	-7.39
perunpdb206	-7.39
fqnp283	-7.39
fqnp426	-7.39
zinc000003876023	-7.39
nubbe400	-7.38
nubbe587	-7.38
nubbe649	-7.38
fqnp380	-7.38

zinc000000896717	-7.38
nubbe1199	-7.37
nubbe13	-7.37
nubbe1714	-7.37
nubbe2070	-7.37
nubbe977	-7.37
perunpdb3	-7.37
nubbe179uff	-7.36
nubbe2009	-7.36
nubbe2189	-7.36
nubbe2351	-7.36
nubbe264	-7.36
nubbe384uff	-7.36
nubbe933	-7.36
perunpdb184	-7.36
zinc000004097440	-7.36
zinc000049643479	-7.36
nubbe2173	-7.35
nubbe228uff	-7.35
nubbe2290	-7.35
nubbe2353	-7.35
nubbe674	-7.35
zinc000004428529	-7.35
zinc000043207238	-7.35
nubbe1055	-7.34
nubbe2261	-7.34
nubbe2289	-7.34
nubbe2376	-7.34
nubbe387	-7.34
perunpdb175	-7.34
fqnp245	-7.34
fqnp87	-7.34
zinc000003807917	-7.34
zinc000022116612	-7.34
nubbe2428	-7.33
nubbe2487	-7.33
nubbe2502	-7.33
perunpdb134	-7.33
zinc000000004076	-7.33
zinc000003993855	-7.33
zinc000253630390	-7.33
nubbe1849	-7.32
nubbe215uff	-7.32

nubbe435	-7.32
perunpdb251	-7.32
fqnp297	-7.32
zinc000000000596	-7.32
zinc000003977981	-7.32
nubbe1329	-7.31
nubbe2496	-7.31
nubbe2515	-7.31
nubbe567	-7.31
zinc000030726187	-7.31
nubbe1426	-7.3
nubbe1719	-7.3
nubbe606	-7.3
perunpdb216	-7.3
fqnp209	-7.3
fqnp232	-7.3
nubbe1451	-7.29
nubbe2174	-7.29
perunpdb149	-7.29
perunpdb228	-7.29
fqnp90	-7.29
zinc000052245489	-7.29
zinc000052955754	-7.29
zinc000150338819	-7.29
nubbe143uff	-7.28
nubbe1460	-7.28
nubbe1730	-7.28
nubbe2155	-7.28
fqnp109	-7.28
zinc000001996117	-7.28
nubbe1726	-7.27
nubbe481	-7.27
fqnp147	-7.27
zinc000000005151	-7.27
nubbe1771	-7.26
nubbe1830	-7.26
nubbe1970	-7.26
nubbe2199	-7.26
nubbe2230	-7.26
perunpdb148	-7.26
fqnp387	-7.26
zinc000000001331	-7.26
zinc000095617678	-7.26
nubbe1150	-7.25

nubbe1193	-7.25
nubbe2493	-7.25
nubbe611	-7.25
zinc000003932831	-7.25
nubbe202	-7.24
nubbe2430	-7.24
nubbe293uff	-7.24
nubbe34	-7.24
nubbe676	-7.24
nubbe82uff	-7.24
nubbe1499	-7.23
nubbe2294	-7.23
zinc000004097308	-7.23
nubbe1234	-7.22
nubbe1361	-7.22
nubbe1442	-7.22
nubbe1555	-7.22
nubbe1717	-7.22
nubbe2231	-7.22
nubbe2387	-7.22
nubbe2486	-7.22
nubbe491	-7.22
nubbe993	-7.22
zinc000003831139	-7.22
nubbe1448	-7.21
nubbe2005	-7.21
nubbe2034	-7.21
nubbe2160	-7.21
nubbe651	-7.21
zinc000000601254	-7.21
perunpdb127	-7.2
nubbe1406	-7.2
nubbe1514	-7.2
nubbe2194	-7.2
nubbe2282	-7.2
nubbe355	-7.2
nubbe460uff	-7.2
perunpdb250	-7.2
fqnp105	-7.2
zinc000012503187	-7.2
nubbe1294	-7.19
nubbe1554	-7.19
nubbe1950	-7.19
nubbe2362	-7.19

nubbe49uff	-7.19
nubbe830uff	-7.19
perunpdb239	-7.19
fqnp363	-7.19
zinc000000509440	-7.19
zinc000006467621	-7.19
nubbe1071	-7.18
nubbe1163	-7.18
nubbe2375	-7.18
nubbe259uff	-7.18
nubbe742uff	-7.18
perunpdb135	-7.18
nubbe1099	-7.17
nubbe1229	-7.17
nubbe1417	-7.17
nubbe1418	-7.17
nubbe1456	-7.17
nubbe1636	-7.17
nubbe1656	-7.17
nubbe2500	-7.17
nubbe565uff	-7.17
nubbe989	-7.17
perunpdb233	-7.17
nubbe1351	-7.16
nubbe1541	-7.16
nubbe1548	-7.16
nubbe159uff	-7.16
nubbe2056	-7.16
nubbe2193	-7.16
nubbe2245	-7.16
nubbe2436	-7.16
nubbe576uff	-7.16
perunpdb100	-7.16
zinc000012503068	-7.16
zinc000021981454	-7.16
nubbe1027	-7.15
nubbe1457	-7.15
nubbe1469	-7.15
nubbe2372	-7.15
nubbe383	-7.15
nubbe570	-7.15
nubbe1412	-7.14
nubbe2082	-7.14

nubbe430	-7.14
perunpdb65	-7.14
fqnp24	-7.14
fqnp284	-7.14
fqnp456	-7.14
nubbe1733	-7.13
nubbe2192	-7.13
nubbe2232	-7.13
nubbe2252	-7.13
nubbe2468	-7.13
nubbe551uff	-7.13
nubbe995	-7.13
perunpdb183	-7.13
fqnp25	-7.13
fqnp346	-7.13
zinc000001539579	-7.13
nubbe2416	-7.12
nubbe554	-7.12
fqnp382	-7.12
fqnp89	-7.12
zinc000003917708	-7.12
nubbe2157	-7.11
nubbe24	-7.11
nubbe2489	-7.11
nubbe856	-7.11
fqnp95	-7.11
zinc000000001587	-7.11
zinc000003782599	-7.11
zinc000005029557	-7.11
zinc000013540519	-7.11
zinc000014164617	-7.11
nubbe105uff	-7.1
nubbe1116	-7.1
nubbe1662	-7.1
nubbe573uff	-7.1
zinc000000002272	-7.1
zinc000003798757	-7.1
zinc000004215736	-7.1
zinc000011726211	-7.1
nubbe667	-7.09
zinc000003778874	-7.09
zinc000096006009	-7.09
nubbe1938	-7.08

nubbe2425	-7.08
nubbe340	-7.08
fqnp425	-7.08
zinc000000007295	-7.08
zinc000003816287	-7.08
nubbe1207	-7.07
nubbe1339	-7.07
nubbe1551	-7.07
nubbe1562	-7.07
nubbe1611	-7.07
nubbe2302	-7.07
nubbe2442	-7.07
nubbe2450	-7.07
nubbe563	-7.07
fqnp388	-7.07
nubbe1415	-7.06
nubbe1603	-7.06
nubbe2190	-7.06
nubbe243uff	-7.06
nubbe255	-7.06
nubbe427	-7.06
perunpdb152	-7.06
zinc000000057255	-7.06
zinc000000968274	-7.06
zinc000084668739	-7.06
nubbe1770	-7.05
nubbe2389	-7.05
nubbe409	-7.05
nubbe435uff	-7.05
nubbe549	-7.05
nubbe587uff	-7.05
fqnp229	-7.05
zinc000000001984	-7.05
zinc000000006156	-7.05
zinc000000120319	-7.05
zinc000001530613	-7.05
nubbe1120	-7.04
nubbe1591	-7.04
nubbe316	-7.04
nubbe988	-7.04
fqnp216	-7.04
zinc000022065398	-7.04
nubbe1939	-7.03
nubbe2260	-7.03

nubbe2385	-7.03
nubbe2424	-7.03
fqnp321	-7.03
zinc000003931840	-7.03
zinc000096903163	-7.03
zinc000100016084	-7.03
nubbe2415	-7.02
nubbe2498	-7.02
nubbe459uff	-7.02
fqnp28	-7.02
fqnp379	-7.02
zinc000003875357	-7.02
zinc000003977767	-7.02
zinc000043100709	-7.02
zinc000245204949	-7.02
nubbe1140	-7.01
nubbe1278	-7.01
nubbe1815	-7.01
nubbe2304	-7.01
perunpdb103	-7.01
perunpdb157	-7.01
fqnp114	-7.01
fqnp214	-7.01
zinc000001999487	-7.01
zinc000003201907	-7.01
zinc000009302201	-7.01
nubbe2227	-7
nubbe571	-7
nubbe859	-7
zinc000003831128	-7
zinc000014263142	-7
nubbe1213	-6.99
nubbe2235	-6.99
nubbe2411	-6.99
nubbe2504	-6.99
perunpdb136	-6.99
fqnp205	-6.99
zinc000035328014	-6.99
nubbe2010	-6.98
nubbe2228	-6.98
nubbe2452	-6.98
fqnp182	-6.98
zinc000012661824	-6.98
zinc000096006024	-6.98

zinc000169621223	-6.98
zinc000203757351	-6.98
nubbe1059	-6.97
nubbe1305	-6.97
nubbe1452	-6.97
nubbe2393	-6.97
nubbe262uff	-6.97
nubbe382	-6.97
perunpdb37	-6.97
fqnp204	-6.97
fqnp348	-6.97
zinc000000601305	-6.97
zinc000012503099	-6.97
zinc000052509366	-6.97
zinc000169344691	-6.97
nubbe1408	-6.96
nubbe1617	-6.96
nubbe168	-6.96
nubbe1739	-6.96
nubbe1948	-6.96
nubbe215	-6.96
nubbe2497	-6.96
nubbe647	-6.96
fqnp55	-6.96
zinc000000001145	-6.96
zinc000000606383	-6.96
nubbe1158	-6.95
nubbe1209	-6.95
nubbe1893	-6.95
nubbe2163	-6.95
nubbe567uff	-6.95
nubbe590	-6.95
fqnp381	-6.95
fqnp448	-6.95
zinc000084589076	-6.95
zinc000100017856	-6.95
nubbe1263	-6.94
nubbe1341	-6.94
nubbe1546	-6.94
nubbe85uff	-6.94
nubbe991	-6.94
zinc000000537931	-6.94
zinc000003815424	-6.94

zinc000011679756	-6.94
nubbe105	-6.93
nubbe1196	-6.93
nubbe1316	-6.93
nubbe1454	-6.93
nubbe1539	-6.93
nubbe1547	-6.93
nubbe1899	-6.93
nubbe198uff	-6.93
nubbe259	-6.93
nubbe305uff	-6.93
nubbe76uff	-6.93
nubbe829	-6.93
perunpdb110	-6.93
perunpdb27	-6.93
zinc000000020221	-6.93
zinc000000025958	-6.93
zinc000004097286	-6.93
nubbe2501	-6.92
nubbe548	-6.92
nubbe576	-6.92
fqnp145	-6.92
zinc000000538550	-6.92
nubbe1003	-6.91
nubbe1212	-6.91
nubbe1314	-6.91
nubbe1420	-6.91
nubbe150uff	-6.91
nubbe1909	-6.91
nubbe1946	-6.91
nubbe2238	-6.91
zinc000035342789	-6.91
nubbe1660	-6.9
nubbe1963	-6.9
nubbe2277	-6.9
nubbe2517	-6.9
perunpdb238	-6.9
fqnp186	-6.9
fqnp26	-6.9
zinc000000001261	-6.9
zinc000000004724	-6.9
zinc000003806721	-6.9
zinc000003830500	-6.9

zinc000003875560	-6.9
nubbe2356	-6.89
nubbe552uff	-6.89
fqnp356	-6.89
zinc000000968310	-6.89
zinc000003938751	-6.89
nubbe1147	-6.88
nubbe1414	-6.88
nubbe1509	-6.88
nubbe1578	-6.88
nubbe1712	-6.88
nubbe1929	-6.88
nubbe571uff	-6.88
nubbe88	-6.88
perunpdb140	-6.88
zinc000100027531	-6.88
nubbe1165	-6.87
nubbe1266	-6.87
nubbe1915	-6.87
nubbe24uff	-6.87
nubbe320uff	-6.87
nubbe356uff	-6.87
nubbe539	-6.87
nubbe586	-6.87
nubbe623	-6.87
perunpdb270	-6.87
zinc000000000347	-6.87
zinc000028973446	-6.87
zinc000035653007	-6.87
zinc000100907004	-6.87
nubbe1449	-6.86
nubbe1587	-6.86
nubbe33uff	-6.86
nubbe664	-6.86
zinc000003797541	-6.86
nubbe1842	-6.85
nubbe2300	-6.85
nubbe252	-6.85
nubbe550	-6.85
nubbe994	-6.85
fqnp244	-6.85
zinc000000000740	-6.85
zinc000000006310	-6.85
zinc000000968233	-6.85

zinc000001530886	-6.85
zinc000001883067	-6.85
nubbe1405	-6.84
nubbe1538	-6.84
nubbe1913	-6.84
nubbe1966	-6.84
nubbe197uff	-6.84
nubbe564	-6.84
nubbe583	-6.84
nubbe986	-6.84
perunpdb102	-6.84
perunpdb252	-6.84
fqnp159	-6.84
zinc000000002279	-6.84
zinc000003812984	-6.84
zinc000019418959	-6.84
nubbe1293	-6.83
nubbe1332	-6.83
nubbe1463	-6.83
nubbe2040	-6.83
nubbe288	-6.83
nubbe940	-6.83
fqnp218	-6.83
fqnp221	-6.83
fqnp234	-6.83
zinc000001612996	-6.83
nubbe1028	-6.82
nubbe2168	-6.82
nubbe2259	-6.82
nubbe2364	-6.82
nubbe350uff	-6.82
nubbe592uff	-6.82
nubbe682	-6.82
perunpdb205	-6.82
zinc000000001016	-6.82
zinc000000121541	-6.82
zinc000013682481	-6.82
zinc000095452610	-6.82
nubbe1411	-6.81
nubbe1709	-6.81
nubbe200uff	-6.81
nubbe2269	-6.81
nubbe2338	-6.81
nubbe2421	-6.81

nubbe264uff	-6.81
perunpdb240	-6.81
zinc000000602632	-6.81
nubbe1057	-6.8
nubbe1358	-6.8
nubbe1570	-6.8
nubbe565	-6.8
nubbe577	-6.8
fqnp219	-6.8
zinc000004097404	-6.8
zinc000095616600	-6.8
nubbe1117	-6.79
nubbe1396	-6.79
nubbe1537	-6.79
nubbe1542	-6.79
nubbe1606	-6.79
nubbe2164	-6.79
nubbe2499	-6.79
nubbe2513	-6.79
nubbe480	-6.79
fqnp241	-6.79
fqnp92	-6.79
zinc000003956788	-6.79
nubbe1325	-6.78
nubbe2510	-6.78
nubbe363	-6.78
fqnp144	-6.78
fqnp376	-6.78
zinc000003830321	-6.78
nubbe1759	-6.77
nubbe1967	-6.77
nubbe201	-6.77
nubbe561uff	-6.77
nubbe575	-6.77
nubbe82	-6.77
nubbe97uff	-6.77
fqnp185	-6.77
fqnp51	-6.77
zinc000001530930	-6.77
zinc000002510358	-6.77
zinc000003925861	-6.77
nubbe1041	-6.76
nubbe143	-6.76

nubbe1508	-6.76
nubbe1610	-6.76
nubbe1732	-6.76
nubbe2023	-6.76
nubbe2054	-6.76
nubbe2088	-6.76
nubbe2098	-6.76
nubbe395uff	-6.76
nubbe574uff	-6.76
nubbe579uff	-6.76
perunpdb62	-6.76
zinc000006858022	-6.76
nubbe1629	-6.75
nubbe2336	-6.75
fqnp228	-6.75
nubbe1138	-6.74
nubbe1241	-6.74
nubbe2268	-6.74
nubbe292uff	-6.74
nubbe569	-6.74
fqnp184	-6.74
zinc000000403566	-6.74
zinc000034608502	-6.74
zinc000085537011	-6.74
zinc000169289388	-6.74
nubbe1069	-6.73
nubbe1113	-6.73
nubbe1598	-6.73
nubbe179	-6.73
nubbe2096	-6.73
nubbe2273	-6.73
nubbe346uff	-6.73
nubbe363uff	-6.73
nubbe764uff	-6.73
perunpdb26	-6.73
perunpdb53	-6.73
fqnp374	-6.73
zinc000000896523	-6.73
zinc000001611274	-6.73
zinc000034220093	-6.73
zinc000043450326	-6.73
nubbe1112	-6.72
nubbe2327	-6.72

nubbe2373	-6.72
nubbe999	-6.72
perunpdb31	-6.72
zinc000000968257	-6.72
zinc000001530948	-6.72
nubbe1227	-6.71
nubbe2213	-6.71
nubbe2449	-6.71
nubbe2465	-6.71
nubbe293	-6.71
nubbe547	-6.71
nubbe85	-6.71
nubbe948	-6.71
fqnp352	-6.71
zinc000000000973	-6.71
zinc000000009073	-6.71
zinc000000601316	-6.71
zinc000003921872	-6.71
zinc000003936683	-6.71
zinc000003977777	-6.71
nubbe1128	-6.7
nubbe1346	-6.7
nubbe1762	-6.7
nubbe1934	-6.7
nubbe1969	-6.7
nubbe199uff	-6.7
nubbe2236	-6.7
nubbe547uff	-6.7
fqnp213	-6.7
fqnp30	-6.7
zinc000003831405	-6.7
nubbe1784	-6.69
nubbe1890	-6.69
nubbe2062	-6.69
nubbe738uff	-6.69
zinc000003812944	-6.69
zinc000100073786	-6.69
zinc000254134439	-6.69
nubbe163	-6.68
nubbe2451	-6.68
nubbe2509	-6.68
nubbe262	-6.68
nubbe608uff	-6.68
nubbe739uff	-6.68

fqnp119	-6.68
fqnp451	-6.68
zinc000000002688	-6.68
zinc000000006157	-6.68
zinc000000402830	-6.68
zinc000000597013	-6.68
zinc000036766734	-6.68
zinc000084757007	-6.68
nubbe1182	-6.67
nubbe1387	-6.67
nubbe1635	-6.67
nubbe1931	-6.67
nubbe2291	-6.67
nubbe2434	-6.67
nubbe551	-6.67
nubbe585	-6.67
nubbe67	-6.67
fqnp223	-6.67
zinc000011677376	-6.67
zinc000064033452	-6.67
nubbe1299	-6.66
nubbe1558	-6.66
nubbe1561	-6.66
nubbe2253	-6.66
nubbe436uff	-6.66
nubbe971	-6.66
fqnp57	-6.66
zinc000000004785	-6.66
zinc000019156872	-6.66
nubbe1575	-6.65
nubbe1927	-6.65
nubbe2514	-6.65
nubbe355uff	-6.65
nubbe588	-6.65
perunpdb151	-6.65
fqnp22	-6.65
fqnp450	-6.65
fqnp46	-6.65
zinc000100014475	-6.65
nubbe145	-6.64
nubbe1922	-6.64
nubbe2065	-6.64
nubbe2069	-6.64
nubbe613	-6.64

nubbe622	-6.64
nubbe634	-6.64
zinc000001530968	-6.64
zinc000005764759	-6.64
nubbe1394	-6.63
nubbe1593	-6.63
nubbe1648	-6.63
nubbe662	-6.63
fqnp258	-6.63
fqnp278	-6.63
fqnp292	-6.63
fqnp367	-6.63
zinc000000000903	-6.63
zinc000000538621	-6.63
zinc000001530697	-6.63
zinc000003873296	-6.63
nubbe1230	-6.62
nubbe1612	-6.62
nubbe1822	-6.62
nubbe1996	-6.62
nubbe854	-6.62
perunpdb237	-6.62
perunpdb98	-6.62
fqnp91	-6.62
zinc000002570895	-6.62
zinc000003872277	-6.62
zinc000013537284	-6.62
zinc000019796158	-6.62
nubbe1630	-6.61
nubbe2306	-6.61
fqnp302	-6.61
fqnp435	-6.61
zinc000000896703	-6.61
zinc000003872931	-6.61
zinc000004212851	-6.61
zinc000004214603	-6.61
zinc000040899447	-6.61
zinc000100036536	-6.61
nubbe1188	-6.6
nubbe163uff	-6.6
nubbe2079	-6.6
nubbe2165	-6.6
nubbe471	-6.6

nubbe488uff	-6.6
nubbe643uff	-6.6
nubbe683	-6.6
nubbe733	-6.6
nubbe77uff	-6.6
fqnp164	-6.6
fqnp170	-6.6
zinc000000601229	-6.6
zinc000003812983	-6.6
nubbe1083	-6.59
nubbe1769	-6.59
nubbe577uff	-6.59
nubbe612uff	-6.59
nubbe663	-6.59
perunpdb132	-6.59
zinc000000003876	-6.59
zinc000000537822	-6.59
zinc000000896560	-6.59
nubbe1543	-6.58
nubbe1576	-6.58
nubbe1997	-6.58
nubbe2341	-6.58
nubbe2352	-6.58
nubbe2419	-6.58
nubbe323uff	-6.58
nubbe569uff	-6.58
nubbe570uff	-6.58
zinc000000004840	-6.58
zinc000000897232	-6.58
zinc000004083606	-6.58
zinc000043200832	-6.58
zinc000085205448	-6.58
nubbe1713	-6.57
nubbe1968	-6.57
nubbe320	-6.57
nubbe399uff	-6.57
nubbe542uff	-6.57
nubbe572uff	-6.57
nubbe573	-6.57
nubbe944	-6.57
nubbe996	-6.57
perunpdb118	-6.57
perunpdb123	-6.57

perunpdb165	-6.57
perunpdb61	-6.57
zinc000000001370	-6.57
zinc000000491073	-6.57
zinc000000896557	-6.57
zinc000001481956	-6.57
zinc000003872055	-6.57
zinc000003876136	-6.57
zinc000003914810	-6.57
zinc000007997952	-6.57
nubbe1647	-6.56
nubbe2369	-6.56
nubbe549uff	-6.56
nubbe648	-6.56
fqnp110	-6.56
fqnp191	-6.56
zinc000001481815	-6.56
zinc000001530599	-6.56
zinc000003824921	-6.56
zinc000003993846	-6.56
zinc000019594557	-6.56
zinc000150588351	-6.56
nubbe1092	-6.55
nubbe1326	-6.55
nubbe139uff	-6.55
nubbe201uff	-6.55
nubbe30uff	-6.55
nubbe380	-6.55
nubbe436	-6.55
nubbe532uff	-6.55
nubbe855	-6.55
nubbe937	-6.55
perunpdb105	-6.55
perunpdb271	-6.55
fqnp317	-6.55
fqnp358	-6.55
zinc000000014257	-6.55
zinc000003830993	-6.55
zinc000004474414	-6.55
zinc000014961096	-6.55
nubbe122uff	-6.54
nubbe126	-6.54
nubbe1496	-6.54
nubbe150	-6.54

nubbe1569	-6.54
nubbe1941	-6.54
nubbe2008	-6.54
nubbe2051	-6.54
nubbe2271	-6.54
nubbe483	-6.54
perunpdb115	-6.54
zinc000000000655	-6.54
zinc000000005560	-6.54
zinc000001530761	-6.54
zinc000018089317	-6.54
zinc000019203912	-6.54
zinc000103105084	-6.54
nubbe1513	-6.53
nubbe1850	-6.53
nubbe2201	-6.53
nubbe2317	-6.53
nubbe433	-6.53
nubbe562	-6.53
nubbe611uff	-6.53
perunpdb109	-6.53
perunpdb139	-6.53
zinc000000538273	-6.53
zinc000004475353	-6.53
nubbe1195	-6.52
nubbe1422	-6.52
nubbe234	-6.52
nubbe685	-6.52
perunpdb189	-6.52
zinc000000001681	-6.52
zinc000000075126	-6.52
zinc000000968263	-6.52
zinc000003875368	-6.52
zinc000040430143	-6.52
nubbe1264	-6.51
nubbe156uff	-6.51
nubbe79	-6.51
zinc000000538275	-6.51
zinc000001548097	-6.51
zinc000003871541	-6.51
zinc000003875484	-6.51
zinc000004214700	-6.51
zinc000006409735	-6.51
zinc000252679615	-6.51

nubbe1492	-6.5
nubbe165	-6.5
nubbe1824	-6.5
nubbe1930	-6.5
nubbe2158	-6.5
nubbe572	-6.5
nubbe591	-6.5
nubbe983	-6.5
fqnp288	-6.5
zinc000000119717	-6.5
zinc000002032615	-6.5
zinc000003871701	-6.5
zinc04011310	-6.5
nubbe1177	-6.49
nubbe1277	-6.49
nubbe1404	-6.49
nubbe1818	-6.49
nubbe1965	-6.49
nubbe2017	-6.49
nubbe2195	-6.49
nubbe246uff	-6.49
nubbe427uff	-6.49
nubbe477	-6.49
nubbe49	-6.49
nubbe568uff	-6.49
nubbe830	-6.49
zinc000001542199	-6.49
zinc000002548959	-6.49
zinc000003978005	-6.49
zinc000100037020	-6.49
zinc95993817	-6.49
nubbe1526	-6.48
nubbe2066	-6.48
perunpdb39	-6.48
zinc000003792789	-6.48
zinc000009212654	-6.48
zinc000021303210	-6.48
nubbe1285	-6.47
nubbe1904	-6.47
nubbe2046	-6.47
nubbe2348	-6.47
nubbe394	-6.47
nubbe564uff	-6.47

nubbe575uff	-6.47
nubbe610	-6.47
nubbe612	-6.47
perunpdb104	-6.47
fqnp253	-6.47
fqnp357	-6.47
zinc000000001773	-6.47
zinc000003800008	-6.47
zinc000003820029	-6.47
nubbe1621	-6.46
nubbe1708	-6.46
nubbe177	-6.46
nubbe1782	-6.46
nubbe2035	-6.46
nubbe2053	-6.46
nubbe2483	-6.46
nubbe539uff	-6.46
nubbe677	-6.46
zinc000169621215	-6.46
nubbe1044	-6.45
nubbe1161	-6.45
nubbe1991	-6.45
nubbe217uff	-6.45
nubbe406	-6.45
nubbe490	-6.45
nubbe906	-6.45
perunpdb244	-6.45
fqnp403	-6.45
zinc000000002191	-6.45
zinc04011308	-6.45
nubbe1275	-6.44
nubbe1319	-6.44
nubbe1724	-6.44
nubbe2003	-6.44
nubbe2071	-6.44
nubbe2478	-6.44
nubbe327uff	-6.44
perunpdb2	-6.44
fqnp254	-6.44
zinc000000897240	-6.44
nubbe1338	-6.43
nubbe1423	-6.43
nubbe1468	-6.43

nubbe1528	-6.43
fqnp117	-6.43
fqnp63	-6.43
zinc000000003642	-6.43
zinc000000006251	-6.43
zinc000003874498	-6.43
zinc000100015048	-6.43
zinc000118913560	-6.43
nubbe1494	-6.42
nubbe1516	-6.42
nubbe2412	-6.42
nubbe2413	-6.42
nubbe34auff	-6.42
nubbe998	-6.42
zinc000000020228	-6.42
zinc000000057534	-6.42
zinc000001530935	-6.42
zinc000003830999	-6.42
zinc000004212809	-6.42
nubbe1654	-6.41
nubbe178	-6.41
nubbe1933	-6.41
nubbe1943	-6.41
nubbe2256	-6.41
nubbe67uff	-6.41
fqnp249	-6.41
fqnp354	-6.41
zinc89929685	-6.41
nubbe10	-6.4
nubbe1149	-6.4
nubbe1330	-6.4
nubbe1440	-6.4
nubbe152	-6.4
nubbe1524	-6.4
nubbe1716	-6.4
nubbe1758	-6.4
nubbe2212	-6.4
nubbe579	-6.4
nubbe78uff	-6.4
nubbe860	-6.4
zinc000000968326	-6.4
zinc000001530625	-6.4
zinc000030691679	-6.4
zinc000084441937	-6.4

zinc000100003200	-6.4
nubbe1337	-6.39
nubbe199	-6.39
nubbe2284	-6.39
nubbe597	-6.39
nubbe967	-6.39
perunpdb138	-6.39
fqnp2	-6.39
nubbe1274	-6.38
nubbe1556	-6.38
nubbe220uff	-6.38
nubbe368	-6.38
nubbe586uff	-6.38
perunpdb164	-6.38
fqnp289	-6.38
zinc000000001958	-6.38
zinc000003875334	-6.38
zinc000018203737	-6.38
zinc000022002214	-6.38
zinc000030691420	-6.38
nubbe1256	-6.37
nubbe164	-6.37
nubbe1988	-6.37
nubbe2223	-6.37
nubbe33	-6.37
nubbe411	-6.37
nubbe532	-6.37
nubbe644	-6.37
nubbe852	-6.37
perunpdb122	-6.37
perunpdb133	-6.37
fqnp215	-6.37
zinc000004097476	-6.37
zinc000005752191	-6.37
nubbe1103	-6.36
nubbe1270	-6.36
nubbe1350	-6.36
nubbe1545	-6.36
nubbe189	-6.36
nubbe1937	-6.36
nubbe377	-6.36
perunpdb235	-6.36
fqnp113	-6.36
fqnp23	-6.36

zinc000000599734	-6.36
zinc000000601283	-6.36
zinc000000896755	-6.36
zinc000001530571	-6.36
zinc000003813083	-6.36
zinc000222731806	-6.36
zinc39105026	-6.36
nubbe1467	-6.35
nubbe2089	-6.35
nubbe325	-6.35
nubbe538uff	-6.35
fqnp86	-6.35
zinc000000000456	-6.35
nubbe1428	-6.34
nubbe1715	-6.34
nubbe1780	-6.34
nubbe2061	-6.34
nubbe2090	-6.34
perunpdb125	-6.34
zinc000000097996	-6.34
zinc000000897002	-6.34
zinc000001484334	-6.34
zinc000003786192	-6.34
zinc000028973441	-6.34
zinc000095626706	-6.34
zinc000148723177	-6.34
nubbe1090	-6.33
nubbe1734	-6.33
nubbe2180	-6.33
nubbe2209	-6.33
nubbe2384	-6.33
nubbe76	-6.33
perunpdb124	-6.33
fqnp385	-6.33
zinc000000000941	-6.33
zinc000000020245	-6.33
zinc000000968327	-6.33
zinc000003812933	-6.33
zinc000005162311	-6.33
nubbe1493	-6.32
nubbe1756	-6.32
nubbe1919	-6.32
nubbe221uff	-6.32

nubbe2438	-6.32
fqnp187	-6.32
zinc000000001979	-6.32
zinc000000402909	-6.32
zinc000100032379	-6.32
nubbe1315	-6.31
nubbe1573	-6.31
nubbe508	-6.31
nubbe554uff	-6.31
nubbe581	-6.31
perunpdb174	-6.31
zinc000003914808	-6.31
zinc000004175630	-6.31
zinc000030691736	-6.31
nubbe1599	-6.3
nubbe1947	-6.3
nubbe563uff	-6.3
zinc000003831282	-6.3
zinc000100004343	-6.3
nubbe1197	-6.29
nubbe1911	-6.29
nubbe1940	-6.29
nubbe2242	-6.29
nubbe2316	-6.29
nubbe2431	-6.29
nubbe826uff	-6.29
nubbe869	-6.29
perunpdb143	-6.29
fqnp27	-6.29
zinc000000113426	-6.29
zinc000000896595	-6.29
zinc000053683151	-6.29
nubbe1352	-6.28
nubbe1388	-6.28
nubbe144	-6.28
nubbe1527	-6.28
nubbe2255	-6.28
nubbe2381	-6.28
nubbe464	-6.28
zinc000003914809	-6.28
zinc000003960338	-6.28
zinc000019361042	-6.28
nubbe1247	-6.27

nubbe1564	-6.27
nubbe1577	-6.27
nubbe1582	-6.27
nubbe2448	-6.27
nubbe402uff	-6.27
perunpdb52	-6.27
zinc000012414057	-6.27
zinc000096014710	-6.27
zinc000169289411	-6.27
nubbe1252	-6.26
nubbe1400	-6.26
nubbe1693	-6.26
nubbe2204	-6.26
nubbe269uff	-6.26
nubbe584	-6.26
nubbe600uff	-6.26
nubbe858	-6.26
fqnp351	-6.26
zinc000000000096	-6.26
zinc000001530725	-6.26
zinc000004212854	-6.26
nubbe1007	-6.25
nubbe1089	-6.25
nubbe1172	-6.25
nubbe1269	-6.25
nubbe1615	-6.25
nubbe1855	-6.25
nubbe2052	-6.25
nubbe507	-6.25
nubbe561	-6.25
nubbe943	-6.25
nubbe1291	-6.24
nubbe1665	-6.24
nubbe1944	-6.24
nubbe200	-6.24
nubbe218	-6.24
nubbe598	-6.24
fqnp377	-6.24
zinc000000000323	-6.24
nubbe1159	-6.23
nubbe1267	-6.23
nubbe1334	-6.23
nubbe1401	-6.23
nubbe1766	-6.23

nubbe2087	-6.23
nubbe432uff	-6.23
fqnp298	-6.23
zinc000100018854	-6.23
nubbe1137	-6.22
nubbe1462	-6.22
nubbe182uff	-6.22
nubbe593	-6.22
nubbe833	-6.22
nubbe935	-6.22
fqnp342	-6.22
zinc000003875332	-6.22
zinc000008214625	-6.22
zinc000012859773	-6.22
zinc000218037687	-6.22
nubbe1427	-6.21
nubbe1465	-6.21
nubbe151	-6.21
nubbe1574	-6.21
nubbe1718	-6.21
nubbe1776	-6.21
nubbe1960	-6.21
nubbe2012	-6.21
nubbe2172	-6.21
nubbe2292	-6.21
nubbe2298	-6.21
nubbe376uff	-6.21
nubbe511	-6.21
perunpdb234	-6.21
fqnp293	-6.21
fqnp85	-6.21
zinc000003782818	-6.21
zinc000003798734	-6.21
zinc000003920657	-6.21
zinc000100036924	-6.21
nubbe1302	-6.2
nubbe1441	-6.2
nubbe149	-6.2
nubbe158uff	-6.2
nubbe2187	-6.2
nubbe344	-6.2
nubbe942	-6.2
perunpdb249	-6.2
fqnp247	-6.2

fqnp37	-6.2
zinc000000006300	-6.2
zinc000001530812	-6.2
zinc000003873832	-6.2
zinc000003978006	-6.2
zinc000011681534	-6.2
zinc000019594599	-6.2
zinc000100003201	-6.2
zinc89929684	-6.2
nubbe1340	-6.19
nubbe1559	-6.19
nubbe1898	-6.19
nubbe68uff	-6.19
nubbe907	-6.19
nubbe957	-6.19
zinc000000000509	-6.19
zinc000000004778	-6.19
zinc000001530947	-6.19
nubbe1910	-6.18
nubbe1932	-6.18
nubbe2184	-6.18
nubbe2299	-6.18
nubbe2337	-6.18
nubbe261	-6.18
nubbe276uff	-6.18
nubbe401uff	-6.18
nubbe614uff	-6.18
nubbe645uff	-6.18
zinc000000000905	-6.18
zinc000000013156	-6.18
zinc000000113428	-6.18
zinc000001530977	-6.18
zinc000003875392	-6.18
zinc000003938746	-6.18
zinc000004099032	-6.18
zinc000017146904	-6.18
nubbe1276	-6.17
nubbe156	-6.17
nubbe160	-6.17
nubbe1846	-6.17
nubbe325uff	-6.17
nubbe58	-6.17
nubbe617	-6.17

fqnp371	-6.17
zinc000003823475	-6.17
zinc000009224016	-6.17
zinc000253632968	-6.17
nubbe2447	-6.16
nubbe2506	-6.16
nubbe376	-6.16
nubbe509	-6.16
nubbe964	-6.16
zinc000000113410	-6.16
zinc000003775644	-6.16
zinc000003830986	-6.16
zinc000095619105	-6.16
zinc000100036751	-6.16
zinc62454966	-6.16
nubbe1008	-6.15
nubbe1014	-6.15
nubbe1189	-6.15
nubbe1450	-6.15
nubbe1466	-6.15
nubbe149uff	-6.15
nubbe165uff	-6.15
nubbe2025	-6.15
nubbe2170	-6.15
nubbe2358	-6.15
nubbe252uff	-6.15
nubbe600	-6.15
nubbe965	-6.15
perunpdb258	-6.15
fqnp93	-6.15
fqnp97	-6.15
zinc000000968275	-6.15
zinc000003787097	-6.15
zinc000068202099	-6.15
zinc000169621228	-6.15
nubbe2037	-6.14
nubbe2067	-6.14
nubbe2485	-6.14
nubbe306uff	-6.14
nubbe533uff	-6.14
nubbe650	-6.14
perunpdb32	-6.14
fqnp280	-6.14

fqnp340	-6.14
fqnp343	-6.14
zinc000000004448	-6.14
zinc000000968256	-6.14
nubbe1224	-6.13
nubbe1239	-6.13
nubbe1700	-6.13
nubbe1735	-6.13
nubbe1767	-6.13
nubbe181uff	-6.13
nubbe2399	-6.13
nubbe450	-6.13
nubbe5	-6.13
fqnp355	-6.13
zinc000000967521	-6.13
zinc000003604264	-6.13
zinc000003875439	-6.13
zinc000009302239	-6.13
zinc000019796018	-6.13
nubbe1544	-6.12
nubbe1814	-6.12
nubbe1851	-6.12
nubbe267	-6.12
nubbe533	-6.12
zinc000000896569	-6.12
zinc000005456939	-6.12
nubbe1623	-6.11
nubbe1694	-6.11
nubbe2382	-6.11
nubbe2383	-6.11
nubbe617uff	-6.11
nubbe865	-6.11
fqnp296	-6.11
zinc000000002647	-6.11
zinc000003831429	-6.11
nubbe1500	-6.1
nubbe1560	-6.1
nubbe2340	-6.1
nubbe426	-6.1
nubbe592	-6.1
perunpdb162	-6.1
perunpdb209	-6.1
fqnp175	-6.1
fqnp227	-6.1

fqnp96	-6.1
zinc000000896711	-6.1
zinc000035999642	-6.1
zinc000066166864	-6.1
zinc000095616937	-6.1
zinc000101489663	-6.1
nubbe1783	-6.09
nubbe1903	-6.09
nubbe2019	-6.09
nubbe761uff	-6.09
perunpdb64	-6.09
zinc75627230	-6.09
nubbe1831	-6.08
nubbe2339	-6.08
nubbe580uff	-6.08
fqnp15	-6.08
fqnp350	-6.08
fqnp408	-6.08
zinc000000044027	-6.08
zinc000058581064	-6.08
zinc82876574	-6.08
nubbe1016	-6.07
nubbe1279	-6.07
nubbe1372	-6.07
nubbe1549	-6.07
nubbe1607	-6.07
nubbe598uff	-6.07
nubbe828	-6.07
nubbe936	-6.07
fqnp248	-6.07
fqnp465	-6.07
zinc000000001728	-6.07
zinc000004235575	-6.07
zinc000004632106	-6.07
zinc000013763987	-6.07
zinc000022116608	-6.07
zinc000051951647	-6.07
nubbe1272	-6.06
nubbe1529	-6.06
nubbe1924	-6.06
nubbe1935	-6.06
nubbe1995	-6.06
nubbe2074	-6.06
nubbe2266	-6.06

nubbe2401	-6.06
nubbe434	-6.06
zinc000000527386	-6.06
zinc000019702309	-6.06
nubbe1206	-6.05
nubbe2443	-6.05
nubbe2490	-6.05
nubbe285uff	-6.05
nubbe488	-6.05
nubbe520	-6.05
nubbe566uff	-6.05
perunpdb141	-6.05
zinc000000000346	-6.05
zinc000000006427	-6.05
zinc000001530688	-6.05
zinc000003831430	-6.05
zinc000004228257	-6.05
nubbe1022	-6.04
nubbe1121	-6.04
nubbe158	-6.04
nubbe2391	-6.04
nubbe23uff	-6.04
nubbe2418	-6.04
nubbe929	-6.04
nubbe945	-6.04
perunpdb260	-6.04
zinc000002008866	-6.04
zinc000005733652	-6.04
zinc000150601177	-6.04
zinc62454097	-6.04
nubbe10uff	-6.03
nubbe1205	-6.03
nubbe1592	-6.03
nubbe1723	-6.03
nubbe2024	-6.03
nubbe2325	-6.03
nubbe245uff	-6.03
nubbe2505	-6.03
nubbe294uff	-6.03
nubbe32	-6.03
nubbe375	-6.03
nubbe609uff	-6.03
perunpdb117	-6.03

fqnp115	-6.03
fqnp21	-6.03
fqnp301	-6.03
zinc000003872738	-6.03
zinc000003992105	-6.03
zinc000019362735	-6.03
zinc62454585	-6.03
nubbe1009	-6.02
nubbe120	-6.02
nubbe1273	-6.02
nubbe1540	-6.02
nubbe1563	-6.02
nubbe1872	-6.02
nubbe317	-6.02
nubbe345uff	-6.02
nubbe665	-6.02
nubbe672	-6.02
perunpdb261	-6.02
fqnp312	-6.02
zinc000001530741	-6.02
zinc000001542930	-6.02
zinc39105036	-6.02
nubbe1228	-6.01
nubbe1324	-6.01
nubbe1856	-6.01
nubbe217	-6.01
nubbe2215	-6.01
nubbe2275	-6.01
nubbe2420	-6.01
nubbe350	-6.01
nubbe827uff	-6.01
perunpdb269	-6.01
fqnp299	-6.01
fqnp35	-6.01
zinc000003920028	-6.01
nubbe1225	-6
nubbe1742	-6
nubbe1812	-6
nubbe1836	-6
nubbe616uff	-6
nubbe81	-6
perunpdb60	-6
fqnp316	-6

zinc000000643153	-6
zinc000001997127	-6
zinc000004693574	-6
zinc000019362737	-6
nubbe1097	-5.99
nubbe122	-5.99
nubbe14	-5.99
nubbe2014	-5.99
nubbe2100	-5.99
nubbe2131	-5.99
nubbe251uff	-5.99
nubbe409uff	-5.99
nubbe739	-5.99
perunpdb180	-5.99
perunpdb97	-5.99
fqnp1	-5.99
fqnp134	-5.99
zinc000000000575	-5.99
zinc000000001175	-5.99
zinc000000113404	-5.99
zinc000001853550	-5.99
zinc000008383240	-5.99
nubbe250	-5.98
fqnp171	-5.98
fqnp263	-5.98
fqnp444	-5.98
zinc000000000853	-5.98
zinc000000896731	-5.98
zinc000003830218	-5.98
zinc000034806477	-5.98
nubbe295	-5.97
nubbe373	-5.97
nubbe393	-5.97
nubbe440uff	-5.97
perunpdb1	-5.97
fqnp314	-5.97
zinc000003831586	-5.97
zinc000004629876	-5.97
zinc000022002218	-5.97
zinc000100070954	-5.97
nubbe1023	-5.96
nubbe1303	-5.96
nubbe219	-5.96
nubbe2334	-5.96
fqnp360	-5.96
zinc000000000271	-5.96
zinc000000056427	-5.96
zinc000000896819	-5.96
zinc82876572	-5.96
nubbe125	-5.95
nubbe1323	-5.95
nubbe1501	-5.95
nubbe1585	-5.95
nubbe1957	-5.95
nubbe2361	-5.95
nubbe299uff	-5.95
nubbe306	-5.95
nubbe348uff	-5.95
nubbe358uff	-5.95
nubbe749uff	-5.95
nubbe83	-5.95
fqnp14	-5.95
fqnp361	-5.95
zinc000000120286	-5.95
zinc000000537891	-5.95
zinc000003776633	-5.95
zinc000003830215	-5.95
zinc000043763856	-5.95
zinc000068153186	-5.95
nubbe18	-5.94
nubbe2099	-5.94
nubbe249	-5.94
nubbe686	-5.94
nubbe950	-5.94
perunpdb274	-5.94
fqnp454	-5.94
zinc000000002216	-5.94
zinc000000967520	-5.94
zinc000001690324	-5.94
nubbe120uff	-5.93
nubbe1368	-5.93
nubbe1445	-5.93
nubbe152uff	-5.93
nubbe1844	-5.93
nubbe1896	-5.93
nubbe203uff	-5.93
nubbe2101	-5.93
nubbe292	-5.93

nubbe609	-5.93
perunpdb79	-5.93
fqnp42	-5.93
zinc000000020220	-5.93
zinc000000897301	-5.93
zinc000001895505	-5.93
zinc000003920027	-5.93
nubbe1590	-5.92
nubbe1768	-5.92
nubbe219uff	-5.92
nubbe672uff	-5.92
nubbe866	-5.92
perunpdb273	-5.92
zinc000000018635	-5.92
zinc000001530618	-5.92
zinc39105037	-5.92
zinc72321646	-5.92
nubbe1244	-5.91
nubbe1331	-5.91
nubbe1373	-5.91
nubbe1852	-5.91
nubbe2214	-5.91
nubbe2224	-5.91
nubbe266uff	-5.91
nubbe853	-5.91
perunpdb33	-5.91
fqnp16	-5.91
fqnp60	-5.91
zinc000000001148	-5.91
zinc000022010379	-5.91
nubbe126uff	-5.9
nubbe1280	-5.9
nubbe1429	-5.9
nubbe2154	-5.9
nubbe2445	-5.9
nubbe2521	-5.9
nubbe465	-5.9
nubbe582uff	-5.9
nubbe871	-5.9
fqnp419	-5.9
fqnp98	-5.9
zinc000002570817	-5.9
zinc000003808779	-5.9

zinc000003938686	-5.9
zinc000003940470	-5.9
zinc000004693575	-5.9
zinc000022010375	-5.9
zinc000027990463	-5.9
nubbe1306	-5.89
nubbe1658	-5.89
nubbe287uff	-5.89
nubbe352	-5.89
nubbe6	-5.89
perunpdb218	-5.89
fqnp183	-5.89
fqnp242	-5.89
fqnp34	-5.89
zinc000000089763	-5.89
zinc000003964126	-5.89
zinc000006745272	-5.89
zinc000022010387	-5.89
zinc000072316335	-5.89
zinc000100296828	-5.89
nubbe161	-5.88
nubbe171uff	-5.88
nubbe178uff	-5.88
nubbe2055	-5.88
nubbe2225	-5.88
nubbe483uff	-5.88
perunpdb119	-5.88
fqnp341	-5.88
fqnp449	-5.88
zinc000000001792	-5.88
zinc000000002281	-5.88
zinc000000601301	-5.88
zinc000003812989	-5.88
zinc000100014909	-5.88
zinc000150338755	-5.88
nubbe1056	-5.87
nubbe1487	-5.87
nubbe1522	-5.87
nubbe164uff	-5.87
nubbe1659	-5.87
nubbe1695	-5.87
nubbe30	-5.87
nubbe345	-5.87

nubbe645	-5.87
nubbe849	-5.87
fqnp392	-5.87
zinc000000121480	-5.87
zinc000000538564	-5.87
zinc000001530863	-5.87
zinc000004097304	-5.87
zinc000019796155	-5.87
zinc39105187	-5.87
nubbe1920	-5.86
nubbe2026	-5.86
nubbe2511	-5.86
nubbe2518	-5.86
nubbe287	-5.86
nubbe669	-5.86
nubbe829uff	-5.86
nubbe98uff	-5.86
fqnp252	-5.86
zinc000000008667	-5.86
zinc000000020248	-5.86
zinc000003818808	-5.86
zinc000003927198	-5.86
zinc000004099200	-5.86
zinc000052957434	-5.86
nubbe1013	-5.85
nubbe1119	-5.85
nubbe1436	-5.85
nubbe1588	-5.85
nubbe2153	-5.85
nubbe2283	-5.85
nubbe2388	-5.85
nubbe347uff	-5.85
nubbe752uff	-5.85
nubbe972	-5.85
perunpdb188	-5.85
perunpdb236	-5.85
perunpdb257	-5.85
perunpdb259	-5.85
fqnp128	-5.85
zinc000001554274	-5.85
nubbe1211	-5.84
nubbe141	-5.84
nubbe2030	-5.84
nubbe234uff	-5.84

nubbe2427	-5.84
nubbe2444	-5.84
nubbe2479	-5.84
nubbe276	-5.84
nubbe319	-5.84
nubbe481uff	-5.84
nubbe926	-5.84
perunpdb153	-5.84
perunpdb63	-5.84
fqnp29	-5.84
fqnp310	-5.84
fqnp404	-5.84
zinc000000057522	-5.84
zinc000000105216	-5.84
zinc000000113446	-5.84
zinc000000607971	-5.84
zinc000003819392	-5.84
zinc000003830391	-5.84
zinc000003831404	-5.84
zinc000004640636	-5.84
zinc000035902489	-5.84
zinc000053084692	-5.84
zinc000100006264	-5.84
zinc000100018594	-5.84
nubbe159	-5.83
nubbe1673	-5.83
nubbe220	-5.83
nubbe399	-5.83
perunpdb204	-5.83
fqnp259	-5.83
zinc000000621893	-5.83
zinc000000896918	-5.83
zinc000001530981	-5.83
zinc000003977978	-5.83
zinc000012503076	-5.83
nubbe1301	-5.82
nubbe1923	-5.82
nubbe2177	-5.82
nubbe2310	-5.82
nubbe2345	-5.82
nubbe28uff	-5.82
nubbe828uff	-5.82
fqnp304	-5.82
zinc000001530929	-5.82

zinc000001851149	-5.82
zinc000003830961	-5.82
zinc000012484958	-5.82
zinc000014210455	-5.82
zinc04011309	-5.82
nubbe1639	-5.81
nubbe1912	-5.81
nubbe1986	-5.81
nubbe550uff	-5.81
nubbe596uff	-5.81
nubbe633uff	-5.81
fqnp131	-5.81
zinc000000020243	-5.81
zinc000000113398	-5.81
zinc000000897222	-5.81
zinc000002036848	-5.81
zinc000006733300	-5.81
zinc000019875504	-5.81
zinc000033943508	-5.81
nubbe1371	-5.8
nubbe1472	-5.8
nubbe1586	-5.8
nubbe1772	-5.8
nubbe2210	-5.8
nubbe2315	-5.8
nubbe514	-5.8
nubbe737uff	-5.8
nubbe909	-5.8
perunpdb264	-5.8
fqnp146	-5.8
zinc000000601253	-5.8
zinc000002016258	-5.8
zinc000003964325	-5.8
zinc000049036447	-5.8
zinc82876580	-5.8
nubbe1	-5.79
nubbe1597	-5.79
nubbe1788	-5.79
nubbe2001	-5.79
nubbe54	-5.79
fqnp256	-5.79
zinc000003826253	-5.79
zinc000008552123	-5.79
zinc000013911941	-5.79
nubbe1335	-5.78
nubbe153	-5.78
nubbe2045	-5.78
nubbe27	-5.78
nubbe644uff	-5.78
zinc000000000449	-5.78
zinc000000035804	-5.78
zinc000000607790	-5.78
zinc000001530726	-5.78
zinc000001530776	-5.78
zinc000001997125	-5.78
zinc000003816292	-5.78
zinc000072481720	-5.78
nubbe1020	-5.77
nubbe1596	-5.77
nubbe2080	-5.77
nubbe2397	-5.77
nubbe305	-5.77
nubbe973	-5.77
perunpdb121	-5.77
zinc000000001655	-5.77
zinc76631025	-5.77
nubbe1058	-5.76
nubbe1366	-5.76
nubbe154uff	-5.76
nubbe1834	-5.76
nubbe1840	-5.76
nubbe2440	-5.76
nubbe2441	-5.76
nubbe36uff	-5.76
nubbe601	-5.76
nubbe615uff	-5.76
nubbe629	-5.76
nubbe681	-5.76
fqnp362	-5.76
zinc000000009689	-5.76
zinc000000057313	-5.76
zinc000000083315	-5.76
zinc000003871960	-5.76
zinc000016052277	-5.76
zinc000022010382	-5.76
nubbe1173	-5.75

nubbe1281	-5.75
nubbe1403	-5.75
nubbe1605	-5.75
nubbe2519	-5.75
nubbe291uff	-5.75
fqnp255	-5.75
fqnp36	-5.75
zinc000000004321	-5.75
zinc000000897229	-5.75
zinc000003875980	-5.75
zinc000007997568	-5.75
zinc000034051848	-5.75
zinc000084843283	-5.75
zinc000095915244	-5.75
zinc00051800	-5.75
nubbe1419	-5.74
nubbe1517	-5.74
nubbe2059	-5.74
nubbe2346	-5.74
nubbe25	-5.74
fqnp52	-5.74
zinc000000000751	-5.74
zinc000100018598	-5.74
nubbe1223	-5.73
nubbe1622	-5.73
nubbe1887	-5.73
nubbe2020	-5.73
zinc000000000931	-5.73
zinc000003810860	-5.73
zinc000004618208	-5.73
nubbe1435	-5.72
nubbe1461	-5.72
nubbe181	-5.72
nubbe2105	-5.72
nubbe2276	-5.72
nubbe666	-5.72
nubbe827	-5.72
nubbe851	-5.72
fqnp172	-5.72
fqnp459	-5.72
zinc000000000850	-5.72
zinc000000001267	-5.72
zinc000000002159	-5.72
zinc000003830579	-5.72

zinc000013545634	-5.72
zinc000014210457	-5.72
nubbe2049	-5.71
nubbe421	-5.71
perunpdb35	-5.71
fqnp102	-5.71
zinc000000000128	-5.71
zinc000000020250	-5.71
zinc000003780893	-5.71
zinc000003831490	-5.71
zinc000084400879	-5.71
zinc39105035	-5.71
nubbe1073	-5.7
nubbe2186	-5.7
nubbe297uff	-5.7
nubbe353	-5.7
nubbe740uff	-5.7
nubbe982	-5.7
perunpdb107	-5.7
perunpdb108	-5.7
fqnp353	-5.7
zinc000003861806	-5.7
zinc000003972949	-5.7
zinc000019594594	-5.7
zinc000204073689	-5.7
nubbe1633	-5.69
nubbe1749	-5.69
nubbe405	-5.69
nubbe83uff	-5.69
fqnp194	-5.69
fqnp359	-5.69
fqnp369	-5.69
zinc000000608382	-5.69
zinc000000895081	-5.69
zinc000019632614	-5.69
zinc75421702	-5.69
nubbe1581	-5.68
nubbe2002	-5.68
nubbe2085	-5.68
nubbe2377	-5.68
nubbe2462	-5.68
nubbe25uff	-5.68
perunpdb137	-5.68
perunpdb166	-5.68

fqnp112	-5.68
zinc000000896709	-5.68
zinc000001530752	-5.68
zinc000001530975	-5.68
zinc000001550477	-5.68
zinc000003787060	-5.68
zinc000003801163	-5.68
zinc000052716421	-5.68
nubbe1328	-5.67
nubbe1464	-5.67
nubbe1755	-5.67
nubbe2392	-5.67
nubbe290uff	-5.67
nubbe295uff	-5.67
nubbe615	-5.67
perunpdb120	-5.67
perunpdb263	-5.67
perunpdb36	-5.67
zinc000000010164	-5.67
zinc000001542113	-5.67
zinc000003929486	-5.67
zinc000007997966	-5.67
zinc000030690433	-5.67
zinc72321660	-5.67
zinc76629420	-5.67
nubbe1624	-5.66
nubbe1832	-5.66
nubbe1864	-5.66
nubbe2	-5.66
nubbe441	-5.66
nubbe750uff	-5.66
zinc000000601317	-5.66
zinc000001536779	-5.66
zinc000003831417	-5.66
zinc000006382803	-5.66
zinc000022448983	-5.66
zinc000253476027	-5.66
nubbe1180	-5.65
nubbe180uff	-5.65
nubbe385	-5.65
nubbe421uff	-5.65
perunpdb215	-5.65
zinc000000000196	-5.65

zinc000000537795	-5.65
zinc000000897408	-5.65
zinc000003938744	-5.65
zinc95993818	-5.65
nubbe1011	-5.64
nubbe2043	-5.64
nubbe267uff	-5.64
nubbe555	-5.64
zinc000000020241	-5.64
zinc000001543873	-5.64
zinc000043207851	-5.64
nubbe1006	-5.63
nubbe1307	-5.63
nubbe140uff	-5.63
nubbe1745	-5.63
nubbe1752	-5.63
nubbe2076	-5.63
nubbe243	-5.63
nubbe2520	-5.63
nubbe410uff	-5.63
nubbe472uff	-5.63
nubbe475uff	-5.63
nubbe911	-5.63
perunpdb255	-5.63
perunpdb262	-5.63
fqnp274	-5.63
zinc000000020251	-5.63
zinc000000897089	-5.63
zinc000003876068	-5.63
zinc76630238	-5.63
nubbe1129	-5.62
nubbe148	-5.62
nubbe1678	-5.62
nubbe1754	-5.62
nubbe182	-5.62
nubbe2169	-5.62
nubbe2240	-5.62
nubbe348	-5.62
nubbe385uff	-5.62
nubbe556	-5.62
perunpdb187	-5.62
fqnp19	-5.62
fqnp192	-5.62

zinc000000000746	-5.62
zinc000000011012	-5.62
zinc000001850377	-5.62
zinc000100052205	-5.62
zinc39105034	-5.62
zinc98179803	-5.62
nubbe1012	-5.61
nubbe1192	-5.61
nubbe1829	-5.61
nubbe19	-5.61
nubbe2380	-5.61
nubbe2435	-5.61
nubbe2474	-5.61
nubbe294	-5.61
nubbe472	-5.61
nubbe562uff	-5.61
nubbe614	-5.61
nubbe661	-5.61
nubbe7	-5.61
perunpdb114	-5.61
fqnp165	-5.61
fqnp20	-5.61
zinc000000001132	-5.61
zinc000000896484	-5.61
zinc000000967566	-5.61
zinc000003873295	-5.61
zinc000004097343	-5.61
zinc000038140873	-5.61
zinc000072318121	-5.61
zinc76629425	-5.61
nubbe1344	-5.6
nubbe151uff	-5.6
nubbe1787	-5.6
nubbe559uff	-5.6
nubbe588uff	-5.6
nubbe835	-5.6
perunpdb227	-5.6
fqnp41	-5.6
zinc000000596881	-5.6
zinc000000896958	-5.6
zinc000100296832	-5.6
zinc76631033	-5.6
nubbe1668	-5.59
nubbe198	-5.59

nubbe2102	-5.59
nubbe2296	-5.59
nubbe2453	-5.59
nubbe291	-5.59
nubbe333	-5.59
nubbe393uff	-5.59
nubbe400uff	-5.59
nubbe558	-5.59
nubbe657	-5.59
fqnp38	-5.59
zinc000000010163	-5.59
zinc000001530601	-5.59
zinc000007997897	-5.59
zinc000018516586	-5.59
nubbe1343	-5.58
nubbe139	-5.58
nubbe147	-5.58
nubbe1868	-5.58
nubbe1952	-5.58
nubbe2044	-5.58
nubbe2148	-5.58
nubbe2324	-5.58
nubbe310	-5.58
nubbe349	-5.58
nubbe556uff	-5.58
perunpdb25	-5.58
fqnp40	-5.58
zinc000001530912	-5.58
zinc000001999441	-5.58
zinc000003874715	-5.58
zinc000003938684	-5.58
zinc000019228902	-5.58
zinc77307084	-5.58
nubbe1222	-5.57
nubbe1651	-5.57
nubbe2004	-5.57
nubbe2013	-5.57
nubbe2133	-5.57
nubbe2335	-5.57
nubbe275uff	-5.57
nubbe336uff	-5.57
nubbe938	-5.57
perunpdb253	-5.57
fqnp47	-5.57

zinc000000020231	-5.57
zinc000000056556	-5.57
zinc000001530764	-5.57
zinc000003792417	-5.57
zinc000005844792	-5.57
zinc000068247389	-5.57
zinc000085555528	-5.57
zinc000100035385	-5.57
zinc61992278	-5.57
zinc72321641	-5.57
nubbe1021	-5.56
nubbe1763	-5.56
nubbe1859	-5.56
nubbe2307	-5.56
nubbe2349	-5.56
nubbe2458	-5.56
nubbe249uff	-5.56
nubbe344uff	-5.56
nubbe84	-5.56
nubbe876	-5.56
nubbe877	-5.56
nubbe947	-5.56
perunpdb214	-5.56
fqnp440	-5.56
fqnp461	-5.56
zinc000000009342	-5.56
zinc000001481833	-5.56
zinc000001552174	-5.56
zinc000019632670	-5.56
zinc000164760874	-5.56
nubbe2042	-5.55
nubbe238uff	-5.55
nubbe296	-5.55
nubbe566	-5.55
nubbe599uff	-5.55
nubbe658uff	-5.55
perunpdb181	-5.55
fqnp318	-5.55
zinc000000002043	-5.55
zinc000000621853	-5.55
zinc000004099009	-5.55
zinc000145808877	-5.55
nubbe1191	-5.54

nubbe1471	-5.54
nubbe1520	-5.54
nubbe2342	-5.54
nubbe2414	-5.54
nubbe597uff	-5.54
nubbe751uff	-5.54
fqnp418	-5.54
fqnp445	-5.54
zinc000019632834	-5.54
zinc000022059930	-5.54
zinc000100005670	-5.54
nubbe141uff	-5.53
nubbe2000	-5.53
nubbe2152	-5.53
nubbe375uff	-5.53
nubbe595	-5.53
nubbe671uff	-5.53
fqnp222	-5.53
zinc000000001850	-5.53
zinc000030691754	-5.53
zinc000035653009	-5.53
zinc000072320326	-5.53
zinc000094566093	-5.53
zinc000100007011	-5.53
zinc45028187	-5.53
zinc82876576	-5.53
nubbe1657	-5.52
nubbe1737	-5.52
nubbe2162	-5.52
nubbe263	-5.52
nubbe534	-5.52
nubbe673uff	-5.52
perunpdb225	-5.52
zinc000000057341	-5.52
zinc000000075008	-5.52
zinc000000622123	-5.52
zinc000001886617	-5.52
zinc000003782807	-5.52
zinc000003831531	-5.52
zinc000003913937	-5.52
zinc000016159083	-5.52
zinc96331710	-5.52
nubbe128uff	-5.51

nubbe157uff	-5.51
nubbe1874	-5.51
nubbe221	-5.51
nubbe260uff	-5.51
nubbe32uff	-5.51
nubbe358	-5.51
nubbe446	-5.51
nubbe753uff	-5.51
fqnp409	-5.51
zinc000000897322	-5.51
zinc000003920266	-5.51
zinc000034781704	-5.51
zinc000100013500	-5.51
zinc62454963	-5.51
nubbe1214	-5.5
nubbe1251	-5.5
nubbe1282	-5.5
nubbe1310	-5.5
nubbe1813	-5.5
nubbe196uff	-5.5
nubbe2205	-5.5
nubbe41	-5.5
zinc000000056647	-5.5
zinc000001531009	-5.5
zinc000003872994	-5.5
zinc39098132	-5.5
nubbe1363	-5.49
nubbe1600	-5.49
nubbe1uff	-5.49
nubbe274	-5.49
nubbe484	-5.49
nubbe659uff	-5.49
fqnp239	-5.49
fqnp32	-5.49
zinc000000538627	-5.49
zinc000004392649	-5.49
zinc000019632912	-5.49
zinc000031274852	-5.49
zinc000070466416	-5.49
zinc000100052267	-5.49
zinc62454073	-5.49
nubbe1215	-5.48
nubbe1750	-5.48
nubbe2016	-5.48
nubbe473uff	-5.48
nubbe482	-5.48
nubbe633	-5.48
nubbe671	-5.48
zinc000000000242	-5.48
zinc000000000490	-5.48
zinc000000001411	-5.48
zinc000000057435	-5.48
zinc000003873921	-5.48
zinc000004676424	-5.48
zinc000100299039	-5.48
nubbe1437	-5.47
nubbe2217	-5.47
nubbe275	-5.47
nubbe279	-5.47
perunpdb43	-5.47
fqnp188	-5.47
zinc000001849548	-5.47
zinc000003875393	-5.47
zinc000100001918	-5.47
zinc000164528615	-5.47
zinc75421703	-5.47
nubbe1381	-5.46
nubbe1663	-5.46
nubbe2058	-5.46
nubbe2398	-5.46
nubbe2473	-5.46
nubbe36	-5.46
nubbe80	-5.46
perunpdb78	-5.46
fqnp300	-5.46
zinc000000508068	-5.46
nubbe160uff	-5.45
nubbe1835	-5.45
nubbe1873	-5.45
nubbe1959	-5.45
nubbe2175	-5.45
nubbe2216	-5.45
perunpdb254	-5.45
perunpdb256	-5.45
zinc000000000373	-5.45
zinc000004097309	-5.45
zinc62454086	-5.45
zinc76608936	-5.45

nubbe1722	-5.44
nubbe1845	-5.44
nubbe2018	-5.44
nubbe2050	-5.44
nubbe2117	-5.44
nubbe241	-5.44
nubbe2475	-5.44
nubbe296uff	-5.44
nubbe29uff	-5.44
nubbe397uff	-5.44
nubbe414uff	-5.44
nubbe432	-5.44
nubbe5uff	-5.44
fqnp197	-5.44
fqnp344	-5.44
zinc000000537928	-5.44
zinc000000968301	-5.44
zinc000001530940	-5.44
zinc000100024116	-5.44
zinc75421765	-5.44
zinc76637435	-5.44
zinc77307085	-5.44
nubbe1091	-5.43
nubbe1421	-5.43
nubbe162	-5.43
nubbe1666	-5.43
nubbe1847	-5.43
nubbe188uff	-5.43
nubbe2031	-5.43
nubbe457	-5.43
nubbe673	-5.43
fqnp257	-5.43
zinc000000001382	-5.43
zinc000000537964	-5.43
zinc000001530617	-5.43
zinc000001530621	-5.43
zinc000001530814	-5.43
zinc000096272772	-5.43
zinc000100009278	-5.43
zinc000245204924	-5.43
zinc82855711	-5.43
nubbe1646	-5.42
nubbe582	-5.42

nubbe969	-5.42
zinc000000000301	-5.42
zinc000000643055	-5.42
zinc000012358719	-5.42
zinc000053045054	-5.42
nubbe1696	-5.41
nubbe1760	-5.41
nubbe1857	-5.41
nubbe2083	-5.41
nubbe2208	-5.41
nubbe2482	-5.41
nubbe289uff	-5.41
nubbe557	-5.41
nubbe643	-5.41
perunpdb159	-5.41
fqnp166	-5.41
zinc000000005823	-5.41
zinc000000057464	-5.41
zinc000001530695	-5.41
zinc000003938652	-5.41
zinc000013986658	-5.41
zinc95993816	-5.41
nubbe1751	-5.4
nubbe253	-5.4
nubbe254	-5.4
nubbe59uff	-5.4
nubbe655	-5.4
perunpdb277	-5.4
fqnp168	-5.4
zinc000000057533	-5.4
zinc000000968273	-5.4
zinc000003812865	-5.4
zinc000100036760	-5.4
zinc95993815	-5.4
nubbe1220	-5.39
nubbe1242	-5.39
nubbe1740	-5.39
nubbe1825	-5.39
nubbe1902	-5.39
nubbe35uff	-5.39
nubbe408	-5.39
nubbe486uff	-5.39
nubbe540	-5.39

nubbe736uff	-5.39
perunpdb179	-5.39
zinc000000897244	-5.39
zinc000003831477	-5.39
zinc000003938704	-5.39
zinc000013831130	-5.39
zinc000013912394	-5.39
nubbe1065	-5.38
nubbe1216	-5.38
nubbe1518	-5.38
nubbe1743	-5.38
nubbe1892	-5.38
nubbe1897	-5.38
nubbe2022	-5.38
nubbe289	-5.38
nubbe352uff	-5.38
fqnp279	-5.38
zinc000000007782	-5.38
zinc000000896968	-5.38
zinc000003990451	-5.38
nubbe1439	-5.37
nubbe1637	-5.37
nubbe1721	-5.37
nubbe1914	-5.37
nubbe2007	-5.37
nubbe254uff	-5.37
nubbe308uff	-5.37
nubbe333uff	-5.37
nubbe371	-5.37
nubbe669uff	-5.37
nubbe746uff	-5.37
fqnp400	-5.37
zinc000019796080	-5.37
zinc000100009280	-5.37
zinc000100037855	-5.37
zinc76630243	-5.37
nubbe1226	-5.36
nubbe157	-5.36
nubbe1638	-5.36
nubbe1738	-5.36
nubbe1984	-5.36
nubbe2029	-5.36
nubbe251	-5.36
nubbe357	-5.36

perunpdb30	-5.36
perunpdb58	-5.36
zinc000000001554	-5.36
zinc000001550766	-5.36
zinc000003830441	-5.36
zinc000018115268	-5.36
nubbe1155	-5.35
nubbe2038	-5.35
nubbe2258	-5.35
nubbe476uff	-5.35
nubbe654	-5.35
perunpdb226	-5.35
fqnp130	-5.35
fqnp395	-5.35
zinc000002000707	-5.35
zinc000003976838	-5.35
zinc000060325170	-5.35
nubbe1024	-5.34
nubbe1692	-5.34
nubbe1741	-5.34
nubbe218uff	-5.34
nubbe476	-5.34
nubbe583uff	-5.34
nubbe594	-5.34
nubbe657uff	-5.34
nubbe741uff	-5.34
nubbe870	-5.34
perunpdb213	-5.34
fqnp290	-5.34
zinc000000538266	-5.34
zinc000000897291	-5.34
zinc000001850376	-5.34
zinc000002522648	-5.34
zinc000002522669	-5.34
zinc000003830339	-5.34
zinc000242548690	-5.34
nubbe1010	-5.33
nubbe1342	-5.33
nubbe14uff	-5.33
nubbe175uff	-5.33
nubbe2057	-5.33
nubbe23	-5.33
nubbe377uff	-5.33
nubbe485uff	-5.33

fqnp243	-5.33
fqnp7	-5.33
zinc000002015928	-5.33
zinc000002561203	-5.33
zinc000003873371	-5.33
nubbe188	-5.32
nubbe2021	-5.32
nubbe2179	-5.32
nubbe237	-5.32
nubbe269	-5.32
nubbe447uff	-5.32
fqnp160	-5.32
zinc000095616603	-5.32
zinc76599676	-5.32
nubbe1860	-5.31
nubbe2422	-5.31
nubbe35	-5.31
nubbe353uff	-5.31
perunpdb265	-5.31
zinc000002016037	-5.31
nubbe1746	-5.3
nubbe2150	-5.3
fqnp167	-5.3
zinc000001482164	-5.3
zinc000004641374	-5.3
zinc000008403947	-5.3
zinc000019632633	-5.3
zinc000100015775	-5.3
zinc000169621231	-5.3
zinc75601818	-5.3
nubbe1304	-5.29
nubbe1444	-5.29
nubbe145uff	-5.29
nubbe1521	-5.29
nubbe1580	-5.29
nubbe2027	-5.29
nubbe2128	-5.29
nubbe949	-5.29
nubbe970	-5.29
perunpdb4	-5.29
zinc000001530769	-5.29
zinc000004099008	-5.29
zinc000027428713	-5.29

nubbe1114	-5.28
nubbe1333	-5.28
zinc000000896740	-5.28
zinc000000968303	-5.28
zinc000003874185	-5.28
zinc000022010649	-5.28
zinc000038197764	-5.28
zinc000053683345	-5.28
zinc000253476025	-5.28
zinc76631020	-5.28
nubbe1109	-5.27
nubbe175	-5.27
nubbe2032	-5.27
nubbe209uff	-5.27
nubbe406uff	-5.27
nubbe43uff	-5.27
nubbe68	-5.27
fqnp198	-5.27
fqnp240	-5.27
zinc000017285869	-5.27
nubbe1620	-5.26
nubbe1781	-5.26
nubbe2047	-5.26
nubbe229uff	-5.26
nubbe2512	-5.26
nubbe373uff	-5.26
nubbe658	-5.26
perunpdb142	-5.26
zinc000000001464	-5.26
zinc000000896455	-5.26
zinc000001530641	-5.26
zinc000003873160	-5.26
zinc000003918087	-5.26
zinc000019632628	-5.26
zinc000036294079	-5.26
nubbe1895	-5.25
nubbe2370	-5.25
nubbe2371	-5.25
perunpdb160	-5.25
zinc000000000061	-5.25
zinc000000006016	-5.25
zinc000003873936	-5.25
zinc000253917094	-5.25

nubbe135uff	-5.24
nubbe1837	-5.24
nubbe1901	-5.24
nubbe2132	-5.24
nubbe2466	-5.24
nubbe41uff	-5.24
nubbe70	-5.24
perunpdb34	-5.24
zinc000003872566	-5.24
zinc000019364230	-5.24
zinc000084758235	-5.24
nubbe1143	-5.23
nubbe2015	-5.23
nubbe253uff	-5.23
nubbe279uff	-5.23
nubbe599	-5.23
nubbe616	-5.23
nubbe628	-5.23
nubbe64	-5.23
fqnp3	-5.23
zinc000000004319	-5.23
zinc000000896663	-5.23
zinc000000968330	-5.23
zinc000004228258	-5.23
zinc000005179119	-5.23
zinc000016929327	-5.23
zinc000019796168	-5.23
nubbe113	-5.22
nubbe121uff	-5.22
nubbe130	-5.22
nubbe1367	-5.22
nubbe1557	-5.22
nubbe18uff	-5.22
nubbe297	-5.22
nubbe654uff	-5.22
nubbe735uff	-5.22
perunpdb38	-5.22
fqnp127	-5.22
fqnp150	-5.22
zinc000000057532	-5.22
zinc000000113382	-5.22
zinc000000897385	-5.22
zinc000001530611	-5.22
zinc000002005550	-5.22

zinc62454081	-5.22
nubbe155uff	-5.21
nubbe250uff	-5.21
nubbe69uff	-5.21
nubbe984	-5.21
fqnp378	-5.21
zinc000000057512	-5.21
zinc000000967597	-5.21
zinc000003812869	-5.21
zinc000030691727	-5.21
zinc39105186	-5.21
zinc65454683	-5.21
nubbe1200	-5.2
nubbe1284	-5.2
nubbe1616	-5.2
nubbe2111	-5.2
nubbe2328	-5.2
nubbe431	-5.2
nubbe44	-5.2
nubbe449	-5.2
nubbe624uff	-5.2
nubbe925	-5.2
fqnp125	-5.2
fqnp397	-5.2
fqnp59	-5.2
zinc000000001644	-5.2
zinc000000537752	-5.2
zinc000004212945	-5.2
zinc000018324776	-5.2
zinc000100036955	-5.2
zinc75627227	-5.2
nubbe1017	-5.19
nubbe1701	-5.19
nubbe2094	-5.19
nubbe2130	-5.19
nubbe378	-5.19
nubbe45	-5.19
zinc000003812841	-5.19
zinc000003871923	-5.19
zinc000003882036	-5.19
zinc39058139	-5.19
nubbe1443	-5.18
nubbe148uff	-5.18
nubbe1865	-5.18

nubbe2028	-5.18
nubbe359	-5.18
nubbe412	-5.18
nubbe475	-5.18
nubbe536	-5.18
fqnp104	-5.18
zinc000000000565	-5.18
zinc000000154964	-5.18
zinc000003830716	-5.18
zinc000022059926	-5.18
zinc16138079	-5.18
zinc82855713	-5.18
nubbe1618	-5.17
nubbe336	-5.17
nubbe4	-5.17
nubbe594uff	-5.17
fqnp442	-5.17
zinc000000602128	-5.17
zinc000003830405	-5.17
zinc000085534098	-5.17
zinc000095616601	-5.17
zinc76608933	-5.17
nubbe1068	-5.16
nubbe2138	-5.16
nubbe2147	-5.16
nubbe2297	-5.16
nubbe237uff	-5.16
nubbe2386	-5.16
nubbe2417	-5.16
nubbe241uff	-5.16
nubbe446uff	-5.16
nubbe53uff	-5.16
nubbe555uff	-5.16
nubbe601uff	-5.16
zinc000000001798	-5.16
zinc000003922770	-5.16
zinc000053045055	-5.16
zinc39097474	-5.16
nubbe1169	-5.15
nubbe1839	-5.15
nubbe1983	-5.15
nubbe1999	-5.15
nubbe346	-5.15

nubbe484uff	-5.15
nubbe543	-5.15
nubbe656uff	-5.15
fqnp394	-5.15
fqnp447	-5.15
zinc000001530806	-5.15
zinc000022448696	-5.15
zinc000035465976	-5.15
zinc000087496429	-5.15
zinc62454992	-5.15
nubbe1174	-5.14
nubbe1250	-5.14
nubbe197	-5.14
nubbe2467	-5.14
nubbe356	-5.14
nubbe537uff	-5.14
nubbe733uff	-5.14
nubbe825uff	-5.14
perunpdb186	-5.14
fqnp156	-5.14
fqnp31	-5.14
zinc000000001281	-5.14
zinc000000008492	-5.14
zinc000000016154	-5.14
zinc000000057253	-5.14
zinc000000137884	-5.14
zinc000000968336	-5.14
zinc000003813042	-5.14
zinc000003872520	-5.14
zinc000006021033	-5.14
zinc000008855117	-5.14
nubbe1601	-5.13
nubbe1627	-5.13
nubbe1747	-5.13
nubbe1817	-5.13
nubbe2041	-5.13
nubbe224uff	-5.13
nubbe441uff	-5.13
zinc000000002009	-5.13
zinc000000004949	-5.13
zinc000001482197	-5.13
zinc000001530689	-5.13
zinc000003812862	-5.13

zinc000003830569	-5.13
zinc000004074875	-5.13
zinc000095616599	-5.13
nubbe128	-5.12
nubbe137	-5.12
nubbe2206	-5.12
perunpdb177	-5.12
zinc000003926298	-5.12
zinc000011681563	-5.12
zinc000169621220	-5.12
zinc39059648	-5.12
zinc82854740	-5.12
nubbe129uff	-5.11
nubbe2379	-5.11
perunpdb221	-5.11
perunpdb24	-5.11
fqnp398	-5.11
zinc000003794601	-5.11
zinc15768131	-5.11
zinc62454096	-5.11
nubbe1369	-5.1
nubbe183	-5.1
nubbe42	-5.1
nubbe580	-5.1
zinc000000002101	-5.1
zinc000000896463	-5.1
zinc000001530710	-5.1
zinc000005844788	-5.1
nubbe1823	-5.09
nubbe1906	-5.09
nubbe2254	-5.09
nubbe227	-5.09
fqnp8	-5.09
zinc000000001735	-5.09
zinc000000895199	-5.09
zinc000003914596	-5.09
zinc000003916214	-5.09
zinc000004258316	-5.09
zinc000004468780	-5.09
zinc000013585233	-5.09
zinc000018043251	-5.09
nubbe135	-5.08
nubbe195uff	-5.08
nubbe354uff	-5.08

nubbe69	-5.08
fqnp18	-5.08
zinc000000057624	-5.08
zinc42227681	-5.08
nubbe100uff	-5.07
nubbe1254	-5.07
nubbe136	-5.07
nubbe2136	-5.07
nubbe298	-5.07
nubbe3uff	-5.07
nubbe89	-5.07
fqnp436	-5.07
zinc000001530973	-5.07
zinc000002525885	-5.07
zinc000003807804	-5.07
zinc000003813061	-5.07
zinc000004213946	-5.07
zinc000004474460	-5.07
zinc000019632668	-5.07
zinc76598893	-5.07
nubbe1221	-5.06
nubbe166	-5.06
nubbe1819	-5.06
nubbe1992	-5.06
nubbe2141	-5.06
nubbe2331	-5.06
nubbe2390	-5.06
nubbe2433	-5.06
nubbe304	-5.06
nubbe368uff	-5.06
perunpdb219	-5.06
fqnp132	-5.06
fqnp453	-5.06
fqnp58	-5.06
zinc000000057513	-5.06
zinc000001530756	-5.06
zinc000100036756	-5.06
zinc76630229	-5.06
zinc76637429	-5.06
zinc98179804	-5.06
nubbe2075	-5.05
nubbe2103	-5.05
nubbe474	-5.05
nubbe558uff	-5.05

nubbe631	-5.05
nubbe976	-5.05
perunpdb178	-5.05
perunpdb42	-5.05
zinc000000004166	-5.05
zinc000001530951	-5.05
zinc000001850374	-5.05
zinc000003819138	-5.05
zinc000003831474	-5.05
zinc000004098633	-5.05
zinc000068204830	-5.05
zinc000169677008	-5.05
zinc21792937	-5.05
nubbe1115	-5.04
nubbe1625	-5.04
nubbe1753	-5.04
nubbe1789	-5.04
nubbe19uff	-5.04
nubbe225uff	-5.04
zinc000000039089	-5.04
zinc000001530751	-5.04
nubbe1869	-5.03
nubbe534uff	-5.03
nubbe754uff	-5.03
perunpdb193	-5.03
fqnp384	-5.03
zinc000000057206	-5.03
zinc000008466459	-5.03
zinc000030691760	-5.03
nubbe108uff	-5.02
nubbe121	-5.02
nubbe203	-5.02
nubbe244uff	-5.02
nubbe637	-5.02
nubbe9	-5.02
nubbe9uff	-5.02
zinc000000002273	-5.02
zinc000001530805	-5.02
zinc000003986735	-5.02
zinc000013550868	-5.02
zinc35055762	-5.02
zinc76599718	-5.02
zinc76637431	-5.02

nubbe2092	-5.01
nubbe521	-5.01
nubbe61uff	-5.01
perunpdb158	-5.01
zinc000000020244	-5.01
zinc000001530716	-5.01
zinc000001530788	-5.01
zinc000001639567	-5.01
zinc000003831006	-5.01
zinc000003831332	-5.01
zinc000004340269	-5.01
zinc000019632713	-5.01
zinc000026011099	-5.01
nubbe1259	-5
nubbe1779	-5
zinc000003781664	-5
zinc000022056030	-5
zinc75601819	-5
nubbe1312	-4.99
nubbe1838	-4.99
nubbe2060	-4.99
nubbe367uff	-4.99
nubbe627uff	-4.99
nubbe910	-4.99
fqnp10	-4.99
fqnp315	-4.99
zinc000043195697	-4.99
nubbe1217	-4.98
nubbe42uff	-4.98
nubbe65	-4.98
nubbe863	-4.98
fqnp261	-4.98
zinc000000607986	-4.98
zinc000002019954	-4.98
zinc000002020233	-4.98
zinc000003802690	-4.98
zinc000035801098	-4.98
zinc000203686879	-4.98
zinc76598755	-4.98
zinc91756065	-4.98
nubbe1480	-4.97
nubbe1631	-4.97
nubbe1879	-4.97

nubbe2476	-4.97
nubbe392	-4.97
nubbe455	-4.97
fqnp99	-4.97
zinc000000968345	-4.97
zinc000003827556	-4.97
zinc000012503151	-4.97
zinc000014879972	-4.97
zinc000019144226	-4.97
zinc000084758479	-4.97
zinc000252286875	-4.97
zinc39059646	-4.97
zinc39097476	-4.97
nubbe134	-4.96
nubbe15	-4.96
nubbe1764	-4.96
nubbe2218	-4.96
nubbe403uff	-4.96
nubbe473	-4.96
nubbe867	-4.96
zinc000001571009	-4.96
zinc000035342787	-4.96
zinc000100015780	-4.96
zinc76608943	-4.96
zinc82855712	-4.96
nubbe1108	-4.95
nubbe1289	-4.95
nubbe1308	-4.95
nubbe1921	-4.95
nubbe342uff	-4.95
nubbe52uff	-4.95
nubbe84uff	-4.95
perunpdb20	-4.95
zinc000000537791	-4.95
zinc000003798763	-4.95
zinc000003833846	-4.95
zinc76599666	-4.95
nubbe1015	-4.94
nubbe113uff	-4.94
nubbe1288	-4.94
nubbe2048	-4.94
nubbe2081	-4.94
nubbe2347	-4.94
nubbe630	-4.94

nubbe908	-4.94
perunpdb51	-4.94
fqnp61	-4.94
zinc000000056653	-4.94
zinc000003802417	-4.94
zinc000003874950	-4.94
zinc000028232750	-4.94
zinc000030691763	-4.94
zinc000100071256	-4.94
zinc39059658	-4.94
zinc39060222	-4.94
zinc82854738	-4.94
nubbe153uff	-4.93
nubbe323	-4.93
perunpdb196	-4.93
fqnp389	-4.93
zinc000000538386	-4.93
zinc000003607120	-4.93
zinc000035465980	-4.93
zinc000096942202	-4.93
nubbe134uff	-4.92
nubbe2350	-4.92
nubbe300	-4.92
perunpdb220	-4.92
fqnp195	-4.92
fqnp468	-4.92
zinc000000020783	-4.92
zinc000001530555	-4.92
zinc000001530694	-4.92
zinc000003008621	-4.92
zinc000003800706	-4.92
zinc000028639340	-4.92
nubbe1761	-4.91
nubbe2036	-4.91
nubbe2139	-4.91
nubbe2140	-4.91
nubbe354	-4.91
nubbe595uff	-4.91
fqnp100	-4.91
zinc000000105196	-4.91
zinc000000592419	-4.91
zinc000002539827	-4.91
zinc000257553304	-4.91
zinc34451127	-4.91

nubbe1432	-4.9
nubbe20uff	-4.9
nubbe2330	-4.9
nubbe627	-4.9
fqnp124	-4.9
zinc000000057062	-4.9
zinc000000591993	-4.9
zinc000001547851	-4.9
zinc000001843047	-4.9
zinc39059660	-4.9
nubbe101uff	-4.89
nubbe1552	-4.89
nubbe1977	-4.89
nubbe248	-4.89
nubbe367	-4.89
nubbe412uff	-4.89
nubbe425	-4.89
nubbe449uff	-4.89
nubbe868	-4.89
zinc000000388462	-4.89
zinc000000391812	-4.89
zinc000000608101	-4.89
zinc000000896698	-4.89
zinc000003651680	-4.89
zinc000026664090	-4.89
zinc04082733	-4.89
zinc39105188	-4.89
zinc49934104	-4.89
nubbe154	-4.88
nubbe261uff	-4.88
nubbe357uff	-4.88
nubbe55	-4.88
nubbe593uff	-4.88
fqnp174	-4.88
zinc000000000607	-4.88
zinc000000012346	-4.88
zinc000000156395	-4.88
zinc000019364229	-4.88
zinc35567004	-4.88
zinc76598887	-4.88
zinc82846296	-4.88
nubbe1858	-4.87
nubbe1871	-4.87

nubbe2112	-4.87
nubbe31	-4.87
nubbe40uff	-4.87
nubbe630uff	-4.87
zinc000000001899	-4.87
zinc000003830449	-4.87
zinc000004038341	-4.87
zinc000008143866	-4.87
zinc000012360535	-4.87
zinc000014210642	-4.87
zinc000014879975	-4.87
zinc000022443609	-4.87
zinc34060545	-4.87
zinc45028188	-4.87
zinc75601817	-4.87
zinc76630233	-4.87
nubbe1309	-4.86
nubbe2039	-4.86
nubbe2278	-4.86
nubbe4uff	-4.86
nubbe53	-4.86
nubbe624	-4.86
perunpdb224	-4.86
fqnp17	-4.86
fqnp441	-4.86
zinc000000000416	-4.86
zinc000000607939	-4.86
zinc000000896666	-4.86
zinc000001842633	-4.86
zinc000014879992	-4.86
zinc76598889	-4.86
nubbe557uff	-4.85
perunpdb198	-4.85
zinc000000643143	-4.85
zinc000001530638	-4.85
zinc000003812851	-4.85
zinc000004097344	-4.85
zinc000008214619	-4.85
nubbe1389	-4.84
nubbe2183	-4.84
nubbe59	-4.84
nubbe8uff	-4.84
perunpdb161	-4.84

fqnp12	-4.84
fqnp173	-4.84
zinc000000001758	-4.84
zinc000000136138	-4.84
zinc000000538483	-4.84
zinc000003776970	-4.84
zinc000003830212	-4.84
zinc21792941	-4.84
zinc35055761	-4.84
zinc82846298	-4.84
zinc91847710	-4.84
nubbe1889	-4.83
nubbe2011	-4.83
nubbe734uff	-4.83
fqnp13	-4.83
zinc000000001341	-4.83
zinc000000020240	-4.83
zinc000001530922	-4.83
zinc000001534965	-4.83
zinc000003927870	-4.83
zinc76631029	-4.83
zinc82846284	-4.83
nubbe138	-4.82
nubbe1816	-4.82
nubbe426uff	-4.82
nubbe440	-4.82
nubbe757uff	-4.82
nubbe914	-4.82
fqnp45	-4.82
zinc000000001011	-4.82
zinc000000020237	-4.82
zinc000003830347	-4.82
zinc000018279854	-4.82
zinc000095619101	-4.82
zinc38234352	-4.82
nubbe1506	-4.81
nubbe1916	-4.81
zinc000000015515	-4.81
zinc000000643138	-4.81
zinc000000968328	-4.81
zinc000001576892	-4.81
zinc000001633889	-4.81
zinc000096006023	-4.81
zinc76599721	-4.81

nubbe1881	-4.8
nubbe1888	-4.8
nubbe1978	-4.8
nubbe2178	-4.8
nubbe478uff	-4.8
nubbe538	-4.8
nubbe734	-4.8
perunpdb182	-4.8
zinc000004474443	-4.8
zinc000253387843	-4.8
zinc35151524	-4.8
zinc39116167	-4.8
zinc42305217	-4.8
zinc42469141	-4.8
zinc42469144	-4.8
zinc76599715	-4.8
nubbe1664	-4.79
nubbe755uff	-4.79
nubbe850	-4.79
nubbe99uff	-4.79
fqnp262	-4.79
zinc000000000693	-4.79
zinc000003589203	-4.79
zinc000118913562	-4.79
zinc82384239	-4.79
nubbe1127	-4.78
nubbe1894	-4.78
nubbe235	-4.78
nubbe450uff	-4.78
fqnp140	-4.78
zinc000000538283	-4.78
zinc000001690604	-4.78
zinc000004215234	-4.78
zinc000034636383	-4.78
zinc76608939	-4.78
nubbe1300	-4.77
nubbe1875	-4.77
nubbe277	-4.77
nubbe43	-4.77
nubbe931	-4.77
fqnp9	-4.77
zinc000000001317	-4.77
zinc000000004028	-4.77
zinc000000056568	-4.77

zinc000000057001	-4.77
zinc000000085733	-4.77
zinc000000113355	-4.77
zinc000001530637	-4.77
zinc000012495062	-4.77
zinc000043100953	-4.77
zinc06185614	-4.77
zinc39116169	-4.77
zinc42469146	-4.77
zinc75627232	-4.77
zinc76591233	-4.77
zinc76598891	-4.77
nubbe1409	-4.76
nubbe1790	-4.76
nubbe2129	-4.76
nubbe762uff	-4.76
zinc000000001795	-4.76
zinc000000895154	-4.76
zinc000004577910	-4.76
zinc000169621230	-4.76
zinc33961860	-4.76
nubbe1029	-4.75
nubbe1380	-4.75
nubbe2222	-4.75
nubbe58uff	-4.75
fqnp136	-4.75
zinc000008220909	-4.75
zinc000021297660	-4.75
zinc06562443	-4.75
nubbe2137	-4.74
nubbe2439	-4.74
nubbe95uff	-4.74
perunpdb185	-4.74
fqnp44	-4.74
fqnp457	-4.74
zinc000000000471	-4.74
zinc000000007673	-4.74
zinc000000897085	-4.74
zinc000001530816	-4.74
zinc000003876069	-4.74
zinc000003989268	-4.74
zinc000006827695	-4.74
zinc82391766	-4.74

nubbe1848	-4.73
nubbe1877	-4.73
nubbe2182	-4.73
nubbe229	-4.73
nubbe2318	-4.73
nubbe747uff	-4.73
perunpdb57	-4.73
fqnp225	-4.73
fqnp322	-4.73
zinc000000020259	-4.73
zinc000000538065	-4.73
zinc000000968305	-4.73
zinc000006845963	-4.73
zinc16952677	-4.73
zinc72321658	-4.73
nubbe1018	-4.72
nubbe1687	-4.72
nubbe1841	-4.72
nubbe1861	-4.72
nubbe1862	-4.72
nubbe20	-4.72
nubbe309	-4.72
nubbe70uff	-4.72
fqnp155	-4.72
zinc000000537805	-4.72
zinc000019166988	-4.72
zinc35298583	-4.72
zinc82384238	-4.72
nubbe1297	-4.71
nubbe31uff	-4.71
fqnp11	-4.71
fqnp462	-4.71
zinc000000119344	-4.71
zinc000001530862	-4.71
zinc000002005305	-4.71
zinc000003831151	-4.71
zinc000169289767	-4.71
zinc82854737	-4.71
nubbe1030	-4.7
nubbe1126	-4.7
nubbe1153	-4.7
nubbe413uff	-4.7
fqnp101	-4.7

fqnp129	-4.7
fqnp50	-4.7
zinc000000596731	-4.7
zinc000013597823	-4.7
zinc000252286878	-4.7
zinc62454589	-4.7
zinc71789633	-4.7
zinc82393646	-4.7
nubbe1619	-4.69
nubbe2118	-4.69
nubbe2127	-4.69
nubbe265	-4.69
nubbe342	-4.69
nubbe474uff	-4.69
nubbe668uff	-4.69
fqnp393	-4.69
fqnp62	-4.69
zinc000000000053	-4.69
zinc000001552908	-4.69
zinc000012404516	-4.69
zinc000098023177	-4.69
zinc35151526	-4.69
zinc42227695	-4.69
nubbe1082	-4.68
nubbe372	-4.68
nubbe478	-4.68
nubbe668	-4.68
zinc000000001982	-4.68
zinc000000895360	-4.68
zinc000006021043	-4.68
nubbe2480	-4.67
nubbe26uff	-4.67
nubbe3	-4.67
nubbe745uff	-4.67
fqnp161	-4.67
fqnp373	-4.67
fqnp406	-4.67
fqnp407	-4.67
fqnp6	-4.67
zinc000000001084	-4.67
zinc000000006481	-4.67
zinc000000057254	-4.67
zinc000001482094	-4.67
zinc00901326	-4.67

zinc32227795	-4.67
nubbe127	-4.66
nubbe1374	-4.66
nubbe2309	-4.66
nubbe242	-4.66
nubbe405uff	-4.66
nubbe414	-4.66
nubbe655uff	-4.66
perunpdb223	-4.66
perunpdb44	-4.66
zinc000000020257	-4.66
zinc000006036847	-4.66
nubbe1019	-4.65
nubbe162uff	-4.65
nubbe40	-4.65
perunpdb276	-4.65
perunpdb280	-4.65
fqnp126	-4.65
fqnp260	-4.65
fqnp396	-4.65
zinc000000395010	-4.65
zinc000001996784	-4.65
zinc16953164	-4.65
zinc62454989	-4.65
nubbe1154	-4.64
nubbe155	-4.64
nubbe232uff	-4.64
nubbe371uff	-4.64
nubbe408uff	-4.64
nubbe568	-4.64
perunpdb176	-4.64
zinc000000004413	-4.64
zinc000001530974	-4.64
zinc000003813010	-4.64
zinc000003979899	-4.64
zinc01081506	-4.64
zinc01612537	-4.64
zinc32227798	-4.64
zinc37490676	-4.64
zinc76591241	-4.64
zinc82855714	-4.64
nubbe2207	-4.63
nubbe349uff	-4.63
zinc000001536109	-4.63

zinc000001554588	-4.63
zinc000006627681	-4.63
zinc51887125	-4.63
zinc76592639	-4.63
zinc76592642	-4.63
zinc76599727	-4.63
zinc82393087	-4.63
nubbe1671	-4.62
nubbe2091	-4.62
nubbe290	-4.62
zinc34506019	-4.62
zinc82399749	-4.62
nubbe1773	-4.61
nubbe2104	-4.61
nubbe260	-4.61
zinc000000895302	-4.61
zinc000001482184	-4.61
zinc000001853205	-4.61
zinc000003816514	-4.61
zinc000003953037	-4.61
nubbe136uff	-4.6
nubbe1680	-4.6
nubbe628uff	-4.6
nubbe653uff	-4.6
zinc000003861768	-4.6
zinc000004468778	-4.6
zinc000012402836	-4.6
zinc000028232755	-4.6
zinc65743304	-4.6
nubbe195	-4.59
nubbe2185	-4.59
nubbe62	-4.59
nubbe626	-4.59
nubbe939	-4.59
perunpdb222	-4.59
zinc000019144231	-4.59
zinc50916527	-4.59
nubbe2126	-4.58
nubbe2469	-4.58
nubbe417uff	-4.58
nubbe477uff	-4.58
nubbe482uff	-4.58
nubbe631uff	-4.58

fqnp313	-4.58
zinc000000001931	-4.58
zinc000000020255	-4.58
zinc000000074836	-4.58
zinc000001035331	-4.58
nubbe1655	-4.57
nubbe634uff	-4.57
fqnp433	-4.57
zinc000000020230	-4.57
zinc000003798537	-4.57
zinc06185631	-4.57
zinc39116114	-4.57
nubbe1311	-4.56
nubbe238	-4.56
nubbe26	-4.56
nubbe492	-4.56
nubbe536uff	-4.56
nubbe544	-4.56
perunpdb48	-4.56
fqnp66	-4.56
zinc000000113442	-4.56
zinc000001530707	-4.56
zinc000003645145	-4.56
zinc02379604	-4.56
zinc38234064	-4.56
zinc40734066	-4.56
nubbe1385	-4.55
nubbe1482	-4.55
nubbe1504	-4.55
nubbe1595	-4.55
nubbe1670	-4.55
nubbe1907	-4.55
zinc000000005878	-4.55
zinc000026985532	-4.55
zinc000043207237	-4.55
zinc000100070937	-4.55
zinc35649799	-4.55
nubbe142uff	-4.54
nubbe161uff	-4.54
nubbe1885	-4.54
nubbe1886	-4.54
nubbe2343	-4.54
nubbe2470	-4.54

nubbe537	-4.54
nubbe638	-4.54
perunpdb150	-4.54
fqnp469	-4.54
zinc000000895457	-4.54
zinc000001530817	-4.54
zinc000003779042	-4.54
zinc000003823492	-4.54
nubbe15uff	-4.53
nubbe1900	-4.53
nubbe640	-4.53
perunpdb95	-4.53
zinc000013545636	-4.53
zinc05063918	-4.53
zinc39103505	-4.53
nubbe1287	-4.52
nubbe2166	-4.52
nubbe403	-4.52
nubbe578uff	-4.52
perunpdb55	-4.52
fqnp438	-4.52
zinc000000901791	-4.52
zinc000003843198	-4.52
zinc000004978673	-4.52
zinc000005133329	-4.52
zinc000013298313	-4.52
zinc39100730	-4.52
nubbe1495	-4.51
nubbe16	-4.51
nubbe265uff	-4.51
nubbe27uff	-4.51
nubbe442	-4.51
nubbe765uff	-4.51
fqnp196	-4.51
zinc000000005895	-4.51
zinc000019796087	-4.51
zinc000022059268	-4.51
zinc36460092	-4.51
zinc48857967	-4.51
zinc78916384	-4.51
nubbe1685	-4.5
perunpdb7	-4.5
zinc000000014360	-4.5
zinc000000056652	-4.5

zinc000000897258	-4.5
zinc000003977764	-4.5
zinc000019364222	-4.5
zinc000019364224	-4.5
zinc000096942201	-4.5
zinc000100008275	-4.5
zinc16138077	-4.5
nubbe1170	-4.49
nubbe1882	-4.49
nubbe196	-4.49
nubbe1994	-4.49
perunpdb266	-4.49
fqnp437	-4.49
fqnp54	-4.49
zinc000000403010	-4.49
zinc000001530701	-4.49
zinc000001530703	-4.49
zinc45028190	-4.49
nubbe138uff	-4.48
nubbe2122	-4.48
nubbe653	-4.48
nubbe86uff	-4.48
zinc000100003902	-4.48
zinc12504453	-4.48
zinc36436848	-4.48
zinc51887124	-4.48
nubbe209	-4.47
nubbe2463	-4.47
nubbe763uff	-4.47
nubbe932	-4.47
zinc000000057340	-4.47
zinc000009212427	-4.47
zinc34485150	-4.47
nubbe1152	-4.46
nubbe119	-4.46
nubbe1382	-4.46
nubbe2134	-4.46
nubbe559	-4.46
zinc000000002055	-4.46
zinc000035465978	-4.46
zinc000087515509	-4.46
zinc75421740	-4.46
zinc76632281	-4.46
nubbe1594	-4.45

nubbe2097	-4.45
nubbe86	-4.45
perunpdb113	-4.45
fqnp375	-4.45
zinc000000001408	-4.45
zinc000000056645	-4.45
zinc000000895032	-4.45
zinc000001481910	-4.45
zinc000003871967	-4.45
zinc82854739	-4.45
zinc96327097	-4.45
nubbe1298	-4.44
nubbe2115	-4.44
nubbe2319	-4.44
nubbe55uff	-4.44
fqnp157	-4.44
zinc40734069	-4.44
zinc42432370	-4.44
zinc76591243	-4.44
nubbe239uff	-4.43
nubbe2456	-4.43
fqnp267	-4.43
fqnp391	-4.43
zinc000000000506	-4.43
zinc000000001688	-4.43
zinc000096006020	-4.43
zinc39058097	-4.43
zinc42207550	-4.43
zinc76593292	-4.43
nubbe1202	-4.42
nubbe2119	-4.42
nubbe362uff	-4.42
perunpdb11	-4.42
perunpdb16	-4.42
perunpdb45	-4.42
zinc000003629271	-4.42
zinc05492902	-4.42
nubbe1261	-4.41
nubbe1744	-4.41
nubbe1867	-4.41
nubbe57	-4.41
nubbe64uff	-4.41
nubbe680	-4.41

zinc000000034157	-4.41
zinc000003806104	-4.41
zinc03875042	-4.41
zinc43419395	-4.41
zinc76593308	-4.41
nubbe300uff	-4.4
nubbe334uff	-4.4
nubbe391	-4.4
fqnp266	-4.4
fqnp424	-4.4
zinc000000000922	-4.4
zinc000000002028	-4.4
zinc000000155269	-4.4
zinc000001530950	-4.4
zinc000002847375	-4.4
zinc000019144216	-4.4
zinc000252286877	-4.4
zinc04098893	-4.4
zinc35567008	-4.4
zinc39058138	-4.4
zinc76592629	-4.4
nubbe235uff	-4.39
nubbe284uff	-4.39
nubbe968	-4.39
fqnp56	-4.39
zinc000000056646	-4.39
zinc000001530570	-4.39
zinc000003817234	-4.39
zinc000003830264	-4.39
zinc000003830990	-4.39
perunpdb245	-4.38
fqnp88	-4.38
zinc000000000215	-4.38
zinc000000006226	-4.38
zinc000008101109	-4.38
zinc37490674	-4.38
nubbe1283	-4.37
nubbe277uff	-4.37
nubbe343uff	-4.37
nubbe397	-4.37
nubbe424uff	-4.37
nubbe744uff	-4.37
fqnp135	-4.37

zinc000000114127	-4.37
zinc000001530736	-4.37
zinc000008101127	-4.37
zinc000012503156	-4.37
zinc000019632718	-4.37
zinc06185615	-4.37
zinc91914945	-4.37
nubbe192	-4.36
nubbe2221	-4.36
perunpdb54	-4.36
zinc000000000469	-4.36
zinc000011615928	-4.36
zinc000017285872	-4.36
zinc03786134	-4.36
zinc96327090	-4.36
nubbe1104	-4.35
nubbe1383	-4.35
fqnp439	-4.35
fqnp467	-4.35
zinc000008015016	-4.35
zinc34588843	-4.35
nubbe106	-4.34
nubbe670uff	-4.34
perunpdb168	-4.34
perunpdb75	-4.34
perunpdb8	-4.34
zinc000100004345	-4.34
zinc03874426	-4.34
zinc05133855	-4.34
zinc42305220	-4.34
zinc76598761	-4.34
zinc96331712	-4.34
nubbe123uff	-4.33
zinc15768134	-4.33
nubbe1364	-4.32
nubbe1479	-4.32
nubbe1667	-4.32
nubbe1688	-4.32
nubbe1736	-4.32
nubbe183uff	-4.32
nubbe214	-4.32
nubbe2461	-4.32
nubbe321uff	-4.32
nubbe404uff	-4.32

perunpdb49	-4.32
zinc000000001706	-4.32
zinc57219206	-4.32
zinc76632232	-4.32
nubbe979	-4.31
fqnp364	-4.31
zinc000011615927	-4.31
zinc13516646	-4.31
zinc82846295	-4.31
nubbe103uff	-4.3
nubbe1510	-4.3
nubbe2135	-4.3
nubbe2176	-4.3
nubbe222uff	-4.3
nubbe2459	-4.3
nubbe370uff	-4.3
nubbe8	-4.3
zinc000000537957	-4.3
zinc000001530567	-4.3
zinc000001546066	-4.3
zinc000005133378	-4.3
zinc22061979	-4.3
zinc42227693	-4.3
zinc76598758	-4.3
nubbe11	-4.29
nubbe2226	-4.29
nubbe2477	-4.29
nubbe61	-4.29
nubbe626uff	-4.29
zinc000003806262	-4.29
zinc000018099446	-4.29
zinc000029416466	-4.29
zinc000038212689	-4.29
zinc000100052688	-4.29
zinc01688588	-4.29
zinc12153281	-4.29
zinc37688189	-4.29
zinc82399752	-4.29
nubbe1198	-4.28
nubbe127uff	-4.28
nubbe171	-4.28
nubbe2114	-4.28
nubbe226	-4.28
nubbe242uff	-4.28

nubbe29	-4.28
nubbe44uff	-4.28
perunpdb272	-4.28
zinc000000018087	-4.28
zinc000003871978	-4.28
zinc000035024346	-4.28
zinc03795332	-4.28
zinc03875041	-4.28
zinc71244991	-4.28
zinc76598759	-4.28
nubbe1156	-4.27
nubbe321	-4.27
nubbe637buff	-4.27
zinc05840346	-4.27
nubbe1111	-4.26
nubbe629uff	-4.26
zinc05375997	-4.26
zinc39116165	-4.26
zinc82405552	-4.26
nubbe1975	-4.25
nubbe2107	-4.25
perunpdb10	-4.25
perunpdb41	-4.25
zinc000004216238	-4.25
zinc34332711	-4.25
zinc38950861	-4.25
nubbe184	-4.24
nubbe2121	-4.24
nubbe359uff	-4.24
nubbe394uff	-4.24
nubbe447	-4.24
nubbe652uff	-4.24
zinc000003918138	-4.24
zinc000012466082	-4.24
zinc34452792	-4.24
zinc38867481	-4.24
zinc39103504	-4.24
zinc95078832	-4.24
nubbe1375	-4.23
nubbe1384	-4.23
nubbe212uff	-4.23
nubbe227uff	-4.23
perunpdb29	-4.23

perunpdb96	-4.23
fqnp33	-4.23
zinc000000000711	-4.23
zinc000000014864	-4.23
zinc000029319828	-4.23
zinc06562434	-4.23
nubbe1243	-4.22
nubbe1951	-4.22
nubbe423	-4.22
nubbe442uff	-4.22
zinc000000000122	-4.22
zinc000004658290	-4.22
zinc61992342	-4.22
zinc82399748	-4.22
nubbe374uff	-4.21
nubbe51	-4.21
zinc000003831040	-4.21
zinc000022016976	-4.21
zinc13483728	-4.21
nubbe2181	-4.2
nubbe2457	-4.2
nubbe311	-4.2
fqnp5	-4.2
zinc000000000882	-4.2
zinc000001530759	-4.2
zinc000013973998	-4.2
zinc35151522	-4.2
nubbe1179	-4.19
nubbe515	-4.19
nubbe7uff	-4.19
perunpdb77	-4.19
fqnp139	-4.19
fqnp311	-4.19
zinc000001531036	-4.19
zinc000049783788	-4.19
zinc38522789	-4.19
zinc57219209	-4.19
nubbe1604	-4.18
nubbe1650	-4.18
nubbe1682	-4.18
zinc000003798064	-4.18
zinc000003809490	-4.18
zinc000003830947	-4.18

zinc000003831165	-4.18
zinc000013587680	-4.18
zinc38950571	-4.18
zinc40733757	-4.18
nubbe1178	-4.17
nubbe1958	-4.17
nubbe369uff	-4.17
nubbe419	-4.17
nubbe423uff	-4.17
nubbe57uff	-4.17
fqnp443	-4.17
zinc000000002041	-4.17
zinc000000518554	-4.17
zinc000001530659	-4.17
zinc000008101126	-4.17
zinc000011680943	-4.17
zinc02528515	-4.17
zinc49934103	-4.17
zinc65454681	-4.17
nubbe308	-4.16
nubbe343	-4.16
nubbe632uff	-4.16
perunpdb40	-4.16
fqnp4	-4.16
zinc000000003911	-4.16
zinc000001530706	-4.16
zinc000004654889	-4.16
zinc000009574770	-4.16
zinc21798209	-4.16
zinc35567010	-4.16
zinc82399746	-4.16
nubbe1378	-4.15
nubbe1681	-4.15
nubbe2142	-4.15
nubbe230uff	-4.15
nubbe319uff	-4.15
nubbe335uff	-4.15
nubbe46	-4.15
zinc000003978029	-4.15
zinc000014210876	-4.15
zinc34753985	-4.15
zinc37688190	-4.15
nubbe1702	-4.14
nubbe2123	-4.14

nubbe2329	-4.14
nubbe374	-4.14
zinc000000155531	-4.14
zinc000001530939	-4.14
zinc000001550499	-4.14
zinc000003875259	-4.14
zinc000003920355	-4.14
zinc76592636	-4.14
zinc76593293	-4.14
nubbe283uff	-4.13
perunpdb56	-4.13
zinc000004097310	-4.13
zinc38522792	-4.13
zinc39059944	-4.13
zinc39098130	-4.13
zinc76593305	-4.13
nubbe1164	-4.12
perunpdb66	-4.12
zinc000000004009	-4.12
zinc000000007601	-4.12
zinc000001530811	-4.12
zinc000003795098	-4.12
zinc42207553	-4.12
zinc76632279	-4.12
zinc95078834	-4.12
nubbe1219	-4.11
nubbe1676	-4.11
nubbe47	-4.11
nubbe52	-4.11
nubbe675	-4.11
zinc000003813078	-4.11
zinc05572699	-4.11
nubbe284	-4.1
nubbe418uff	-4.1
nubbe60	-4.1
nubbe930	-4.1
fqnp323	-4.1
zinc000001529323	-4.1
zinc000003831551	-4.1
zinc57219211	-4.1
nubbe1201	-4.09
nubbe1883	-4.09
nubbe50uff	-4.09
nubbe767uff	-4.09

perunpdb278	-4.09
perunpdb71	-4.09
zinc000000001115	-4.09
zinc000000014007	-4.09
zinc000000266964	-4.09
zinc000000601281	-4.09
zinc000001530427	-4.09
zinc000004097225	-4.09
zinc000004658560	-4.09
zinc37490673	-4.09
zinc39097912	-4.09
nubbe108	-4.08
nubbe1809	-4.08
nubbe222	-4.08
nubbe370	-4.08
nubbe418	-4.08
zinc000003806413	-4.08
zinc000003843378	-4.08
zinc04915559	-4.08
zinc19089931	-4.08
zinc38951335	-4.08
nubbe1799	-4.07
nubbe454uff	-4.07
zinc42469138	-4.07
fqnp464	-4.06
zinc000000968255	-4.06
zinc000003920719	-4.06
zinc51887249	-4.06
nubbe1249	-4.05
nubbe1775	-4.05
nubbe2219	-4.05
nubbe239	-4.05
perunpdb82	-4.05
zinc000000896543	-4.05
zinc000001530713	-4.05
zinc000001532517	-4.05
zinc000036520252	-4.05
zinc000095619100	-4.05
zinc51887248	-4.05
zinc96327103	-4.05
nubbe137uff	-4.04
nubbe1774	-4.04
nubbe208uff	-4.04

nubbe37	-4.04
nubbe47uff	-4.04
perunpdb199	-4.04
perunpdb247	-4.04
zinc000257553302	-4.04
zinc14680517	-4.04
zinc35567006	-4.04
zinc82393088	-4.04
nubbe1640	-4.03
nubbe186	-4.03
nubbe2109	-4.03
zinc000001530568	-4.03
zinc000003812974	-4.03
zinc000003871703	-4.03
zinc62454728	-4.03
nubbe1644	-4.02
nubbe1691	-4.02
nubbe351	-4.02
nubbe927	-4.02
fqnp193	-4.02
zinc000000049153	-4.02
zinc000003831475	-4.02
zinc000100017404	-4.02
zinc03957104	-4.02
zinc35298061	-4.02
zinc35298586	-4.02
nubbe1705	-4.01
fqnp466	-4.01
zinc000000599985	-4.01
zinc000001530572	-4.01
zinc000003784120	-4.01
nubbe1804	-4
nubbe1993	-4
nubbe214uff	-4
nubbe398	-4
fqnp158	-4
zinc000000005152	-4
zinc000003781943	-4
zinc000004097283	-4
zinc38810082	-4
zinc38810084	-4
zinc39060307	-4
zinc57219208	-4

nubbe2151	-3.99
nubbe2211	-3.99
nubbe2464	-3.99
nubbe768uff	-3.99
fqnp148	-3.99
zinc000000057278	-3.99
zinc000100016058	-3.99
zinc96029763	-3.99
nubbe1870	-3.98
zinc000000001590	-3.98
zinc000000901736	-3.98
zinc000003798750	-3.98
zinc000003995811	-3.98
zinc000004655029	-3.98
zinc000085537026	-3.98
zinc03874427	-3.98
zinc05829940	-3.98
zinc35298063	-3.98
nubbe1253	-3.97
nubbe184uff	-3.97
nubbe362	-3.97
fqnp138	-3.97
zinc39097840	-3.97
zinc61992341	-3.97
nubbe1260	-3.96
nubbe1379	-3.96
fqnp199	-3.96
zinc000000033882	-3.96
zinc000001530283	-3.96
zinc000001530636	-3.96
zinc000003807172	-3.96
zinc000004217732	-3.96
zinc000009212428	-3.96
zinc000019166991	-3.96
nubbe1778	-3.95
nubbe424	-3.95
nubbe578	-3.95
zinc000000155694	-3.95
zinc000000389149	-3.95
zinc000001530579	-3.95
zinc000003798247	-3.95
zinc000019364242	-3.95
zinc61992333	-3.95
nubbe2446	-3.94

nubbe351uff	-3.94
perunpdb279	-3.94
fqnp319	-3.94
zinc000000897256	-3.94
zinc95078833	-3.94
nubbe1833	-3.93
nubbe1918	-3.93
zinc000001187543	-3.93
zinc05131867	-3.93
zinc42375787	-3.93
zinc75421741	-3.93
zinc91914946	-3.93
nubbe2106	-3.92
nubbe545	-3.92
zinc000043202140	-3.92
zinc01081507	-3.92
zinc76591237	-3.92
zinc82399753	-3.92
nubbe1166	-3.91
nubbe119uff	-3.91
nubbe1245	-3.91
nubbe1608	-3.91
nubbe2125	-3.91
nubbe974	-3.91
perunpdb201	-3.91
zinc000000001673	-3.91
zinc000002015035	-3.91
zinc000004097427	-3.91
zinc000006827693	-3.91
zinc000008214573	-3.91
zinc000011677857	-3.91
zinc38996892	-3.91
perunpdb170	-3.9
zinc000001489478	-3.9
zinc000164760756	-3.9
nubbe1184	-3.89
nubbe639	-3.89
perunpdb169	-3.89
perunpdb246	-3.89
zinc000001530803	-3.89
zinc35151520	-3.89
nubbe1642	-3.88
nubbe307	-3.88
zinc000001482113	-3.88

zinc000002019953	-3.88
zinc38522791	-3.88
zinc39088370	-3.88
nubbe102uff	-3.87
nubbe1399	-3.87
nubbe1791	-3.87
nubbe1974	-3.87
nubbe205uff	-3.87
nubbe369	-3.87
nubbe60uff	-3.87
nubbe670	-3.87
zinc000001530820	-3.87
zinc000002539702	-3.87
zinc39116171	-3.87
nubbe232	-3.86
perunpdb46	-3.86
zinc000000403618	-3.86
zinc000003830842	-3.86
zinc000004095696	-3.86
zinc000004658553	-3.86
zinc000019168887	-3.86
zinc22061976	-3.86
zinc38522790	-3.86
nubbe2460	-3.85
nubbe281	-3.85
perunpdb192	-3.85
zinc65743303	-3.85
nubbe213uff	-3.84
nubbe2149	-3.84
nubbe225	-3.84
nubbe425uff	-3.84
nubbe758uff	-3.84
zinc000001554010	-3.84
zinc000003938482	-3.84
zinc000003982483	-3.84
zinc000022016981	-3.84
zinc65739542	-3.84
nubbe1566	-3.83
nubbe2144	-3.83
zinc000003842753	-3.83
zinc000004095858	-3.83
zinc000086040406	-3.83
zinc38433750	-3.83

nubbe1395	-3.82
nubbe248uff	-3.82
nubbe2uff	-3.82
perunpdb210	-3.82
zinc000001530938	-3.82
zinc000001633887	-3.82
zinc000002519740	-3.82
nubbe1628	-3.81
nubbe1878	-3.81
nubbe1905	-3.81
nubbe535uff	-3.81
perunpdb167	-3.81
zinc000000014037	-3.81
zinc000000388081	-3.81
zinc000011616925	-3.81
zinc77292286	-3.81
zinc95078831	-3.81
zinc96327117	-3.81
nubbe211	-3.8
nubbe2455	-3.8
nubbe419uff	-3.8
nubbe96uff	-3.8
nubbe980	-3.8
zinc000000403011	-3.8
zinc000001530303	-3.8
zinc000003782550	-3.8
zinc000003995809	-3.8
nubbe2116	-3.79
nubbe417	-3.79
nubbe641uff	-3.79
perunpdb19	-3.79
zinc22055286	-3.79
nubbe1866	-3.78
nubbe456uff	-3.78
nubbe637uff	-3.78
nubbe756uff	-3.78
zinc000000000083	-3.78
zinc000003929508	-3.78
zinc000008214418	-3.78
zinc000072267023	-3.78
nubbe1805	-3.77
nubbe48uff	-3.77
perunpdb13	-3.77

zinc000049933061	-3.77
zinc000100074252	-3.77
zinc03775177	-3.77
zinc22055292	-3.77
zinc39060246	-3.77
nubbe129	-3.76
nubbe1525	-3.76
zinc000003079336	-3.76
nubbe117uff	-3.75
nubbe1497	-3.75
nubbe1808	-3.75
zinc000001538857	-3.75
zinc000003830314	-3.75
zinc000019419017	-3.75
nubbe1498	-3.74
nubbe273uff	-3.74
zinc000003805768	-3.74
zinc000085432544	-3.74
zinc13719785	-3.74
zinc71789999	-3.74
nubbe1376	-3.73
nubbe1794	-3.73
nubbe641	-3.73
zinc000001542002	-3.73
zinc000001698306	-3.73
zinc000002599970	-3.73
zinc000011616852	-3.73
zinc38027459	-3.73
perunpdb211	-3.72
perunpdb70	-3.72
zinc000003794711	-3.72
zinc000003812863	-3.72
zinc01631208	-3.72
zinc06562433	-3.72
zinc37490675	-3.72
zinc39088368	-3.72
nubbe1296	-3.71
nubbe16uff	-3.71
nubbe273	-3.71
nubbe510	-3.71
perunpdb200	-3.71
zinc000000049154	-3.71
zinc000000897288	-3.71
zinc000003952881	-3.71

zinc000034676245	-3.71
zinc000100061056	-3.71
nubbe1677	-3.7
nubbe1686	-3.7
nubbe372uff	-3.7
nubbe535	-3.7
zinc000003581355	-3.7
zinc000003830387	-3.7
zinc38363215	-3.7
zinc96327085	-3.7
nubbe131	-3.69
nubbe1973	-3.69
nubbe2108	-3.69
nubbe56uff	-3.69
perunpdb47	-3.69
perunpdb81	-3.69
zinc000000000856	-3.69
zinc000000002005	-3.69
zinc000100022637	-3.69
zinc15442204	-3.69
zinc39097858	-3.69
nubbe167	-3.68
nubbe322	-3.68
fqnp123	-3.68
zinc000000057146	-3.68
zinc000000155905	-3.68
zinc000003791297	-3.68
zinc06385059	-3.68
zinc39060242	-3.68
nubbe1704	-3.67
nubbe2143	-3.67
nubbe56	-3.67
perunpdb173	-3.67
zinc000000403609	-3.67
zinc000001530652	-3.67
zinc000003830959	-3.67
zinc000004658562	-3.67
zinc000085534336	-3.67
zinc15442203	-3.67
zinc40186615	-3.67
zinc76599673	-3.67
nubbe1565	-3.66
nubbe2146	-3.66
nubbe270	-3.66

perunpdb172	-3.66
fqnp429	-3.66
zinc000000000494	-3.66
zinc000002539484	-3.66
nubbe1386	-3.65
nubbe378uff	-3.65
zinc000000057147	-3.65
zinc000001543916	-3.65
zinc000003981610	-3.65
zinc000013818943	-3.65
zinc000100006770	-3.65
nubbe46uff	-3.64
nubbe6uff	-3.64
fqnp273	-3.64
zinc000000896546	-3.64
zinc000001530762	-3.64
zinc000003830453	-3.64
zinc000004474405	-3.64
zinc000252286876	-3.64
nubbe117	-3.63
nubbe39	-3.63
nubbe635	-3.63
zinc000001843099	-3.63
zinc000003831050	-3.63
nubbe1987	-3.62
fqnp390	-3.62
zinc000000895099	-3.62
zinc000253529561	-3.62
zinc03794711	-3.62
nubbe975	-3.61
perunpdb194	-3.61
perunpdb197	-3.61
zinc000002169830	-3.61
zinc000100052681	-3.61
nubbe1295	-3.6
nubbe748uff	-3.6
perunpdb171	-3.6
fqnp53	-3.6
zinc000001530718	-3.6
zinc000003830713	-3.6
zinc000004658603	-3.6
zinc000100070233	-3.6
zinc02077807	-3.6

zinc48857970	-3.6
zinc77292284	-3.6
nubbe2120	-3.59
nubbe457uff	-3.59
zinc21798212	-3.59
zinc76599400	-3.59
nubbe2124	-3.58
zinc000001530639	-3.58
zinc000008034120	-3.58
zinc35649798	-3.58
nubbe452	-3.57
fqnp470	-3.57
zinc000001530654	-3.57
zinc22054075	-3.57
zinc36533584	-3.57
zinc000001529266	-3.56
zinc38864631	-3.56
nubbe1185	-3.55
nubbe1972	-3.55
nubbe443uff	-3.55
perunpdb59	-3.55
zinc000000394284	-3.55
zinc39088366	-3.55
nubbe1084	-3.54
nubbe176uff	-3.54
zinc000000114124	-3.54
zinc000006661227	-3.54
zinc38433749	-3.54
zinc39089965	-3.54
nubbe107	-3.53
nubbe185	-3.53
fqnp120	-3.53
zinc000028467879	-3.53
nubbe233	-3.52
perunpdb50	-3.52
fqnp458	-3.52
fqnp460	-3.52
zinc000001482049	-3.52
zinc000001540998	-3.52
zinc000001995484	-3.52
zinc000003812867	-3.52
nubbe213	-3.51
perunpdb80	-3.51

zinc000001530660	-3.51
zinc000072320327	-3.51
zinc000100019007	-3.51
nubbe116	-3.5
nubbe1884	-3.5
nubbe274uff	-3.5
nubbe304uff	-3.5
nubbe743uff	-3.5
zinc04098894	-3.5
zinc11681339	-3.5
nubbe1643	-3.49
nubbe913	-3.49
zinc000085432549	-3.49
zinc000085540223	-3.49
nubbe1190	-3.48
nubbe173	-3.48
nubbe173uff	-3.48
nubbe2110	-3.48
zinc000145401681	-3.48
nubbe1690	-3.47
nubbe339	-3.47
zinc000043194409	-3.47
zinc13545227	-3.47
nubbe104uff	-3.46
nubbe413	-3.46
perunpdb202	-3.46
zinc000001530575	-3.46
zinc000003978028	-3.46
zinc000004214955	-3.46
zinc000005224188	-3.46
zinc38234157	-3.46
zinc76599396	-3.46
nubbe107uff	-3.45
nubbe1645	-3.45
nubbe1684	-3.45
nubbe307uff	-3.45
perunpdb248	-3.45
zinc000001530717	-3.44
zinc000253529562	-3.44
nubbe303uff	-3.43
perunpdb12	-3.43
zinc000003995807	-3.43
zinc43419392	-3.43
zinc000000901552	-3.42

zinc000003803652	-3.42
zinc000012468792	-3.42
nubbe1503	-3.41
nubbe1683	-3.41
nubbe1802	-3.41
nubbe1998	-3.41
zinc000001530728	-3.41
zinc000003831051	-3.41
nubbe288uff	-3.4
nubbe875	-3.4
zinc000001530775	-3.4
zinc38433752	-3.4
nubbe389	-3.39
nubbe632	-3.39
fqnp200	-3.39
zinc000000895040	-3.39
nubbe114uff	-3.38
nubbe1703	-3.38
nubbe1876	-3.38
fqnp39	-3.38
zinc39088369	-3.38
nubbe123	-3.37
nubbe166uff	-3.37
nubbe1971	-3.37
zinc000008214402	-3.37
zinc11681341	-3.37
nubbe280uff	-3.36
nubbe282	-3.36
perunpdb275	-3.36
zinc000001532529	-3.36
zinc000008551180	-3.36
nubbe285	-3.35
nubbe311uff	-3.35
nubbe453	-3.35
perunpdb69	-3.35
perunpdb74	-3.35
fqnp137	-3.35
zinc000001532805	-3.35
nubbe1792	-3.34
nubbe2145	-3.34
zinc000000000353	-3.34
zinc000000538509	-3.34
zinc000003806063	-3.34
nubbe978	-3.33

perunpdb190	-3.33
zinc000001529425	-3.33
zinc000004658552	-3.33
zinc000008437287	-3.33
zinc000028957444	-3.33
zinc000253529560	-3.33
zinc38864628	-3.33
nubbe270uff	-3.32
nubbe560	-3.32
zinc000000901061	-3.32
zinc000001530569	-3.32
zinc000002016052	-3.32
zinc000005819214	-3.32
zinc000053022902	-3.32
nubbe1880	-3.31
nubbe303	-3.31
nubbe341uff	-3.31
zinc000000000257	-3.31
zinc000001487602	-3.31
zinc000001530635	-3.31
zinc000001530737	-3.31
zinc000001532728	-3.31
zinc000003830957	-3.31
zinc000029571072	-3.31
zinc39937087	-3.31
zinc62454731	-3.31
nubbe205	-3.29
zinc000000001505	-3.29
zinc000000002176	-3.29
zinc000100052691	-3.29
nubbe167uff	-3.28
perunpdb15	-3.28
zinc000001530580	-3.28
zinc000003785276	-3.28
zinc000252678020	-3.28
nubbe1505	-3.27
nubbe230	-3.27
zinc39088367	-3.27
nubbe1793	-3.26
zinc000004658557	-3.26
zinc38864637	-3.26
nubbe124	-3.25
zinc000000895103	-3.25

zinc000003800475	-3.25
zinc39088365	-3.25
nubbe51uff	-3.24
nubbe226uff	-3.23
zinc000003079342	-3.23
zinc000004474564	-3.23
zinc39088364	-3.23
nubbe194uff	-3.22
nubbe312uff	-3.22
perunpdb191	-3.22
zinc000008214514	-3.22
zinc38433751	-3.22
nubbe456	-3.21
perunpdb94	-3.21
perunpdb212	-3.2
perunpdb9	-3.2
zinc000035454991	-3.2
nubbe324uff	-3.19
nubbe636	-3.19
nubbe759uff	-3.19
zinc42375786	-3.19
nubbe1377	-3.18
nubbe38	-3.18
zinc000000895042	-3.18
zinc000001542392	-3.18
zinc76599670	-3.18
nubbe1397	-3.17
nubbe62uff	-3.17
zinc000003079340	-3.17
zinc000003977737	-3.17
zinc05688736	-3.17
nubbe324	-3.16
perunpdb14	-3.16
zinc13511397	-3.16
zinc38864633	-3.16
nubbe106uff	-3.15
zinc000003941496	-3.15
zinc02508225	-3.15
nubbe603	-3.14
zinc000000113415	-3.13
zinc000003812913	-3.13
zinc000006845860	-3.13
zinc000008143864	-3.13

zinc000035454995	-3.13
nubbe302uff	-3.12
nubbe312	-3.12
nubbe1811	-3.11
nubbe981	-3.11
zinc000001532526	-3.11
nubbe2167	-3.1
zinc000000895316	-3.1
zinc000003813088	-3.1
zinc000008220878	-3.1
nubbe318uff	-3.09
zinc000003939013	-3.09
nubbe1820	-3.08
nubbe211uff	-3.08
zinc000001532522	-3.08
zinc39937088	-3.08
nubbe17	-3.07
nubbe37uff	-3.07
zinc000000000507	-3.07
zinc08627075	-3.07
zinc36378001	-3.07
nubbe1821	-3.06
nubbe334	-3.06
zinc000001530600	-3.06
zinc000003830958	-3.06
zinc22054059	-3.06
nubbe1803	-3.05
nubbe233uff	-3.05
nubbe310uff	-3.05
fqnp428	-3.05
zinc000004474603	-3.05
nubbe111uff	-3.04
zinc000003831138	-3.04
zinc000100001964	-3.04
nubbe642uff	-3.03
zinc000095617679	-3.03
nubbe172uff	-3.02
nubbe448uff	-3.02
zinc000003872952	-3.02
nubbe231	-3.01
nubbe50	-3.01
zinc22053713	-3.01
nubbe1795	-3
perunpdb73	-3

nubbe2113	-2.99
zinc000000901159	-2.99
nubbe2471	-2.98
zinc000001535101	-2.98
zinc000003801919	-2.98
zinc01593341	-2.98
nubbe17uff	-2.97
fqnp405	-2.97
fqnp94	-2.97
zinc000000901555	-2.97
zinc000000403079	-2.96
zinc000003079337	-2.96
zinc000008551178	-2.96
zinc000012503177	-2.96
zinc000253529563	-2.96
zinc000000895034	-2.95
zinc000004097426	-2.95
zinc000008034121	-2.95
nubbe1186	-2.94
nubbe1689	-2.94
nubbe660uff	-2.94
zinc000001532525	-2.94
zinc000003794794	-2.94
zinc000100001965	-2.94
nubbe65uff	-2.93
perunpdb195	-2.93
nubbe1567	-2.92
nubbe1679	-2.92
fqnp224	-2.92
zinc04523240	-2.92
nubbe636uff	-2.91
nubbe766uff	-2.91
nubbe309uff	-2.9
zinc000242437512	-2.9
nubbe231uff	-2.89
nubbe299	-2.89
fqnp226	-2.89
zinc39097926	-2.89
nubbe283	-2.88
nubbe339uff	-2.88
zinc05157115	-2.88
zinc22054067	-2.88
nubbe1032	-2.87
zinc000003872605	-2.87

nubbe116uff	-2.86
nubbe206uff	-2.86
nubbe560uff	-2.86
zinc000003830813	-2.86
zinc04523242	-2.86
perunpdb72	-2.85
zinc000011615926	-2.84
zinc000049637509	-2.84
zinc04533798	-2.84
zinc06562444	-2.84
zinc22054079	-2.84
nubbe1699	-2.83
nubbe1810	-2.83
nubbe186uff	-2.83
nubbe1796	-2.82
perunpdb18	-2.82
zinc000000896695	-2.82
zinc02562349	-2.82
nubbe212	-2.81
zinc000003875342	-2.81
zinc000003830945	-2.8
zinc000003871832	-2.8
nubbe206	-2.79
zinc000003812306	-2.78
zinc000003944422	-2.78
zinc04566616	-2.78
nubbe104	-2.77
nubbe1641	-2.77
perunpdb17	-2.77
zinc000001532179	-2.77
zinc000242437511	-2.77
nubbe282uff	-2.76
zinc000003785268	-2.76
zinc05063416	-2.76
nubbe169	-2.75
zinc000003830943	-2.75
zinc000085537053	-2.75
zinc36533537	-2.74
nubbe168uff	-2.73
nubbe301	-2.72
zinc000001554392	-2.71
zinc000008035377	-2.71
nubbe2472	-2.7

zinc98089939	-2.7
zinc000052971887	-2.69
zinc36387027	-2.69
perunpdb203	-2.68
zinc000003812960	-2.68
zinc000001530760	-2.66
zinc000514288136	-2.66
zinc04404234	-2.66
nubbe223uff	-2.65
zinc000000156792	-2.65
zinc000001846431	-2.65
nubbe193uff	-2.64
nubbe420	-2.63
zinc000000895318	-2.63
zinc000245224194	-2.63
nubbe172	-2.62
nubbe318	-2.62
fqnp149	-2.62
zinc22054071	-2.62
zinc000100052685	-2.61
zinc000150338698	-2.61
nubbe2332	-2.6
nubbe760uff	-2.6
nubbe963	-2.6
zinc000008214703	-2.59
nubbe330uff	-2.58
zinc000085540219	-2.58
nubbe451uff	-2.57
zinc000001531008	-2.57
nubbe193	-2.56
nubbe176	-2.55
nubbe2423	-2.55
nubbe48	-2.55
zinc000008214681	-2.55
nubbe236	-2.54
zinc000003812888	-2.54
zinc24817559	-2.54
nubbe281uff	-2.53
nubbe452uff	-2.53
nubbe769uff	-2.53
nubbe1031	-2.52
nubbe187uff	-2.52
nubbe302	-2.51

nubbe448	-2.51
nubbe236uff	-2.5
zinc000028232746	-2.5
perunpdb93	-2.49
nubbe114	-2.48
nubbe1162	-2.48
nubbe332uff	-2.48
zinc000000895048	-2.48
zinc05167490	-2.46
nubbe1157	-2.44
nubbe210	-2.43
nubbe272uff	-2.43
nubbe301uff	-2.43
zinc000100009383	-2.43
nubbe313uff	-2.42
nubbe340uff	-2.42
zinc000514288134	-2.42
fqnp121	-2.41
zinc000003830635	-2.41
zinc04566615	-2.41
zinc000085540215	-2.4
zinc13532457	-2.4
nubbe208	-2.38
nubbe389uff	-2.38
nubbe636buff	-2.38
nubbe642	-2.36
fqnp116	-2.36
zinc000003951740	-2.36
zinc000004097392	-2.36
nubbe1187	-2.35
nubbe2454	-2.35
zinc000253500687	-2.34
zinc000019364219	-2.32
nubbe454	-2.31
nubbe142	-2.3
nubbe169uff	-2.3
nubbe337	-2.3
nubbe443	-2.3
nubbe445	-2.3
zinc000027641461	-2.3
nubbe603uff	-2.29
nubbe63	-2.29
zinc000035454987	-2.29
zinc000049841054	-2.29
nubbe204uff	-2.28
fqnp430	-2.28
zinc000003776875	-2.28
nubbe987	-2.27
zinc000242437513	-2.27
fqnp431	-2.26
zinc000003830891	-2.26
zinc000008035395	-2.26
zinc000216450933	-2.25
nubbe192uff	-2.24
fqnp151	-2.24
zinc000000643046	-2.24
nubbe110uff	-2.23
nubbe207uff	-2.23
nubbe337uff	-2.23
nubbe204	-2.22
fqnp463	-2.22
nubbe1088	-2.21
nubbe1801	-2.21
nubbe453uff	-2.2
zinc000008577218	-2.2
nubbe174uff	-2.19
nubbe194	-2.19
nubbe1800	-2.18
zinc000257355149	-2.18
nubbe223	-2.17
nubbe335	-2.16
nubbe185uff	-2.15
zinc000003955219	-2.15
nubbe110	-2.14
nubbe313	-2.14
zinc000003830946	-2.14
zinc02020190	-2.14
zinc000004102194	-2.13
zinc38644575	-2.13
zinc000008551179	-2.12
nubbe635uff	-2.1
zinc000038238855	-2.1
nubbe272	-2.07
zinc13532465	-2.07
nubbe103	-2.06
perunpdb89	-2.06
zinc000003941829	-2.06
zinc000008035268	-2.06

zinc98089938	-2.05
fqnp122	-2.03
zinc000003991624	-2.03
nubbe1167	-2.02
nubbe1985	-2.02
zinc000019364225	-2.02
nubbe111	-2.01
fqnp432	-2.01
zinc000003830960	-2.01
zinc000100055899	-2.01
zinc000514288311	-2.01
fqnp291	-2
zinc000008214635	-2
zinc000087496092	-2
nubbe109	-1.99
nubbe124uff	-1.99
zinc000103650667	-1.99
nubbe331	-1.97
zinc000008214651	-1.96
zinc000001530810	-1.9
zinc000004474682	-1.9
zinc000257355148	-1.89
nubbe109uff	-1.88
zinc000006920384	-1.88
nubbe125uff	-1.86
nubbe330	-1.86
zinc000035454983	-1.86
zinc000257355145	-1.83
zinc000150339323	-1.82
nubbe112uff	-1.81
zinc04566614	-1.8
fqnp67	-1.79
zinc000003799072	-1.78
zinc98089940	-1.78
nubbe210uff	-1.77
zinc000018279893	-1.77
nubbe118	-1.76
nubbe174	-1.73
nubbe332	-1.73
zinc000004217475	-1.73
zinc98089944	-1.73
nubbe1649	-1.72
zinc000003977952	-1.72

zinc000085536932	-1.72
zinc000009164421	-1.7
nubbe604	-1.68
zinc000004393164	-1.67
nubbe112	-1.66
zinc000001533877	-1.62
zinc000008551177	-1.62
zinc98089943	-1.61
zinc02556386	-1.58
nubbe118uff	-1.56
zinc000002041302	-1.56
nubbe420uff	-1.55
nubbe635buff	-1.55
zinc000514288135	-1.55
zinc000001543475	-1.54
zinc000003943279	-1.54
zinc000019363537	-1.51
zinc000085537014	-1.47
perunpdb90	-1.42
zinc000038945666	-1.38
zinc000257355146	-1.38
nubbe187	-1.35
nubbe207	-1.35
nubbe444	-1.33
zinc000242437514	-1.33
zinc000008214614	-1.31
zinc000003830944	-1.29
zinc000238809664	-1.29
zinc000003930376	-1.26
perunpdb86	-1.22
nubbe602	-1.2
nubbe546	-1.19
perunpdb85	-1.18
zinc04566617	-1.18
nubbe331uff	-1.17
nubbe1806	-1.16
perunpdb88	-1.14
zinc000014881137	-1.09
zinc000238809662	-1.06
zinc000000896409	-1.05
perunpdb87	-1.02
fqnp434	-1.01
zinc000008214692	-1.01

nubbe338	-0.94		zinc000095564694	0.21
zinc000238809665	-0.93		zinc000060392785	0.26
nubbe2274	-0.83		perunpdb83	0.3
nubbe1807	-0.82		zinc000060183170	0.33
nubbe280	-0.8		zinc000022448097	0.35
zinc000028108825	-0.78		zinc000085537017	0.46
zinc000003929022	-0.71		perunpdb84	0.48
zinc000008214483	-0.7		fqnp74	1.03
zinc000257355147	-0.62		zinc000033965961	1.25
nubbe390	-0.55		zinc000022851765	1.63
nubbe286	-0.54		zinc000003830276	1.72
zinc000257355150	-0.46		zinc000021982937	1.89
zinc000096006013	-0.35		nubbe361	2.21
perunpdb92	-0.3		zinc000019855121	2.32
perunpdb91	-0.11		fqnp73	2.36
zinc000238809663	-0.06		zinc000022447798	2.52
nubbe360	-0.03		zinc000096309558	2.89
nubbe271	0.1			

Table S2. PASSer predictions for allosteric site probabilities in MOGS protein pockets.

Pocket Number	Parameter	Amount
Pocket 1		
	Score	0.118
	Druggability Score	0.006
	Number of Alpha Spheres	50.000
	Total SASA	130.671
	Polar SASA	69.082
	Apolar SASA	61.589
	Volume	488.359
	Mean local hydrophobic density	5.250
	Mean alpha sphere radius	3.932
	Mean alp. sph. solvent access	0.707
	Apolar alpha sphere proportion	0.160
	Hydrophobicity score	-3.000
	Volume score	3.308
	Polarity score	10.000
	Charge score	-2.000
	Proportion of polar atoms	51.351
	Alpha sphere density	6.177
	Cent. of mass - Alpha Sphere max dist	14.690
	Flexibility	0.922

Pocket 2

Score	0.084
Druggability Score	0.002
Number of Alpha Spheres	20.000
Total SASA	18.505
Polar SASA	6.429
Apolar SASA	12.076
Volume	96.898
Mean local hydrophobic density	7.000
Mean alpha sphere radius	3.624
Mean alp. sph. solvent access	0.414
Apolar alpha sphere proportion	0.400
Hydrophobicity score	9.889
Volume score	4.556
Polarity score	5.000
Charge score	2.000
Proportion of polar atoms	40.000
Alpha sphere density	1.723
Cent. of mass - Alpha Sphere max dist	3.745
Flexibility	0.897

Pocket 3

Score	0.074
Druggability Score	0.001
Number of Alpha Spheres	19.000
Total SASA	11.554
Polar SASA	4.308
Apolar SASA	7.246
Volume	83.399
Mean local hydrophobic density	8.000
Mean alpha sphere radius	3.606
Mean alp. sph. solvent access	0.454
Apolar alpha sphere proportion	0.474
Hydrophobicity score	-5.000
Volume score	4.500
Polarity score	6.000
Charge score	1.000
Proportion of polar atoms	33.333
Alpha sphere density	1.325
Cent. of mass - Alpha Sphere max dist	2.830
Flexibility	0.941

Pocket 4

Score	0.066

Druggability Score	0.541
Number of Alpha Spheres	53.000
Total SASA	151.351
Polar SASA	48.703
Apolar SASA	102.648
Volume	648.796
Mean local hydrophobic density	20.857
Mean alpha sphere radius	3.872
Mean alp. sph. solvent access	0.530
Apolar alpha sphere proportion	0.528
Hydrophobicity score	-4.857
Volume score	4.857
Polarity score	10.000
Charge score	7.000
Proportion of polar atoms	41.463
Alpha sphere density	6.007
Cent. of mass - Alpha Sphere max dist	16.279
Flexibility	0.982

Pocket 5

Score	0.047
Druggability Score	0.001
Number of Alpha Spheres	16.000
Total SASA	24.021
Polar SASA	10.737
Apolar SASA	13.284
Volume	89.878
Mean local hydrophobic density	6.000
Mean alpha sphere radius	3.567
Mean alp. sph. solvent access	0.402
Apolar alpha sphere proportion	0.438
Hydrophobicity score	22.143
Volume score	4.571
Polarity score	4.000
Charge score	0.000
Proportion of polar atoms	42.857
Alpha sphere density	1.646
Cent. of mass - Alpha Sphere max dist	3.684
Flexibility	0.663

Pocket 6

Score	0.045
Druggability Score	0.002
Number of Alpha Spheres	26.000
Total SASA	35.891
Polar SASA	12.946

Apolar SASA	22.945
Volume	131.564
Mean local hydrophobic density	6.000
Mean alpha sphere radius	3.700
Mean alp. sph. solvent access	0.421
Apolar alpha sphere proportion	0.269
Hydrophobicity score	23.700
Volume score	4.400
Polarity score	5.000
Charge score	0.000
Proportion of polar atoms	38.889
Alpha sphere density	1.915
Cent. of mass - Alpha Sphere max dist	5.526
Flexibility	0.970

Pocket 7

Score	0.023
Druggability Score	0.001
Number of Alpha Spheres	18.000
Total SASA	40.583
Polar SASA	26.091
Apolar SASA	14.492
Volume	117.018
Mean local hydrophobic density	2.000
Mean alpha sphere radius	3.545
Mean alp. sph. solvent access	0.367
Apolar alpha sphere proportion	0.167
Hydrophobicity score	25.500
Volume score	5.125
Polarity score	5.000
Charge score	3.000
Proportion of polar atoms	52.941
Alpha sphere density	2.061
Cent. of mass - Alpha Sphere max dist	4.677
Flexibility	0.910

Pocket 8

Score	0.010
Druggability Score	0.023
Number of Alpha Spheres	28.000
Total SASA	57.172
Polar SASA	17.320
Apolar SASA	39.852
Volume	182.406
Mean local hydrophobic density	19.000

Mean alpha sphere radius	3.663
Mean alp. sph. solvent access	0.419
Apolar alpha sphere proportion	0.714
Hydrophobicity score	5.714
Volume score	4.429
Polarity score	6.000
Charge score	-1.000
Proportion of polar atoms	38.889
Alpha sphere density	2.417
Cent. of mass - Alpha Sphere max dist	5.293
Flexibility	0.721

Pocket 9

Score	-0.003
Druggability Score	0.001
Number of Alpha Spheres	39.000
Total SASA	120.430
Polar SASA	92.654
Apolar SASA	27.775
Volume	430.069
Mean local hydrophobic density	3.667
Mean alpha sphere radius	3.811
Mean alp. sph. solvent access	0.493
Apolar alpha sphere proportion	0.154
Hydrophobicity score	17.429
Volume score	4.357
Polarity score	8.000
Charge score	1.000
Proportion of polar atoms	53.571
Alpha sphere density	4.628
Cent. of mass - Alpha Sphere max dist	11.598
Flexibility	0.975

Pocket 10

Score	-0.004
Druggability Score	0.002
Number of Alpha Spheres	32.000
Total SASA	99.213
Polar SASA	66.607
Apolar SASA	32.606
Volume	310.538
Mean local hydrophobic density	1.000
Mean alpha sphere radius	3.711
Mean alp. sph. solvent access	0.395
Apolar alpha sphere proportion	0.062
Hydrophobicity score	16.429

	Volume score	4.071
	Polarity score	9.000
	Charge score	0.000
	Proportion of polar atoms	60.000
	Alpha sphere density	3.904
	Cent. of mass - Alpha Sphere max dist	10.958
	Flexibility	0.935
Pocket 11		
	Score	-0.011
	Druggability Score	0.003
	Number of Alpha Spheres	35.000
	Total SASA	64.448
	Polar SASA	25.804
	Apolar SASA	38.644
	Volume	177.237
	Mean local hydrophobic density	4.667
	Mean alpha sphere radius	3.704
	Mean alp. sph. solvent access	0.460
	Apolar alpha sphere proportion	0.171
	Hydrophobicity score	45.462
	Volume score	4.231
	Polarity score	5.000
	Charge score	1.000
	Proportion of polar atoms	47.619
	Alpha sphere density	2.336
	Cent. of mass - Alpha Sphere max dist	7.087
	Flexibility	0.951
Pocket 12		
	Score	-0.012
	Druggability Score	0.004
	Number of Alpha Spheres	39.000
	Total SASA	127.083
	Polar SASA	72.739
	Apolar SASA	54.343
	Volume	336.074
	Mean local hydrophobic density	7.778
	Mean alpha sphere radius	3.932
	Mean alp. sph. solvent access	0.432
	Apolar alpha sphere proportion	0.231
	Hydrophobicity score	23.769
	Volume score	4.385
	Polarity score	8.000
	Charge score	4.000

Proportion of polar atoms	46.154
Alpha sphere density	4.882
Cent. of mass - Alpha Sphere max dist	10.925
Flexibility	0.980

Pocket 13

Score	-0.014
Druggability Score	0.009
Number of Alpha Spheres	25.000
Total SASA	47.080
Polar SASA	9.643
Apolar SASA	37.436
Volume	132.102
Mean local hydrophobic density	15.000
Mean alpha sphere radius	3.775
Mean alp. sph. solvent access	0.442
Apolar alpha sphere proportion	0.640
Hydrophobicity score	11.667
Volume score	4.444
Polarity score	6.000
Charge score	1.000
Proportion of polar atoms	41.176
Alpha sphere density	1.799
Cent. of mass - Alpha Sphere max dist	3.916
Flexibility	0.847

Pocket 14

Score	-0.016
Druggability Score	0.000
Number of Alpha Spheres	22.000
Total SASA	44.894
Polar SASA	29.195
Apolar SASA	15.699
Volume	131.939
Mean local hydrophobic density	1.000
Mean alpha sphere radius	3.725
Mean alp. sph. solvent access	0.487
Apolar alpha sphere proportion	0.091
Hydrophobicity score	6.222
Volume score	3.778
Polarity score	7.000
Charge score	-2.000
Proportion of polar atoms	47.059
Alpha sphere density	1.726
Cent. of mass - Alpha Sphere max dist	3.307
Flexibility	0.662

Pocket 15

Score	-0.019
Druggability Score	0.005
Number of Alpha Spheres	33.000
Total SASA	95.612
Polar SASA	55.760
Apolar SASA	39.852
Volume	341.750
Mean local hydrophobic density	15.000
Mean alpha sphere radius	3.953
Mean alp. sph. solvent access	0.546
Apolar alpha sphere proportion	0.485
Hydrophobicity score	20.273
Volume score	3.909
Polarity score	6.000
Charge score	1.000
Proportion of polar atoms	37.500
Alpha sphere density	3.561
Cent. of mass - Alpha Sphere max dist	8.618
Flexibility	0.955

Pocket 16

Score	-0.019
Druggability Score	0.001
Number of Alpha Spheres	61.000
Total SASA	128.326
Polar SASA	68.994
Apolar SASA	59.332
Volume	425.414
Mean local hydrophobic density	3.000
Mean alpha sphere radius	3.987
Mean alp. sph. solvent access	0.561
Apolar alpha sphere proportion	0.066
Hydrophobicity score	11.417
Volume score	3.500
Polarity score	7.000
Charge score	-1.000
Proportion of polar atoms	45.946
Alpha sphere density	4.254
Cent. of mass - Alpha Sphere max dist	9.492
Flexibility	0.860

Pocket 17

Score	-0.020

Druggability Score	0.000
Number of Alpha Spheres	16.000
Total SASA	23.590
Polar SASA	13.929
Apolar SASA	9.661
Volume	67.593
Mean local hydrophobic density	3.000
Mean alpha sphere radius	3.601
Mean alp. sph. solvent access	0.406
Apolar alpha sphere proportion	0.250
Hydrophobicity score	21.375
Volume score	4.625
Polarity score	6.000
Charge score	-1.000
Proportion of polar atoms	50.000
Alpha sphere density	0.899
Cent. of mass - Alpha Sphere max dist	2.064
Flexibility	0.952

Pocket 18

Score	-0.023
Druggability Score	0.003
Number of Alpha Spheres	41.000
Total SASA	160.190
Polar SASA	93.770
Apolar SASA	66.420
Volume	544.982
Mean local hydrophobic density	5.333
Mean alpha sphere radius	3.970
Mean alp. sph. solvent access	0.566
Apolar alpha sphere proportion	0.220
Hydrophobicity score	8.688
Volume score	3.438
Polarity score	9.000
Charge score	-1.000
Proportion of polar atoms	52.941
Alpha sphere density	6.182
Cent. of mass - Alpha Sphere max dist	16.326
Flexibility	0.683

Pocket 19

Score	-0.025
Druggability Score	0.017
Number of Alpha Spheres	33.000
Total SASA	127.982
Polar SASA	49.486

	Apolar SASA	78.496
	Volume	489.355
	Mean local hydrophobic density	13.000
	Mean alpha sphere radius	3.987
	Mean alp. sph. solvent access	0.524
	Apolar alpha sphere proportion	0.424
	Hydrophobicity score	20.778
	Volume score	2.778
	Polarity score	2.000
	Charge score	2.000
	Proportion of polar atoms	45.833
	Alpha sphere density	4.765
	Cent. of mass - Alpha Sphere max dist	10.461
	Flexibility	0.425
Pocket 20		
	Score	-0.030
	Druggability Score	0.022
	Number of Alpha Spheres	37.000
	Total SASA	90.221
	Polar SASA	43.124
	Apolar SASA	47.097
	Volume	252.489
	Mean local hydrophobic density	11.000
	Mean alpha sphere radius	3.864
	Mean alp. sph. solvent access	0.429
	Apolar alpha sphere proportion	0.324
	Hydrophobicity score	-1.231
	Volume score	3.154
	Polarity score	9.000
	Charge score	-2.000
	Proportion of polar atoms	54.167
	Alpha sphere density	3.172
	Cent. of mass - Alpha Sphere max dist	8.628
	Flexibility	0.921
Pocket 21		
	Score	-0.031
	Druggability Score	0.002
	Number of Alpha Spheres	22.000
	Total SASA	54.209
	Polar SASA	22.810
	Apolar SASA	31.398
	Volume	174.297
	Mean local hydrophobic density	7.000

Mean alpha sphere radius	3.758
Mean alp. sph. solvent access	0.463
Apolar alpha sphere proportion	0.364
Hydrophobicity score	12.500
Volume score	4.375
Polarity score	5.000
Charge score	3.000
Proportion of polar atoms	47.059
Alpha sphere density	1.982
Cent. of mass - Alpha Sphere max dist	5.074
Flexibility	0.959

Pocket 22

Score	-0.031
Druggability Score	0.011
Number of Alpha Spheres	25.000
Total SASA	130.159
Polar SASA	55.286
Apolar SASA	74.873
Volume	428.973
Mean local hydrophobic density	8.000
Mean alpha sphere radius	4.123
Mean alp. sph. solvent access	0.613
Apolar alpha sphere proportion	0.400
Hydrophobicity score	9.556
Volume score	3.444
Polarity score	4.000
Charge score	3.000
Proportion of polar atoms	41.667
Alpha sphere density	5.111
Cent. of mass - Alpha Sphere max dist	12.705
Flexibility	0.831

Pocket 23

Score	-0.035
Druggability Score	0.000
Number of Alpha Spheres	27.000
Total SASA	121.655
Polar SASA	72.142
Apolar SASA	49.513
Volume	507.733
Mean local hydrophobic density	0.000
Mean alpha sphere radius	4.125
Mean alp. sph. solvent access	0.597
Apolar alpha sphere proportion	0.037
Hydrophobicity score	10.111

	Volume score	4.111
	Polarity score	7.000
	Charge score	-2.000
	Proportion of polar atoms	56.522
	Alpha sphere density	4.643
	Cent. of mass - Alpha Sphere max dist	12.729
	Flexibility	0.904
Pocket 24		
	Score	-0.041
	Druggability Score	0.010
	Number of Alpha Spheres	20.000
	Total SASA	83.737
	Polar SASA	29.394
	Apolar SASA	54.343
	Volume	323.576
	Mean local hydrophobic density	13.733
	Mean alpha sphere radius	4.132
	Mean alp. sph. solvent access	0.691
	Apolar alpha sphere proportion	0.750
	Hydrophobicity score	54.000
	Volume score	6.000
	Polarity score	3.000
	Charge score	1.000
	Proportion of polar atoms	33.333
	Alpha sphere density	3.203
	Cent. of mass - Alpha Sphere max dist	8.473
	Flexibility	0.579
Pocket 25		
	Score	-0.042
	Druggability Score	0.000
	Number of Alpha Spheres	15.000
	Total SASA	71.437
	Polar SASA	55.738
	Apolar SASA	15.699
	Volume	173.548
	Mean local hydrophobic density	0.000
	Mean alpha sphere radius	3.690
	Mean alp. sph. solvent access	0.641
	Apolar alpha sphere proportion	0.000
	Hydrophobicity score	-4.000
	Volume score	3.400
	Polarity score	3.000
	Charge score	1.000

Proportion of polar atoms	53.333
Alpha sphere density	2.763
Cent. of mass - Alpha Sphere max dist	5.657
Flexibility	0.045

Pocket 26

Score	-0.045
Druggability Score	0.000
Number of Alpha Spheres	15.000
Total SASA	87.649
Polar SASA	55.043
Apolar SASA	32.606
Volume	307.814
Mean local hydrophobic density	0.000
Mean alpha sphere radius	3.923
Mean alp. sph. solvent access	0.480
Apolar alpha sphere proportion	0.067
Hydrophobicity score	-16.500
Volume score	4.100
Polarity score	8.000
Charge score	0.000
Proportion of polar atoms	68.750
Alpha sphere density	3.426
Cent. of mass - Alpha Sphere max dist	7.776
Flexibility	0.805

Pocket 27

Score	-0.046
Druggability Score	0.001
Number of Alpha Spheres	15.000
Total SASA	65.284
Polar SASA	31.471
Apolar SASA	33.814
Volume	186.697
Mean local hydrophobic density	6.000
Mean alpha sphere radius	3.805
Mean alp. sph. solvent access	0.609
Apolar alpha sphere proportion	0.467
Hydrophobicity score	13.333
Volume score	5.000
Polarity score	3.000
Charge score	1.000
Proportion of polar atoms	35.294
Alpha sphere density	2.477
Cent. of mass - Alpha Sphere max dist	6.343
Flexibility	0.997

Pocket 28

Score	-0.048
Druggability Score	0.417
Number of Alpha Spheres	69.000
Total SASA	155.534
Polar SASA	61.339
Apolar SASA	94.195
Volume	467.668
Mean local hydrophobic density	31.029
Mean alpha sphere radius	3.908
Mean alp. sph. solvent access	0.496
Apolar alpha sphere proportion	0.507
Hydrophobicity score	10.000
Volume score	3.867
Polarity score	8.000
Charge score	1.000
Proportion of polar atoms	34.146
Alpha sphere density	4.860
Cent. of mass - Alpha Sphere max dist	10.044
Flexibility	0.938

Pocket 29

Score	-0.048
Druggability Score	0.018
Number of Alpha Spheres	53.000
Total SASA	127.865
Polar SASA	51.784
Apolar SASA	76.081
Volume	424.183
Mean local hydrophobic density	12.714
Mean alpha sphere radius	3.896
Mean alp. sph. solvent access	0.467
Apolar alpha sphere proportion	0.264
Hydrophobicity score	-7.750
Volume score	3.917
Polarity score	9.000
Charge score	1.000
Proportion of polar atoms	43.750
Alpha sphere density	4.109
Cent. of mass - Alpha Sphere max dist	12.084
Flexibility	0.800

Pocket 30

Score	-0.053

Druggability Score	0.000
Number of Alpha Spheres	17.000
Total SASA	48.653
Polar SASA	28.123
Apolar SASA	20.530
Volume	185.780
Mean local hydrophobic density	6.000
Mean alpha sphere radius	3.997
Mean alp. sph. solvent access	0.631
Apolar alpha sphere proportion	0.412
Hydrophobicity score	13.500
Volume score	5.167
Polarity score	5.000
Charge score	1.000
Proportion of polar atoms	46.154
Alpha sphere density	1.611
Cent. of mass - Alpha Sphere max dist	3.107
Flexibility	0.887

Pocket 31

Score	-0.065
Druggability Score	0.000
Number of Alpha Spheres	37.000
Total SASA	118.953
Polar SASA	68.232
Apolar SASA	50.720
Volume	407.559
Mean local hydrophobic density	3.000
Mean alpha sphere radius	3.961
Mean alp. sph. solvent access	0.574
Apolar alpha sphere proportion	0.108
Hydrophobicity score	-5.385
Volume score	4.615
Polarity score	12.000
Charge score	4.000
Proportion of polar atoms	62.069
Alpha sphere density	3.982
Cent. of mass - Alpha Sphere max dist	11.189
Flexibility	0.868

Pocket 32

Score	-0.069
Druggability Score	0.007
Number of Alpha Spheres	30.000
Total SASA	103.317
Polar SASA	35.690

Apolar SASA	67.627
Volume	311.939
Mean local hydrophobic density	10.667
Mean alpha sphere radius	3.863
Mean alp. sph. solvent access	0.514
Apolar alpha sphere proportion	0.400
Hydrophobicity score	13.455
Volume score	3.545
Polarity score	3.000
Charge score	0.000
Proportion of polar atoms	37.500
Alpha sphere density	3.418
Cent. of mass - Alpha Sphere max dist	8.972
Flexibility	0.532

Pocket 33

Score	-0.069
Druggability Score	0.001
Number of Alpha Spheres	19.000
Total SASA	69.871
Polar SASA	31.227
Apolar SASA	38.644
Volume	228.409
Mean local hydrophobic density	6.000
Mean alpha sphere radius	3.936
Mean alp. sph. solvent access	0.537
Apolar alpha sphere proportion	0.368
Hydrophobicity score	23.167
Volume score	3.500
Polarity score	3.000
Charge score	0.000
Proportion of polar atoms	43.750
Alpha sphere density	2.306
Cent. of mass - Alpha Sphere max dist	5.844
Flexibility	0.697

Pocket 34

Score	-0.083
Druggability Score	0.002
Number of Alpha Spheres	19.000
Total SASA	62.900
Polar SASA	22.810
Apolar SASA	40.089
Volume	216.789
Mean local hydrophobic density	11.000

Mean alpha sphere radius	3.956
Mean alp. sph. solvent access	0.533
Apolar alpha sphere proportion	0.632
Hydrophobicity score	38.333
Volume score	4.000
Polarity score	2.000
Charge score	1.000
Proportion of polar atoms	35.714
Alpha sphere density	1.881
Cent. of mass - Alpha Sphere max dist	5.156
Flexibility	0.584

Pocket 35

Score	-0.084
Druggability Score	0.005
Number of Alpha Spheres	58.000
Total SASA	160.254
Polar SASA	116.780
Apolar SASA	43.475
Volume	456.786
Mean local hydrophobic density	5.750
Mean alpha sphere radius	3.785
Mean alp. sph. solvent access	0.475
Apolar alpha sphere proportion	0.138
Hydrophobicity score	6.059
Volume score	4.059
Polarity score	11.000
Charge score	2.000
Proportion of polar atoms	56.098
Alpha sphere density	4.854
Cent. of mass - Alpha Sphere max dist	14.097
Flexibility	0.833

Pocket 36

Score	-0.094
Druggability Score	0.000
Number of Alpha Spheres	15.000
Total SASA	76.028
Polar SASA	39.799
Apolar SASA	36.229
Volume	219.278
Mean local hydrophobic density	0.000
Mean alpha sphere radius	4.016
Mean alp. sph. solvent access	0.546
Apolar alpha sphere proportion	0.067
Hydrophobicity score	11.714

Volume score	3.429
Polarity score	4.000
Charge score	0.000
Proportion of polar atoms	57.143
Alpha sphere density	2.401
Cent. of mass - Alpha Sphere max dist	5.719
Flexibility	0.544

Pocket 37

Score	-0.098
Druggability Score	0.000
Number of Alpha Spheres	16.000
Total SASA	87.516
Polar SASA	54.910
Apolar SASA	32.606
Volume	254.181
Mean local hydrophobic density	1.000
Mean alpha sphere radius	3.871
Mean alp. sph. solvent access	0.588
Apolar alpha sphere proportion	0.125
Hydrophobicity score	-4.714
Volume score	4.000
Polarity score	6.000
Charge score	1.000
Proportion of polar atoms	53.333
Alpha sphere density	2.837
Cent. of mass - Alpha Sphere max dist	7.218
Flexibility	0.814

Pocket 38

Score	-0.098
Druggability Score	0.222
Number of Alpha Spheres	60.000
Total SASA	162.716
Polar SASA	81.488
Apolar SASA	81.228
Volume	633.485
Mean local hydrophobic density	29.355
Mean alpha sphere radius	3.915
Mean alp. sph. solvent access	0.521
Apolar alpha sphere proportion	0.517
Hydrophobicity score	22.812
Volume score	4.625
Polarity score	9.000
Charge score	3.000

Proportion of polar atoms	37.209
Alpha sphere density	4.558
Cent. of mass - Alpha Sphere max dist	10.708
Flexibility	0.733

Pocket 39

Score	-0.099
Druggability Score	0.000
Number of Alpha Spheres	23.000
Total SASA	78.389
Polar SASA	54.237
Apolar SASA	24.153
Volume	209.590
Mean local hydrophobic density	1.000
Mean alpha sphere radius	3.890
Mean alp. sph. solvent access	0.492
Apolar alpha sphere proportion	0.087
Hydrophobicity score	26.714
Volume score	4.286
Polarity score	5.000
Charge score	1.000
Proportion of polar atoms	58.824
Alpha sphere density	2.264
Cent. of mass - Alpha Sphere max dist	5.355
Flexibility	0.970

Pocket 40

Score	-0.101
Druggability Score	0.000
Number of Alpha Spheres	16.000
Total SASA	79.534
Polar SASA	52.966
Apolar SASA	26.568
Volume	190.175
Mean local hydrophobic density	2.000
Mean alpha sphere radius	3.898
Mean alp. sph. solvent access	0.512
Apolar alpha sphere proportion	0.188
Hydrophobicity score	15.833
Volume score	5.000
Polarity score	5.000
Charge score	2.000
Proportion of polar atoms	60.000
Alpha sphere density	2.471
Cent. of mass - Alpha Sphere max dist	5.287
Flexibility	0.462

Pocket 41

Score	-0.106
Druggability Score	0.014
Number of Alpha Spheres	25.000
Total SASA	87.337
Polar SASA	18.502
Apolar SASA	68.835
Volume	282.314
Mean local hydrophobic density	18.000
Mean alpha sphere radius	4.009
Mean alp. sph. solvent access	0.608
Apolar alpha sphere proportion	0.760
Hydrophobicity score	36.200
Volume score	5.000
Polarity score	3.000
Charge score	0.000
Proportion of polar atoms	23.810
Alpha sphere density	2.544
Cent. of mass - Alpha Sphere max dist	6.396
Flexibility	0.903

Pocket 42

Score	-0.112
Druggability Score	0.017
Number of Alpha Spheres	43.000
Total SASA	121.716
Polar SASA	51.673
Apolar SASA	70.042
Volume	411.515
Mean local hydrophobic density	14.000
Mean alpha sphere radius	4.059
Mean alp. sph. solvent access	0.509
Apolar alpha sphere proportion	0.349
Hydrophobicity score	20.500
Volume score	4.000
Polarity score	8.000
Charge score	1.000
Proportion of polar atoms	44.828
Alpha sphere density	3.466
Cent. of mass - Alpha Sphere max dist	9.818
Flexibility	0.942

Pocket 43

Score	-0.113

Druggability Score	0.001
Number of Alpha Spheres	44.000
Total SASA	136.831
Polar SASA	76.450
Apolar SASA	60.381
Volume	464.167
Mean local hydrophobic density	9.000
Mean alpha sphere radius	3.956
Mean alp. sph. solvent access	0.535
Apolar alpha sphere proportion	0.227
Hydrophobicity score	-4.200
Volume score	4.500
Polarity score	8.000
Charge score	0.000
Proportion of polar atoms	48.485
Alpha sphere density	4.064
Cent. of mass - Alpha Sphere max dist	12.052
Flexibility	0.928

Pocket 44

Score	-0.118
Druggability Score	0.187
Number of Alpha Spheres	41.000
Total SASA	155.589
Polar SASA	54.148
Apolar SASA	101.441
Volume	530.394
Mean local hydrophobic density	22.160
Mean alpha sphere radius	3.940
Mean alp. sph. solvent access	0.509
Apolar alpha sphere proportion	0.610
Hydrophobicity score	7.100
Volume score	4.300
Polarity score	8.000
Charge score	2.000
Proportion of polar atoms	35.484
Alpha sphere density	4.749
Cent. of mass - Alpha Sphere max dist	12.554
Flexibility	0.967

Pocket 45

Score	-0.121
Druggability Score	0.002
Number of Alpha Spheres	15.000
Total SASA	115.627
Polar SASA	54.038

	Apolar SASA	61.589
	Volume	349.232
	Mean local hydrophobic density	8.000
	Mean alpha sphere radius	4.095
	Mean alp. sph. solvent access	0.698
	Apolar alpha sphere proportion	0.600
	Hydrophobicity score	5.750
	Volume score	4.500
	Polarity score	5.000
	Charge score	1.000
	Proportion of polar atoms	43.478
	Alpha sphere density	3.838
	Cent. of mass - Alpha Sphere max dist	8.174
	Flexibility	0.959
Pocket 46		
	Score	-0.121
	Druggability Score	0.000
	Number of Alpha Spheres	21.000
	Total SASA	92.135
	Polar SASA	36.585
	Apolar SASA	55.551
	Volume	259.709
	Mean local hydrophobic density	1.000
	Mean alpha sphere radius	3.924
	Mean alp. sph. solvent access	0.573
	Apolar alpha sphere proportion	0.095
	Hydrophobicity score	3.625
	Volume score	3.125
	Polarity score	4.000
	Charge score	-2.000
	Proportion of polar atoms	47.368
	Alpha sphere density	2.617
	Cent. of mass - Alpha Sphere max dist	5.316
	Flexibility	0.577
Pocket 47		
	Score	-0.129
	Druggability Score	0.003
	Number of Alpha Spheres	20.000
	Total SASA	81.657
	Polar SASA	32.144
	Apolar SASA	49.513
	Volume	192.281
	Mean local hydrophobic density	12.000

Mean alpha sphere radius	3.802
Mean alp. sph. solvent access	0.507
Apolar alpha sphere proportion	0.650
Hydrophobicity score	20.400
Volume score	3.200
Polarity score	1.000
Charge score	-1.000
Proportion of polar atoms	33.333
Alpha sphere density	2.173
Cent. of mass - Alpha Sphere max dist	4.736
Flexibility	0.882

Pocket 48

Score	-0.132
Druggability Score	0.008
Number of Alpha Spheres	28.000
Total SASA	117.142
Polar SASA	59.196
Apolar SASA	57.946
Volume	305.573
Mean local hydrophobic density	11.000
Mean alpha sphere radius	3.982
Mean alp. sph. solvent access	0.650
Apolar alpha sphere proportion	0.429
Hydrophobicity score	19.200
Volume score	3.800
Polarity score	5.000
Charge score	2.000
Proportion of polar atoms	44.000
Alpha sphere density	3.415
Cent. of mass - Alpha Sphere max dist	10.477
Flexibility	0.926

Pocket 49

Score	-0.132
Druggability Score	0.006
Number of Alpha Spheres	23.000
Total SASA	81.444
Polar SASA	36.762
Apolar SASA	44.682
Volume	185.406
Mean local hydrophobic density	15.000
Mean alpha sphere radius	3.930
Mean alp. sph. solvent access	0.476
Apolar alpha sphere proportion	0.696
Hydrophobicity score	38.333

Volume score	4.444
Polarity score	3.000
Charge score	1.000
Proportion of polar atoms	35.294
Alpha sphere density	2.043
Cent. of mass - Alpha Sphere max dist	5.135
Flexibility	0.930

Pocket 50

Score	-0.140
Druggability Score	0.013
Number of Alpha Spheres	49.000
Total SASA	193.174
Polar SASA	82.073
Apolar SASA	111.102
Volume	596.227
Mean local hydrophobic density	17.895
Mean alpha sphere radius	3.911
Mean alp. sph. solvent access	0.577
Apolar alpha sphere proportion	0.388
Hydrophobicity score	18.867
Volume score	3.867
Polarity score	8.000
Charge score	-1.000
Proportion of polar atoms	44.737
Alpha sphere density	5.646
Cent. of mass - Alpha Sphere max dist	12.261
Flexibility	0.862

Pocket 51

Score	-0.143
Druggability Score	0.000
Number of Alpha Spheres	22.000
Total SASA	108.236
Polar SASA	35.779
Apolar SASA	72.458
Volume	342.493
Mean local hydrophobic density	4.000
Mean alpha sphere radius	4.103
Mean alp. sph. solvent access	0.511
Apolar alpha sphere proportion	0.227
Hydrophobicity score	12.571
Volume score	4.571
Polarity score	3.000
Charge score	1.000

Proportion of polar atoms	41.176
Alpha sphere density	3.105
Cent. of mass - Alpha Sphere max dist	6.645
Flexibility	0.799

Pocket 52

Score	-0.146
Druggability Score	0.000
Number of Alpha Spheres	39.000
Total SASA	139.074
Polar SASA	71.447
Apolar SASA	67.627
Volume	411.859
Mean local hydrophobic density	5.000
Mean alpha sphere radius	3.842
Mean alp. sph. solvent access	0.438
Apolar alpha sphere proportion	0.154
Hydrophobicity score	12.929
Volume score	3.714
Polarity score	8.000
Charge score	0.000
Proportion of polar atoms	51.852
Alpha sphere density	3.832
Cent. of mass - Alpha Sphere max dist	7.323
Flexibility	0.923

Pocket 53

Score	-0.147
Druggability Score	0.000
Number of Alpha Spheres	32.000
Total SASA	125.518
Polar SASA	84.459
Apolar SASA	41.059
Volume	345.878
Mean local hydrophobic density	2.000
Mean alpha sphere radius	3.802
Mean alp. sph. solvent access	0.469
Apolar alpha sphere proportion	0.094
Hydrophobicity score	1.182
Volume score	4.364
Polarity score	8.000
Charge score	3.000
Proportion of polar atoms	54.167
Alpha sphere density	3.489
Cent. of mass - Alpha Sphere max dist	7.513
Flexibility	0.814

Pocket 54

Score	-0.153
Druggability Score	0.000
Number of Alpha Spheres	20.000
Total SASA	96.633
Polar SASA	59.771
Apolar SASA	36.862
Volume	238.236
Mean local hydrophobic density	1.000
Mean alpha sphere radius	3.906
Mean alp. sph. solvent access	0.597
Apolar alpha sphere proportion	0.100
Hydrophobicity score	3.833
Volume score	3.833
Polarity score	5.000
Charge score	1.000
Proportion of polar atoms	58.824
Alpha sphere density	2.530
Cent. of mass - Alpha Sphere max dist	4.823
Flexibility	0.718

Pocket 55

Score	-0.154
Druggability Score	0.006
Number of Alpha Spheres	40.000
Total SASA	138.765
Polar SASA	82.006
Apolar SASA	56.759
Volume	505.517
Mean local hydrophobic density	15.000
Mean alpha sphere radius	4.094
Mean alp. sph. solvent access	0.447
Apolar alpha sphere proportion	0.400
Hydrophobicity score	4.556
Volume score	4.111
Polarity score	7.000
Charge score	-1.000
Proportion of polar atoms	43.333
Alpha sphere density	3.685
Cent. of mass - Alpha Sphere max dist	8.359
Flexibility	0.968

Pocket 56

Score	-0.159

Druggability Score	0.007
Number of Alpha Spheres	40.000
Total SASA	167.935
Polar SASA	64.079
Apolar SASA	103.856
Volume	496.651
Mean local hydrophobic density	15.143
Mean alpha sphere radius	3.871
Mean alp. sph. solvent access	0.546
Apolar alpha sphere proportion	0.525
Hydrophobicity score	26.900
Volume score	5.200
Polarity score	7.000
Charge score	2.000
Proportion of polar atoms	32.258
Alpha sphere density	4.927
Cent. of mass - Alpha Sphere max dist	9.689
Flexibility	0.860

Pocket 57

Score	-0.173
Druggability Score	0.003
Number of Alpha Spheres	38.000
Total SASA	126.095
Polar SASA	76.583
Apolar SASA	49.513
Volume	348.897
Mean local hydrophobic density	12.000
Mean alpha sphere radius	4.140
Mean alp. sph. solvent access	0.409
Apolar alpha sphere proportion	0.342
Hydrophobicity score	10.556
Volume score	4.222
Polarity score	8.000
Charge score	0.000
Proportion of polar atoms	52.381
Alpha sphere density	3.188
Cent. of mass - Alpha Sphere max dist	9.370
Flexibility	0.843

Pocket 58

Score	-0.179
Druggability Score	0.009
Number of Alpha Spheres	75.000
Total SASA	193.565
Polar SASA	107.269

Apolar SASA	86.296
Volume	630.895
Mean local hydrophobic density	18.000
Mean alpha sphere radius	4.004
Mean alp. sph. solvent access	0.491
Apolar alpha sphere proportion	0.253
Hydrophobicity score	-0.571
Volume score	4.143
Polarity score	10.000
Charge score	4.000
Proportion of polar atoms	53.191
Alpha sphere density	4.782
Cent. of mass - Alpha Sphere max dist	12.088
Flexibility	0.895

Pocket 59

Score	-0.194
Druggability Score	0.003
Number of Alpha Spheres	24.000
Total SASA	125.629
Polar SASA	39.887
Apolar SASA	85.742
Volume	347.772
Mean local hydrophobic density	10.000
Mean alpha sphere radius	4.064
Mean alp. sph. solvent access	0.586
Apolar alpha sphere proportion	0.458
Hydrophobicity score	21.000
Volume score	4.250
Polarity score	5.000
Charge score	1.000
Proportion of polar atoms	45.000
Alpha sphere density	3.278
Cent. of mass - Alpha Sphere max dist	8.997
Flexibility	0.899

Pocket 60

Score	-0.209
Druggability Score	0.666t
Number of Alpha Spheres	157.000
Total SASA	436.409
Polar SASA	147.786
Apolar SASA	288.623
Volume	1667.223
Mean local hydrophobic density	20.340

Mean alpha sphere radius	4.062
Mean alp. sph. solvent access	0.507
Apolar alpha sphere proportion	0.299
Hydrophobicity score	27.125
Volume score	4.229
Polarity score	29.000
Charge score	0.000
Proportion of polar atoms	39.796
Alpha sphere density	9.556
Cent. of mass - Alpha Sphere max dist	25.332
Flexibility	0.844

Pocket 61

Score	-0.221
Druggability Score	0.001
Number of Alpha Spheres	19.000
Total SASA	98.818
Polar SASA	25.153
Apolar SASA	73.665
Volume	195.952
Mean local hydrophobic density	10.000
Mean alpha sphere radius	3.903
Mean alp. sph. solvent access	0.542
Apolar alpha sphere proportion	0.579
Hydrophobicity score	43.250
Volume score	5.500
Polarity score	2.000
Charge score	1.000
Proportion of polar atoms	33.333
Alpha sphere density	1.951
Cent. of mass - Alpha Sphere max dist	5.180
Flexibility	0.636

Pocket 62

Score	-0.233
Druggability Score	0.004
Number of Alpha Spheres	46.000
Total SASA	181.659
Polar SASA	91.087
Apolar SASA	90.572
Volume	598.479
Mean local hydrophobic density	14.000
Mean alpha sphere radius	4.154
Mean alp. sph. solvent access	0.523
Apolar alpha sphere proportion	0.326
Hydrophobicity score	8.643

Volume score	4.643
Polarity score	10.000
Charge score	5.000
Proportion of polar atoms	52.941
Alpha sphere density	4.318
Cent. of mass - Alpha Sphere max dist	12.678
Flexibility	0.414

Pocket 63

Score	-0.297
Druggability Score	0.001
Number of Alpha Spheres	233.000
Total SASA	643.190
Polar SASA	320.436
Apolar SASA	322.753
Volume	2208.325
Mean local hydrophobic density	27.644
Mean alpha sphere radius	3.985
Mean alp. sph. solvent access	0.459
Apolar alpha sphere proportion	0.386
Hydrophobicity score	3.833
Volume score	4.119
Polarity score	25.000
Charge score	2.000
Proportion of polar atoms	41.406
Alpha sphere density	10.983
Cent. of mass - Alpha Sphere max dist	26.566
Flexibility	0.588

Pocket 64

Score	-0.328
Druggability Score	0.018
Number of Alpha Spheres	197.000
Total SASA	495.749
Polar SASA	230.070
Apolar SASA	265.678
Volume	1990.677
Mean local hydrophobic density	28.841
Mean alpha sphere radius	3.927
Mean alp. sph. solvent access	0.465
Apolar alpha sphere proportion	0.350
Hydrophobicity score	12.600
Volume score	4.800
Polarity score	23.000
Charge score	5.000

Proportion of polar atoms	39.806
Alpha sphere density	8.845
Cent. of mass - Alpha Sphere max dist	20.290
Flexibility	0.879
